# Supplementary material for: The copy-number events in skull base chordoma stratify tumours into four biologically coherent groups
Source: Neoplasia. 2026 Jun 12;79:101325. doi: 10.1016/j.neo.2026.101325 (PMC13276418; doi:10.1016/j.neo.2026.101325)
Supplement: Supplementary file 1 [file mmc1.docx]

# Supplementary Material


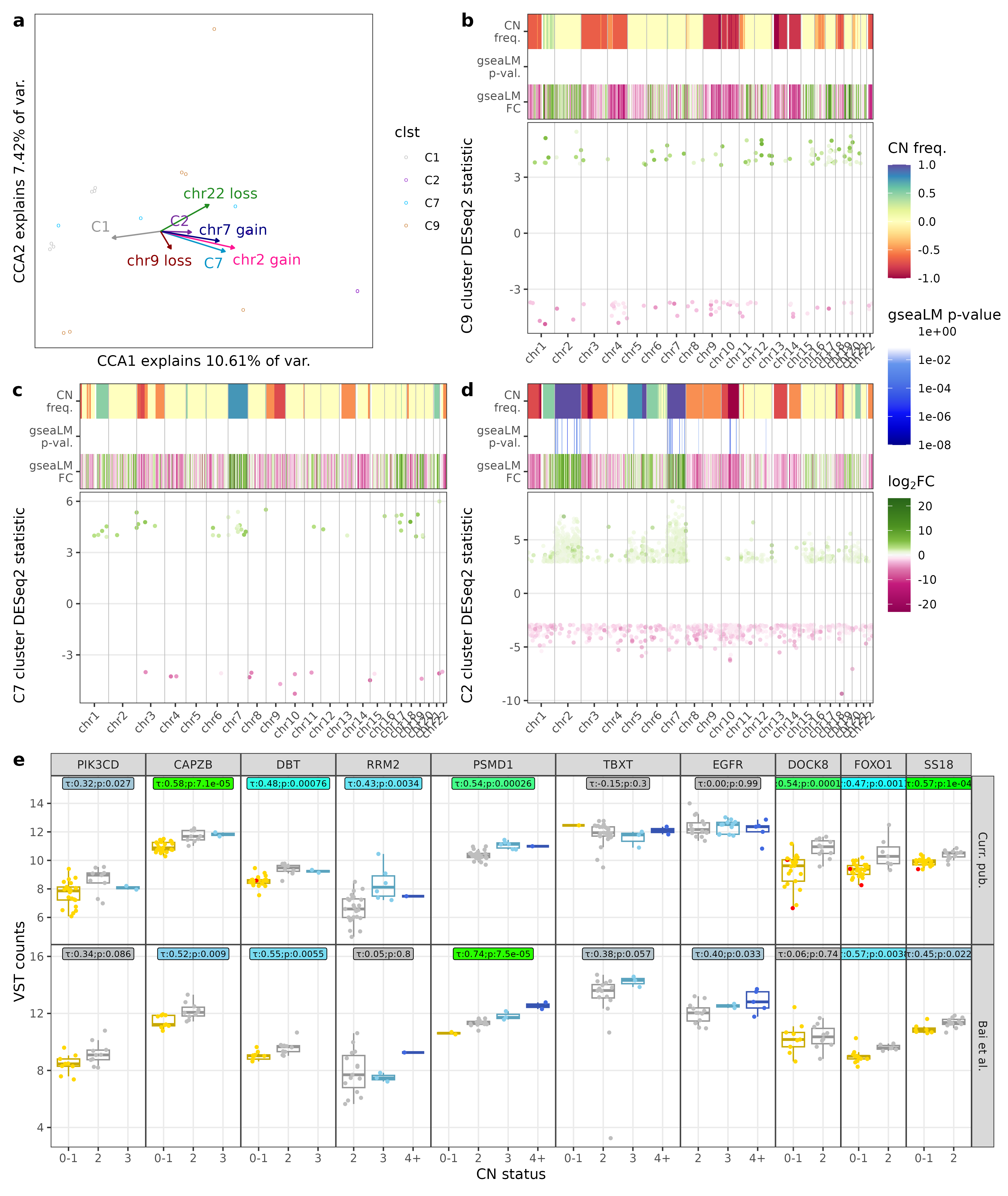


**Supplementary Figure 1.** Copy-number events in the context of RNA-sequencing data; a) canonical componenent analysis of samples from Bai et al.; b) gene expression and CN events in **C9** from Bai et al.; c) gene expression and CN events in **C7** from Bai et al.; d) gene expression and CN events in **C2** from Bai et al.; e) gene expression correlation with CN events for selected genes.
