# Supplementary material for: The copy-number events in skull base chordoma stratify tumours into four biologically coherent groups
Source: Neoplasia. 2026 Jun 12;79:101325. doi: 10.1016/j.neo.2026.101325 (PMC13276418; doi:10.1016/j.neo.2026.101325)
Supplement: Supplementary file 2 [file mmc2.pdf]

11/18

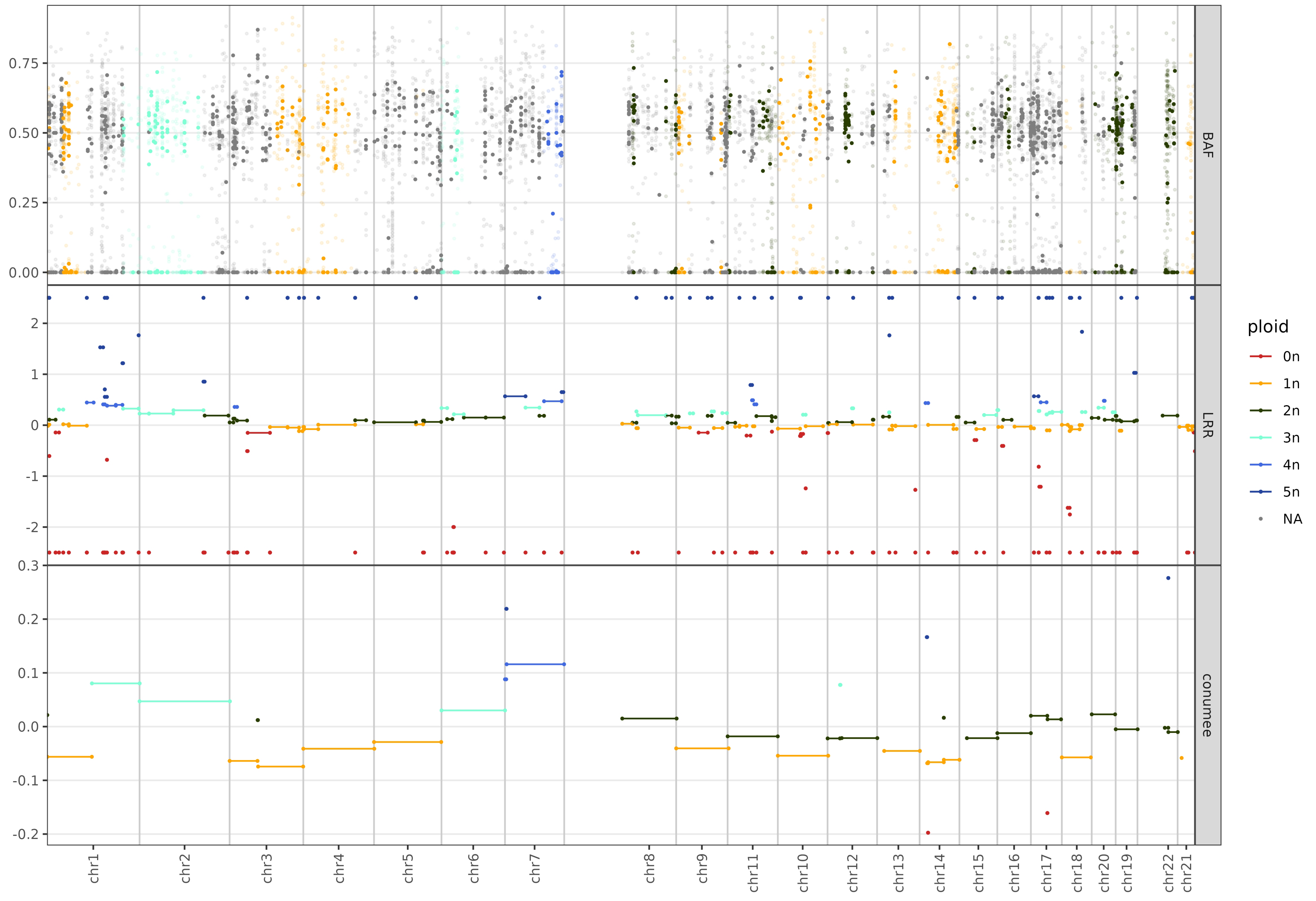

BAF
LRR
conumee
0.75
0.50
0.25
0.00
2
1
0
-1
-2
0.3
0.2
0.1
0.0
-0.1
-0.2
chr1
chr2
chr3
chr4
chr5
chr6
chr7
chr8
chr9
chr11
chr10
chr12
chr13
chr14
chr15
chr16
chr17
chr18
chr20
chr19
chr22
chr21
ploid
0n
1n
2n
3n
4n
5n
NA

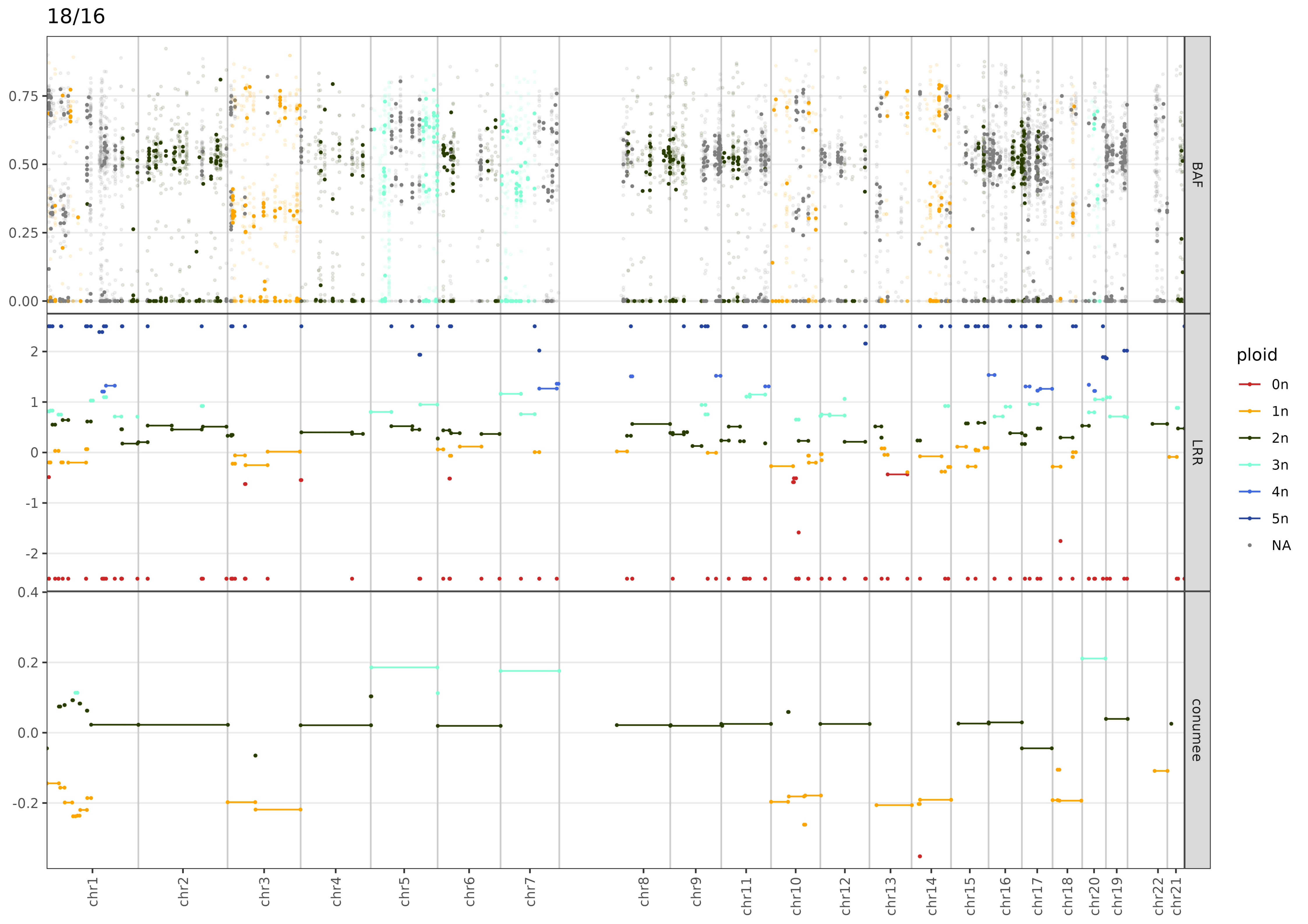

18/16
BAF
LRR
conumee
ploid
0n
1n
2n
3n
4n
5n
NA
0.75
0.50
0.25
0.00
2
1
0
-1
-2
0.4
0.2
0.0
-0.2
chr1
chr2
chr3
chr4
chr5
chr6
chr7
chr8
chr9
chr11
chr10
chr12
chr13
chr14
chr15
chr16
chr17
chr18
chr20
chr19
chr22
chr21

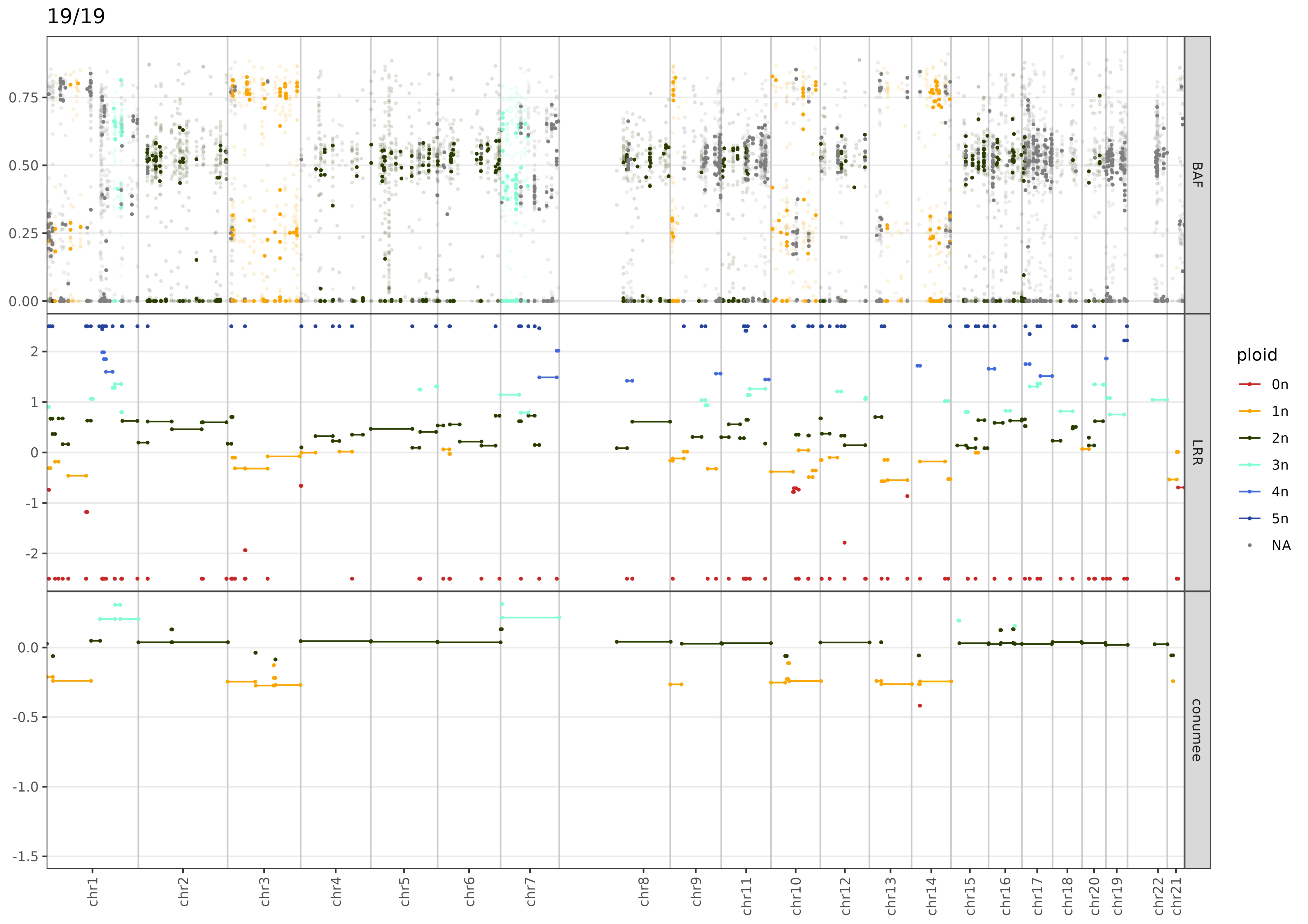
19/19
BAF
LRR
conumee
ploid
0n
1n
2n
3n
4n
5n
NA
chr1
chr2
chr3
chr4
chr5
chr6
chr7
chr8
chr9
chr11
chr10
chr12
chr13
chr14
chr15
chr16
chr17
chr18
chr20
chr19
chr22
chr21

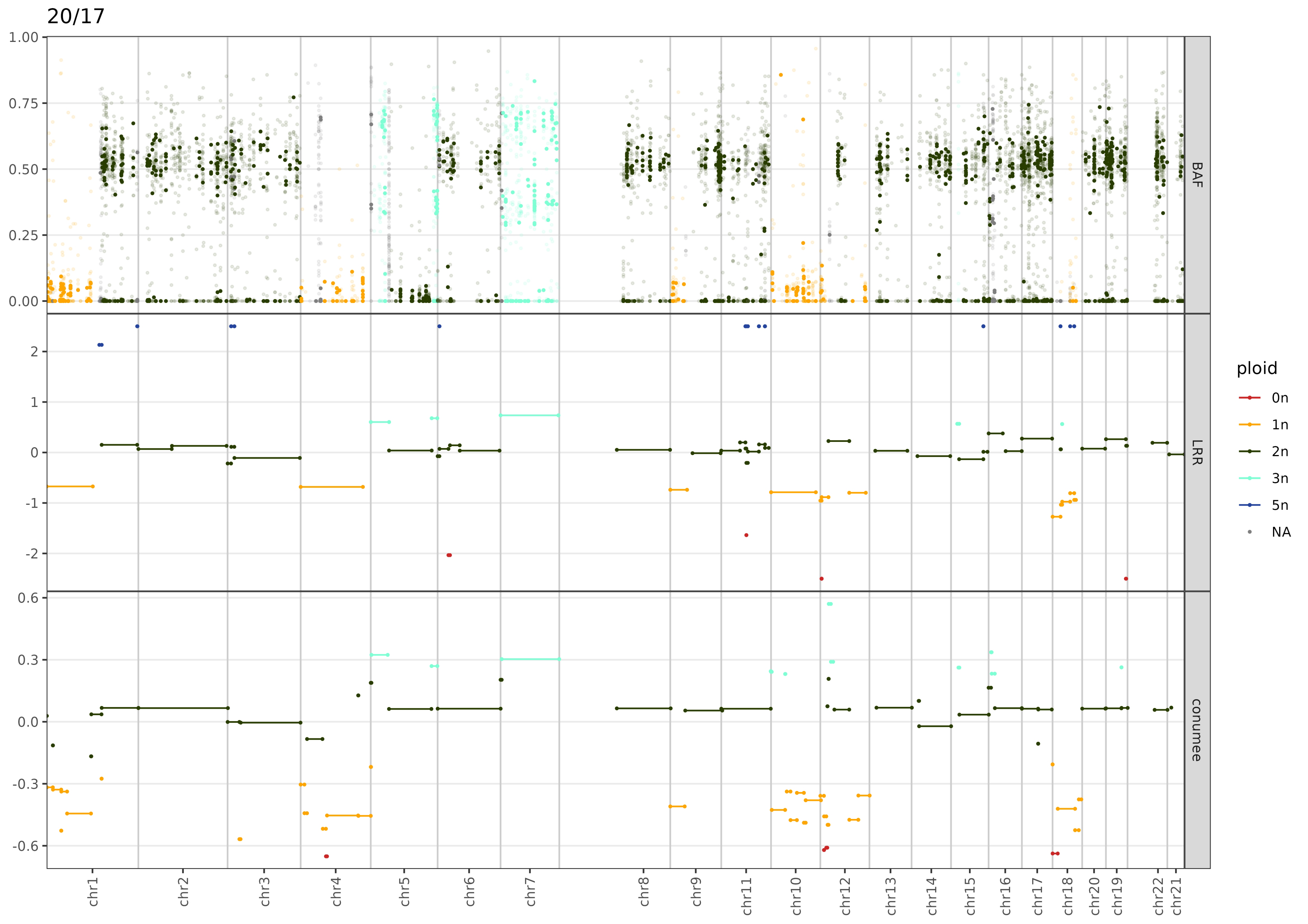

20/17
1.00
0.75
0.50
0.25
0.00
BAF
2
1
0
-1
-2
LRR
0.6
0.3
0.0
-0.3
-0.6
conumee
chr1
chr2
chr3
chr4
chr5
chr6
chr7
chr8
chr9
chr11
chr10
chr12
chr13
chr14
chr15
chr16
chr17
chr18
chr20
chr19
chr22
chr21
ploid
0n
1n
2n
3n
5n
NA

24/18

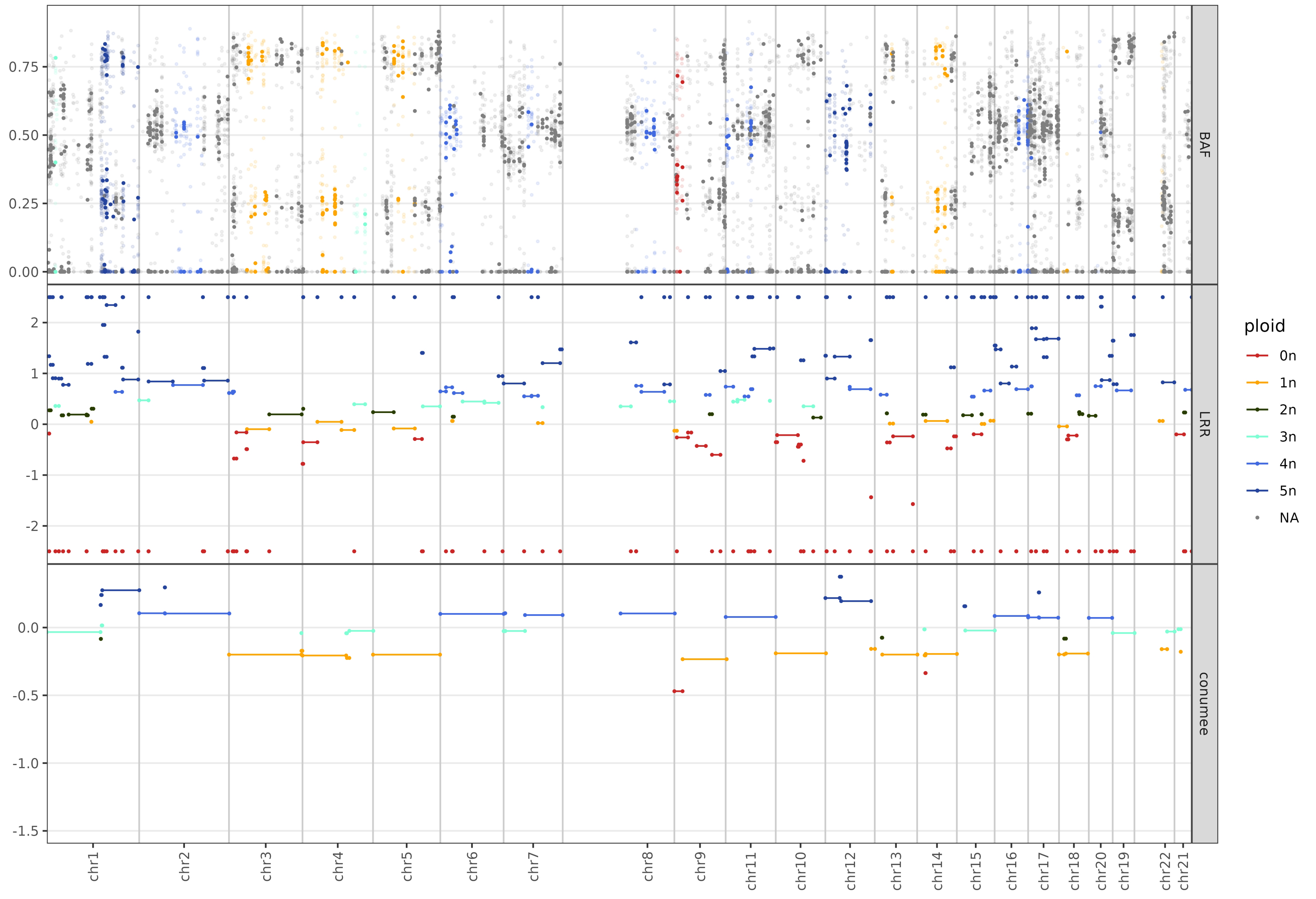

BAF
LRR
conumee
ploid
0n
1n
2n
3n
4n
5n
NA
0.75
0.50
0.25
0.00
2
1
0
-1
-2
0.0
-0.5
-1.0
-1.5
chr1
chr2
chr3
chr4
chr5
chr6
chr7
chr8
chr9
chr11
chr10
chr12
chr13
chr14
chr15
chr16
chr17
chr18
chr20
chr19
chr22
chr21

26/15

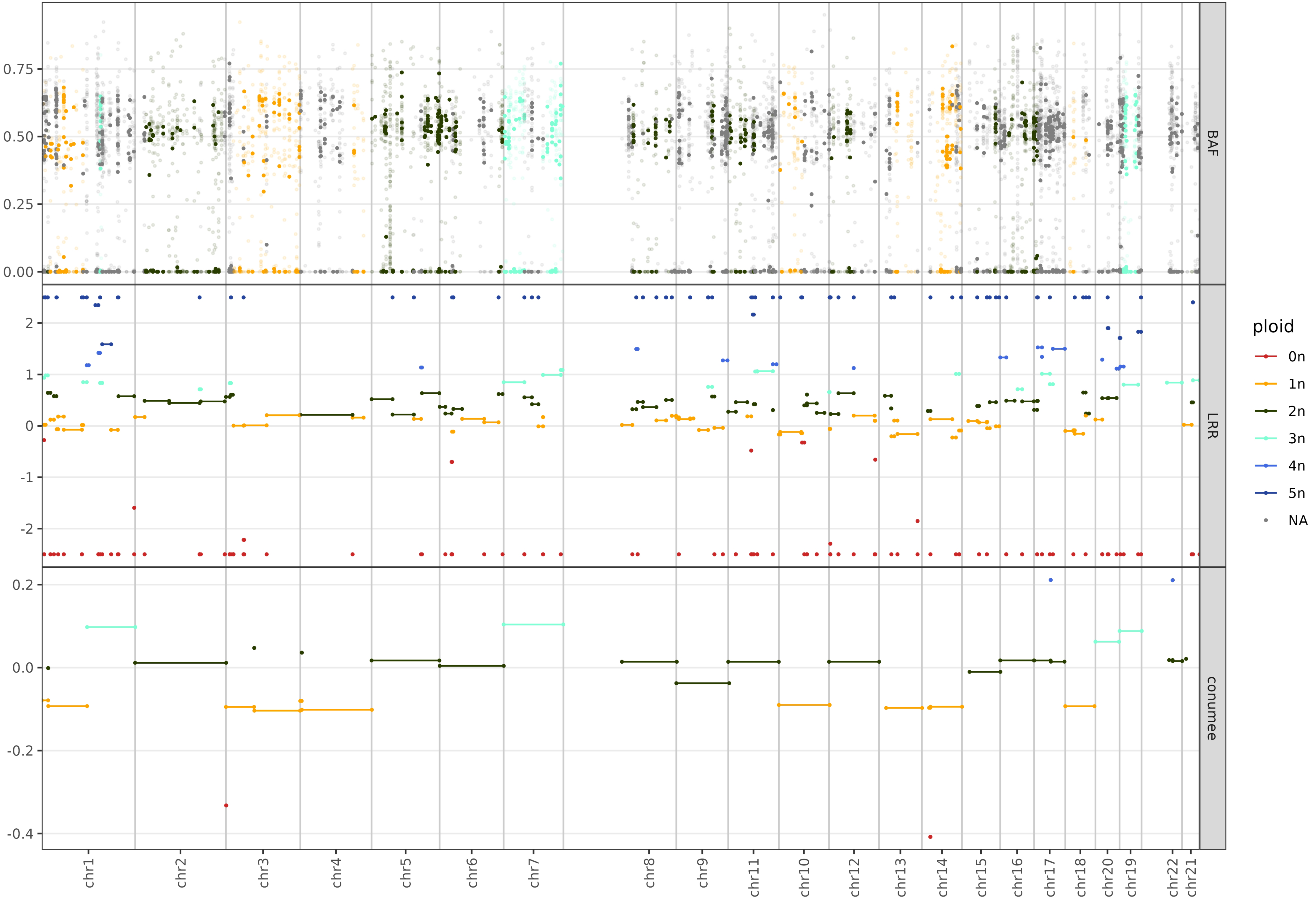

0.75
0.50
0.25
0.00
BAF
2
1
0
-1
-2
LRR
0.2
0.0
-0.2
-0.4
conumee
chr1
chr2
chr3
chr4
chr5
chr6
chr7
chr8
chr9
chr11
chr10
chr12
chr13
chr14
chr15
chr16
chr17
chr18
chr20
chr19
chr22
chr21
ploid
0n
1n
2n
3n
4n
5n
NA

26/16

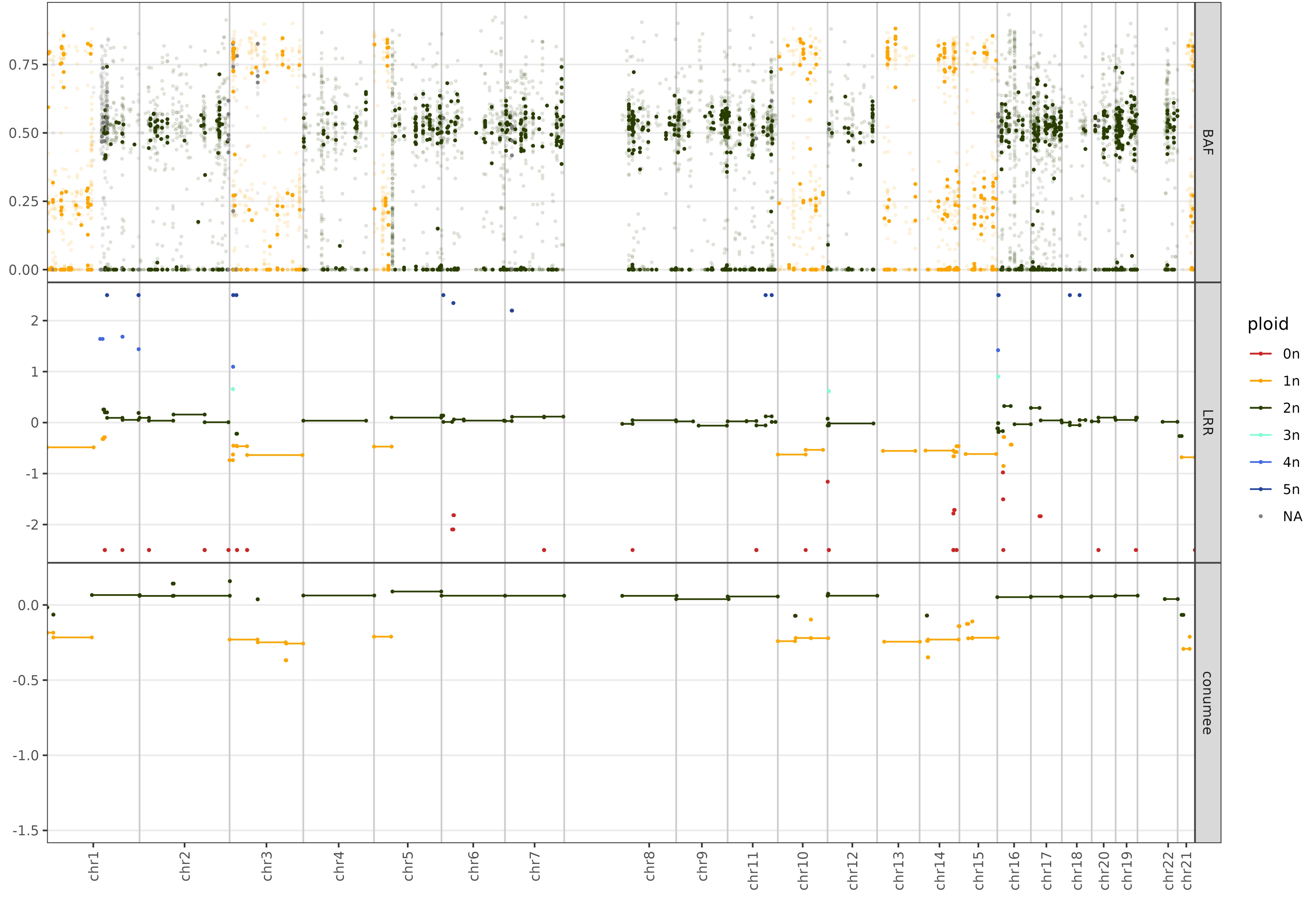
BAF
LRR
conumee
0.75
0.50
0.25
0.00
2
1
0
-1
-2
0.0
-0.5
-1.0
-1.5
chr1
chr2
chr3
chr4
chr5
chr6
chr7
chr8
chr9
chr11
chr10
chr12
chr13
chr14
chr15
chr16
chr17
chr18
chr20
chr19
chr22
chr21
ploid
0n
1n
2n
3n
4n
5n
NA

28/20

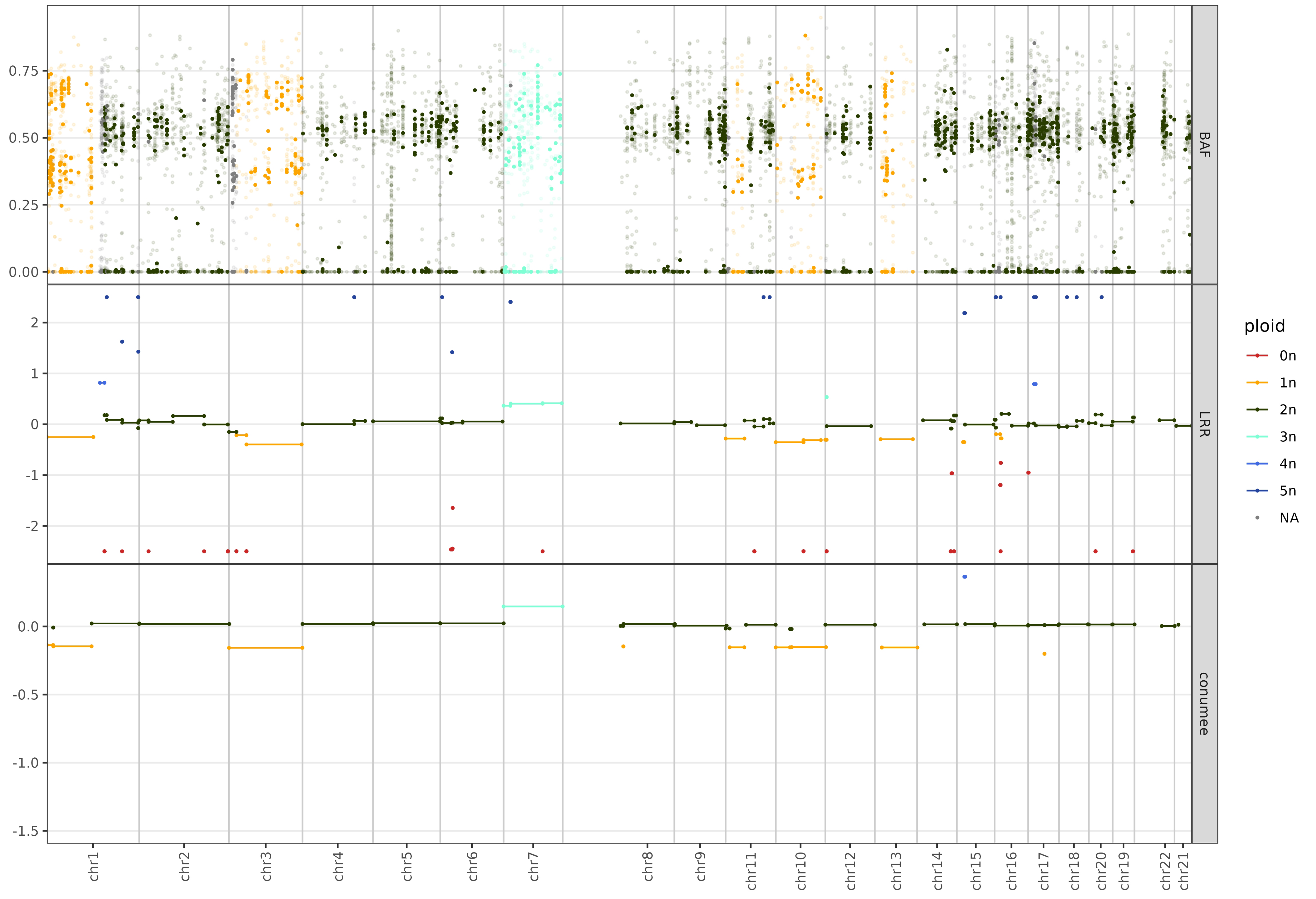

BAF
LRR
conumee
0.75
0.50
0.25
0.00
2
1
0
-1
-2
0.0
-0.5
-1.0
-1.5
chr1
chr2
chr3
chr4
chr5
chr6
chr7
chr8
chr9
chr11
chr10
chr12
chr13
chr14
chr15
chr16
chr17
chr18
chr20
chr19
chr22
chr21
ploid
0n
1n
2n
3n
4n
5n
NA

31/14

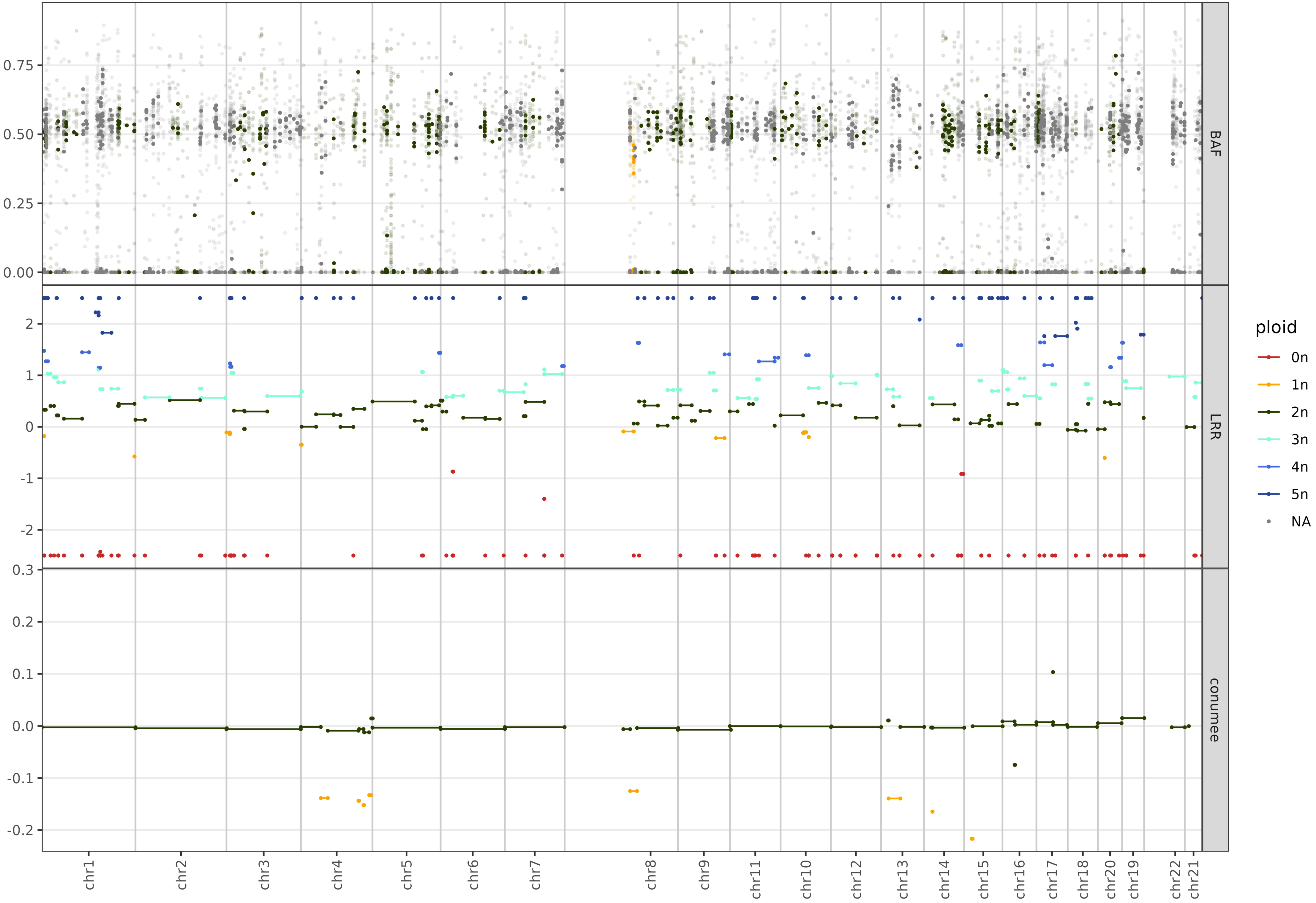

BAF
LRR
conumee
0.75
0.50
0.25
0.00
2
1
0
-1
-2
0.3
0.2
0.1
0.0
-0.1
-0.2
chr1
chr2
chr3
chr4
chr5
chr6
chr7
chr8
chr9
chr11
chr10
chr12
chr13
chr14
chr15
chr16
chr17
chr18
chr20
chr19
chr22
chr21
ploid
0n
1n
2n
3n
4n
5n
NA

31/20

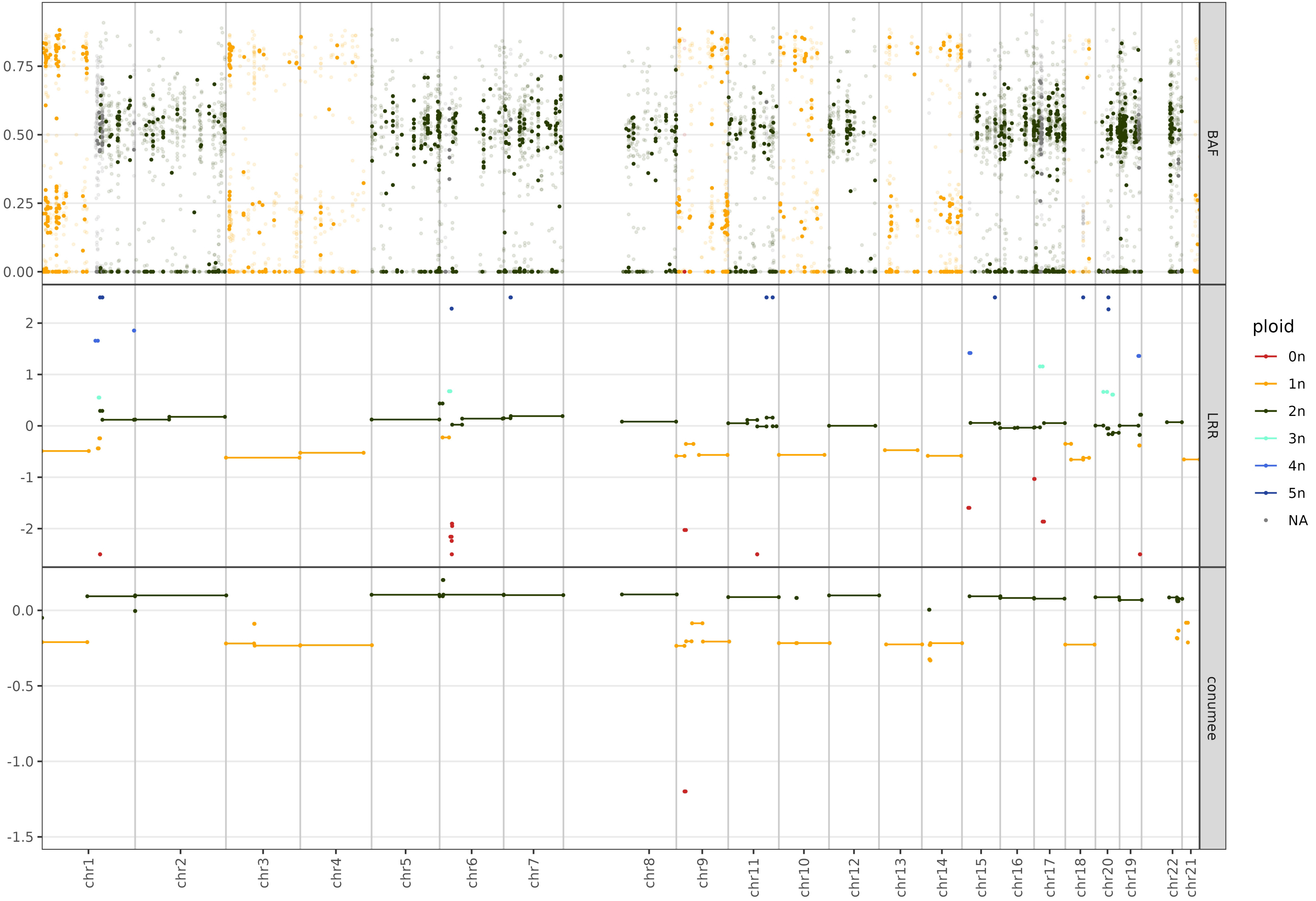
BAF
LRR
conumee
0.75
0.50
0.25
0.00
2
1
0
-1
-2
0.0
-0.5
-1.0
-1.5
chr1
chr2
chr3
chr4
chr5
chr6
chr7
chr8
chr9
chr11
chr10
chr12
chr13
chr14
chr15
chr16
chr17
chr18
chr20
chr19
chr22
chr21
ploid
0n
1n
2n
3n
4n
5n
NA

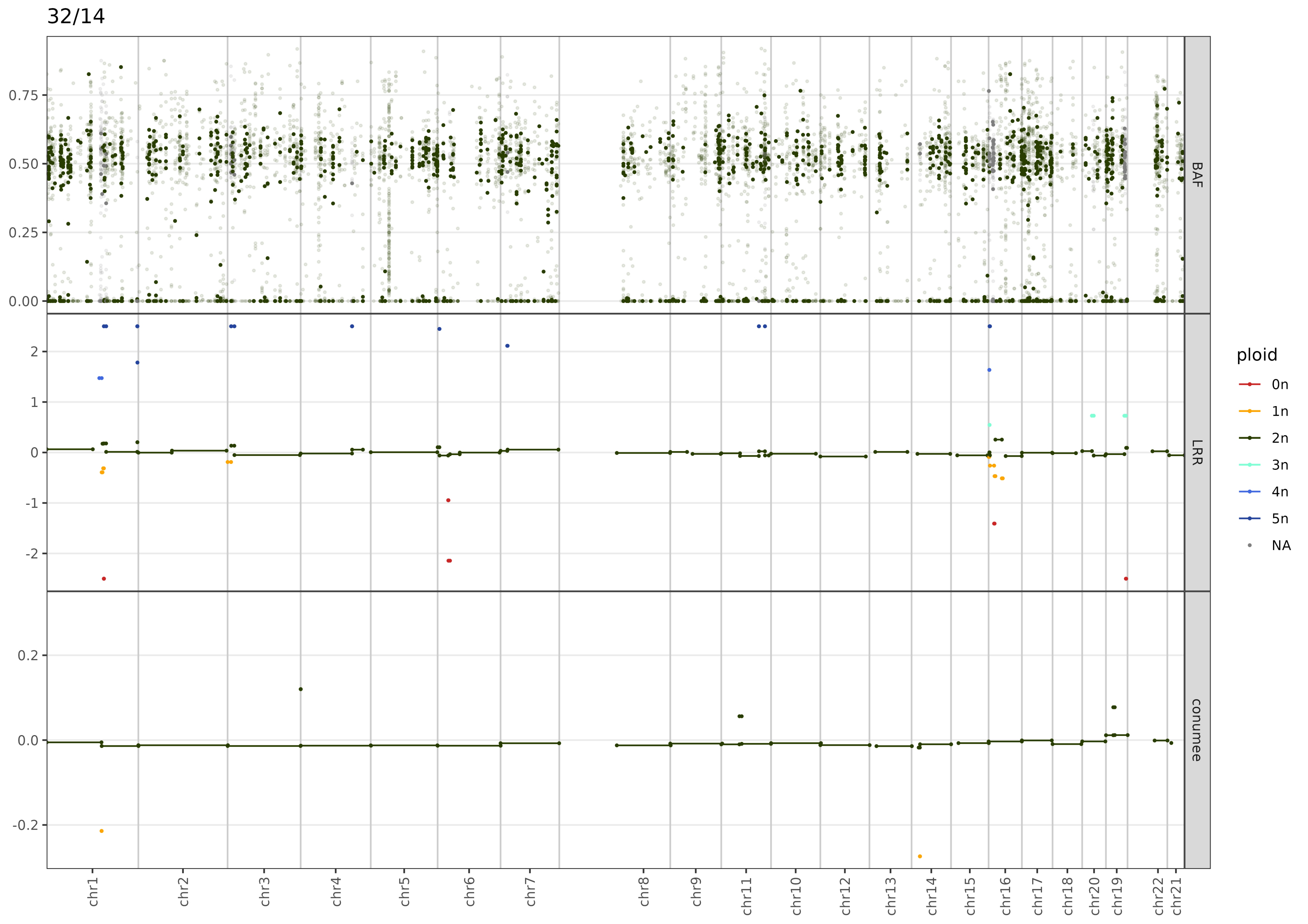

32/14
BAF
LRR
conumee
ploid
0n
1n
2n
3n
4n
5n
NA
0.75
0.50
0.25
0.00
2
1
0
-1
-2
0.2
0.0
-0.2
chr1
chr2
chr3
chr4
chr5
chr6
chr7
chr8
chr9
chr11
chr10
chr12
chr13
chr14
chr15
chr16
chr17
chr18
chr20
chr19
chr22
chr21

35/17

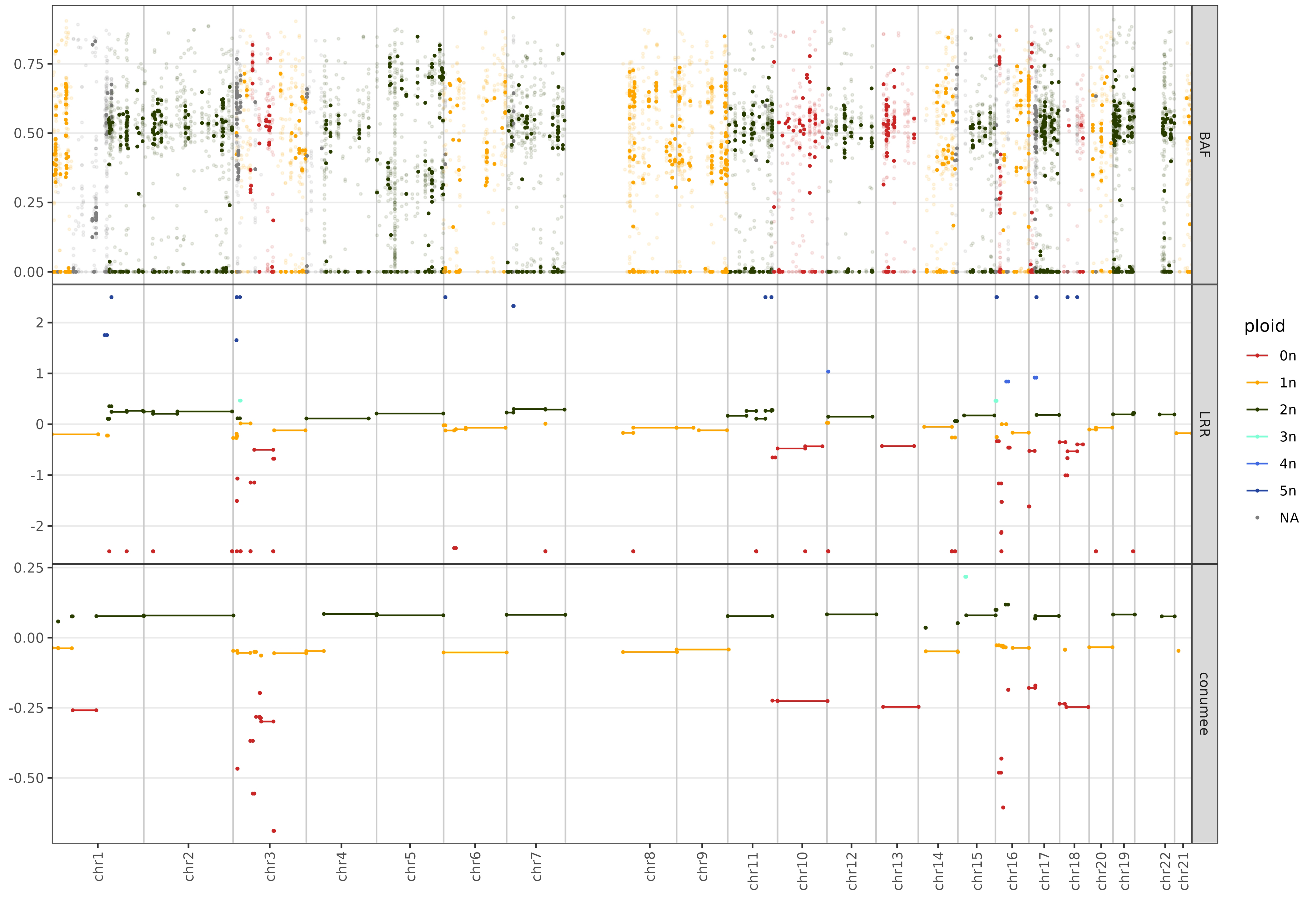

BAF
LRR
conumee
0.75
0.50
0.25
0.00
2
1
0
-1
-2
0.25
0.00
-0.25
-0.50
ploid
0n
1n
2n
3n
4n
5n
NA
chr1
chr2
chr3
chr4
chr5
chr6
chr7
chr8
chr9
chr11
chr10
chr12
chr13
chr14
chr15
chr16
chr17
chr18
chr20
chr19
chr22
chr21

37/19

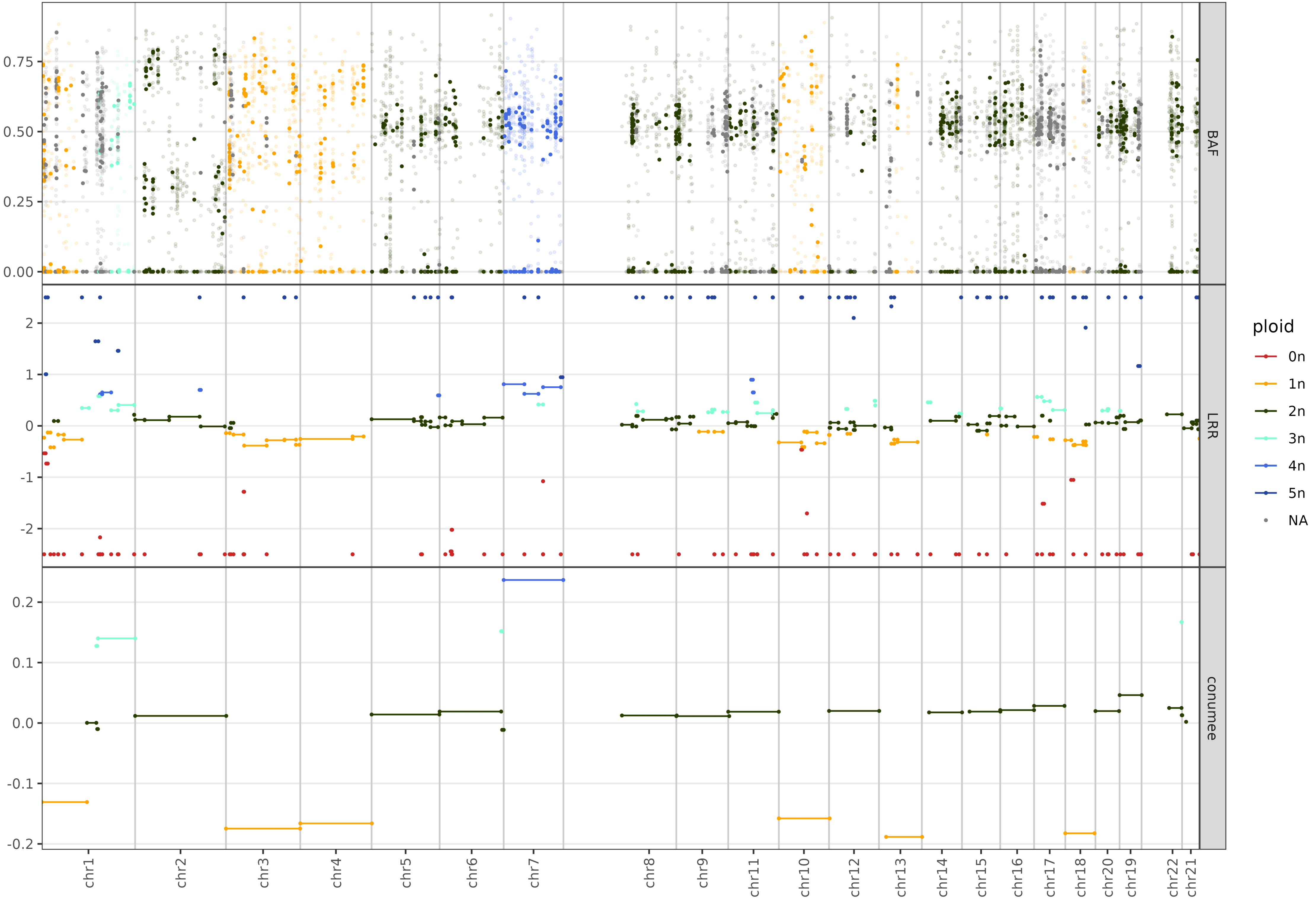

0.75
0.50
0.25
0.00
BAF
2
1
0
-1
-2
LRR
0.2
0.1
0.0
-0.1
-0.2
conumee
chr1
chr2
chr3
chr4
chr5
chr6
chr7
chr8
chr9
chr11
chr10
chr12
chr13
chr14
chr15
chr16
chr17
chr18
chr20
chr19
chr22
chr21
ploid
0n
1n
2n
3n
4n
5n
NA

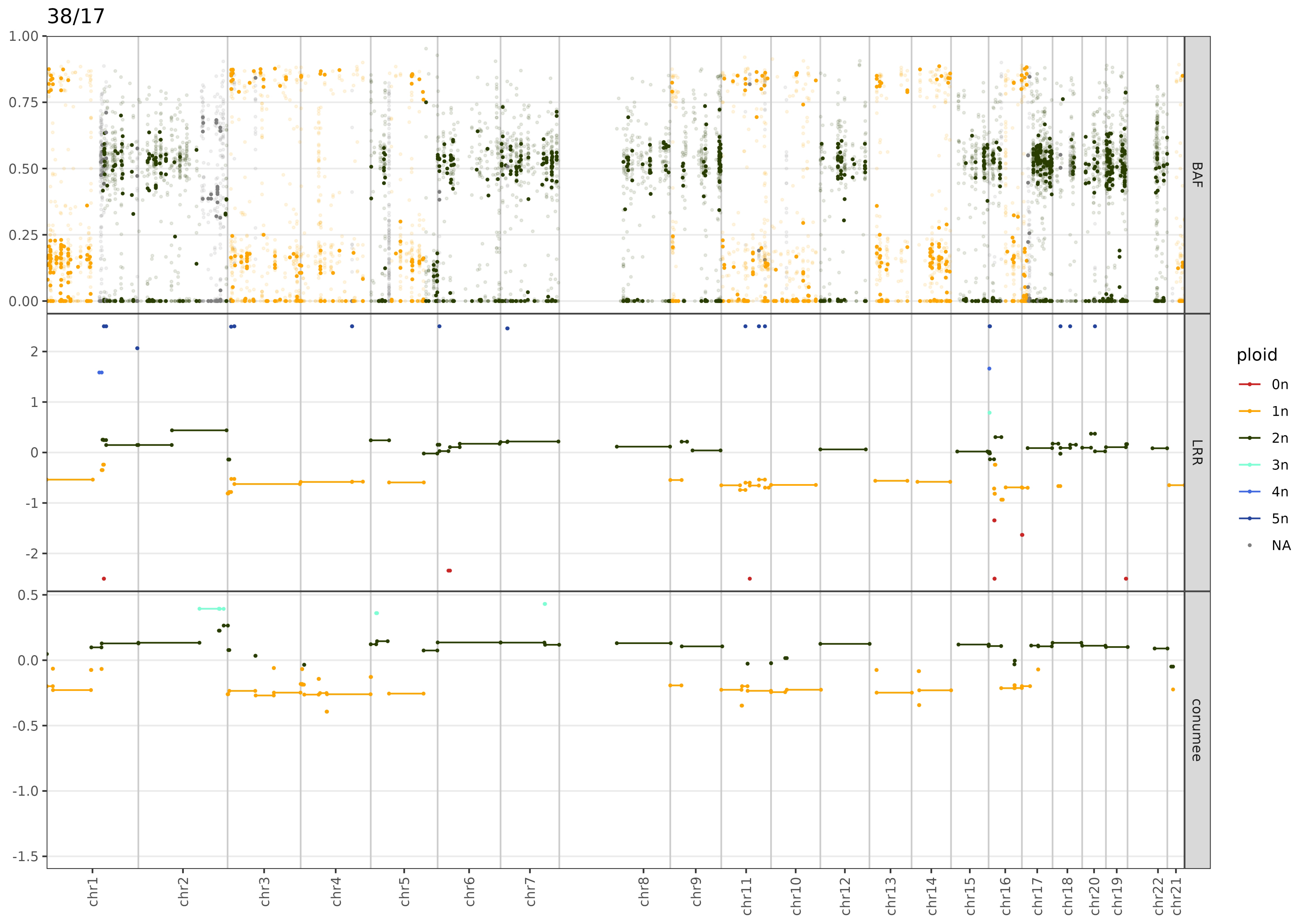

38/17
1.00
0.75
0.50
0.25
0.00
BAF
2
1
0
-1
-2
LRR
0.5
0.0
-0.5
-1.0
-1.5
conumee
chr1
chr2
chr3
chr4
chr5
chr6
chr7
chr8
chr9
chr11
chr10
chr12
chr13
chr14
chr15
chr16
chr17
chr18
chr20
chr19
chr22
chr21
ploid
0n
1n
2n
3n
4n
5n
NA

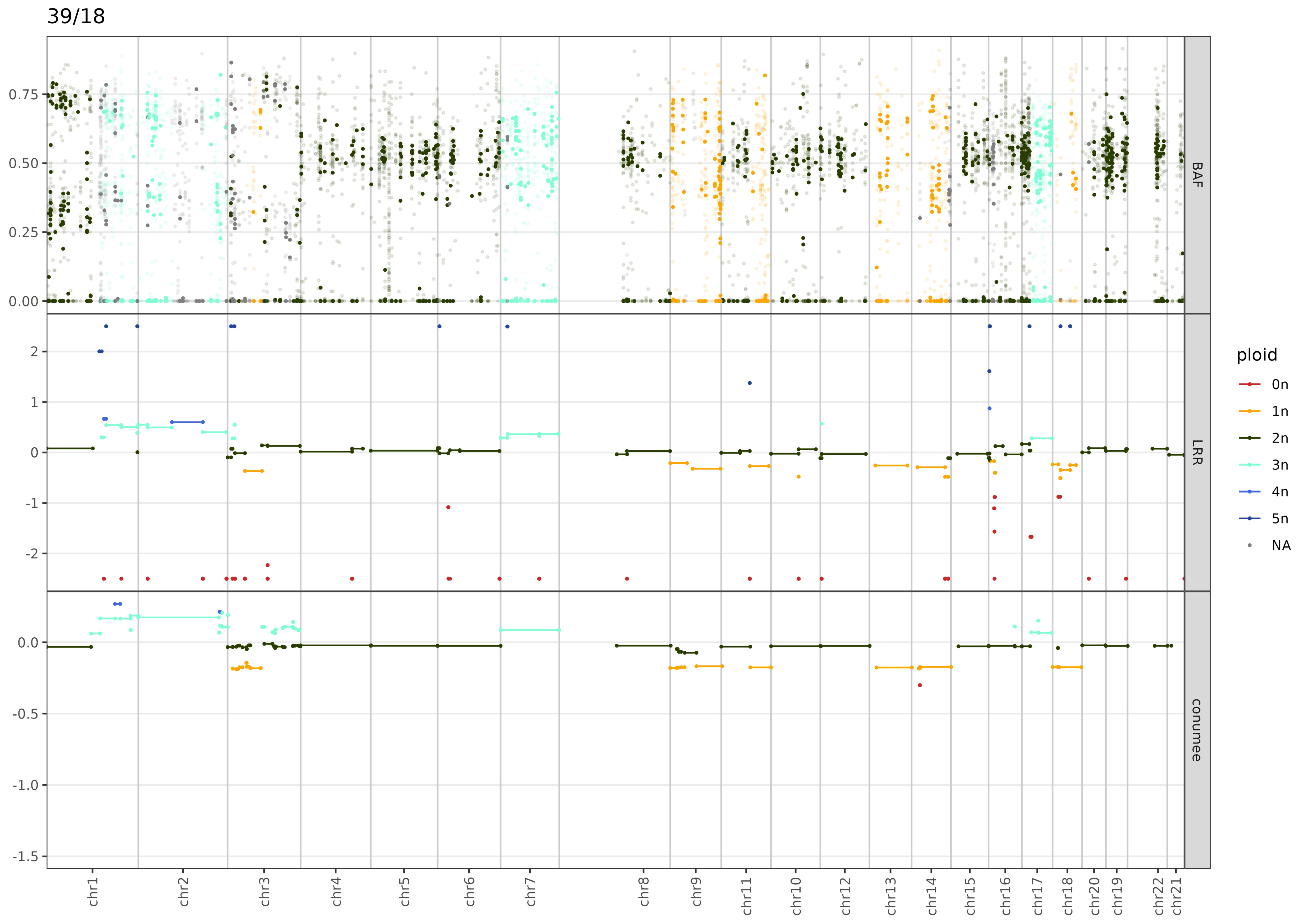
39/18
BAF
LRR
conumee
ploid
0n
1n
2n
3n
4n
5n
NA
0.75
0.50
0.25
0.00
2
1
0
-1
-2
0.0
-0.5
-1.0
-1.5
chr1
chr2
chr3
chr4
chr5
chr6
chr7
chr8
chr9
chr11
chr10
chr12
chr13
chr14
chr15
chr16
chr17
chr18
chr20
chr19
chr22
chr21

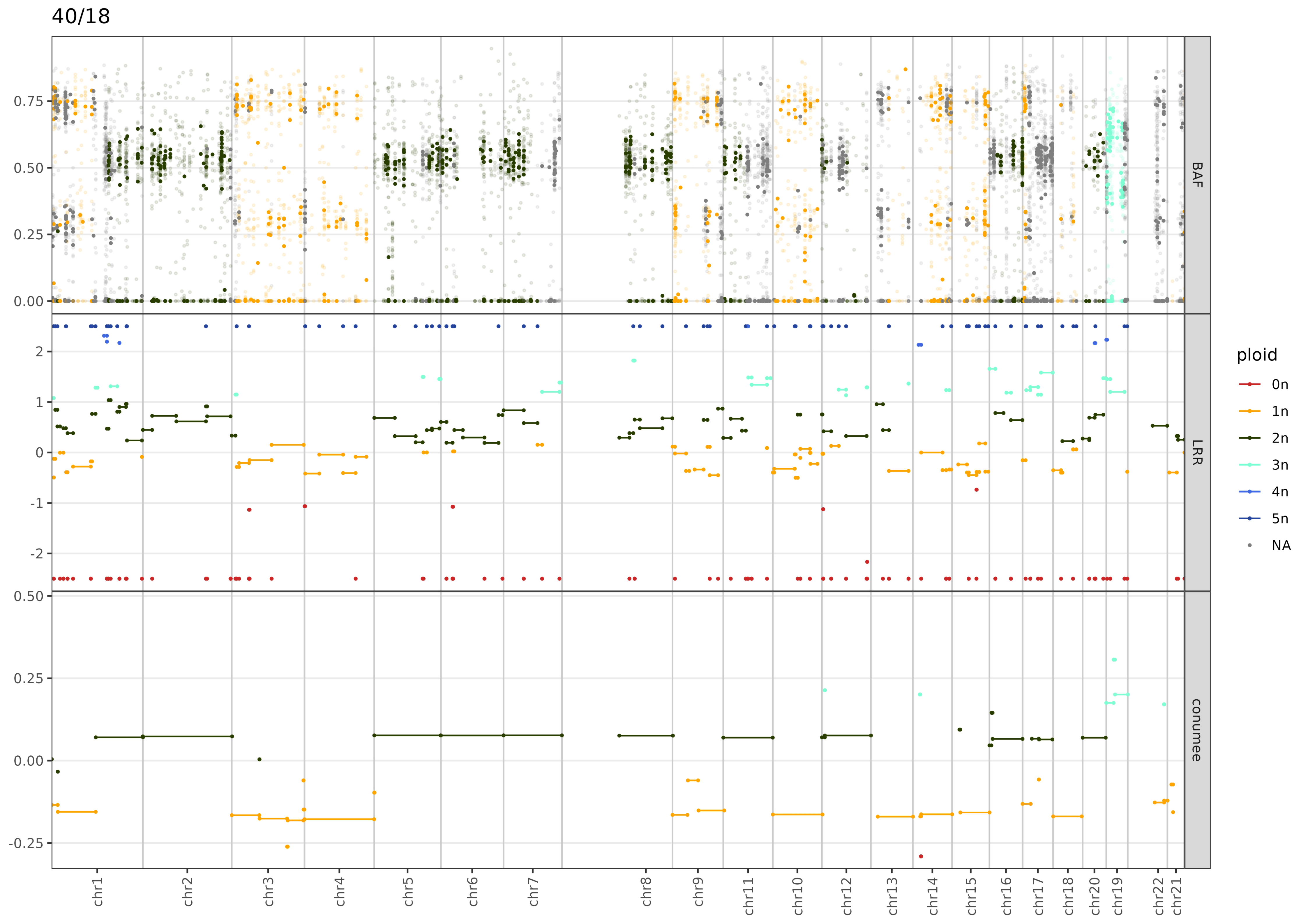
40/18
BAF
LRR
conumee
ploid
0n
1n
2n
3n
4n
5n
NA
0.75
0.50
0.25
0.00
2
1
0
-1
-2
0.50
0.25
0.00
-0.25
chr1
chr2
chr3
chr4
chr5
chr6
chr7
chr8
chr9
chr11
chr10
chr12
chr13
chr14
chr15
chr16
chr17
chr18
chr20
chr19
chr22
chr21

45/17

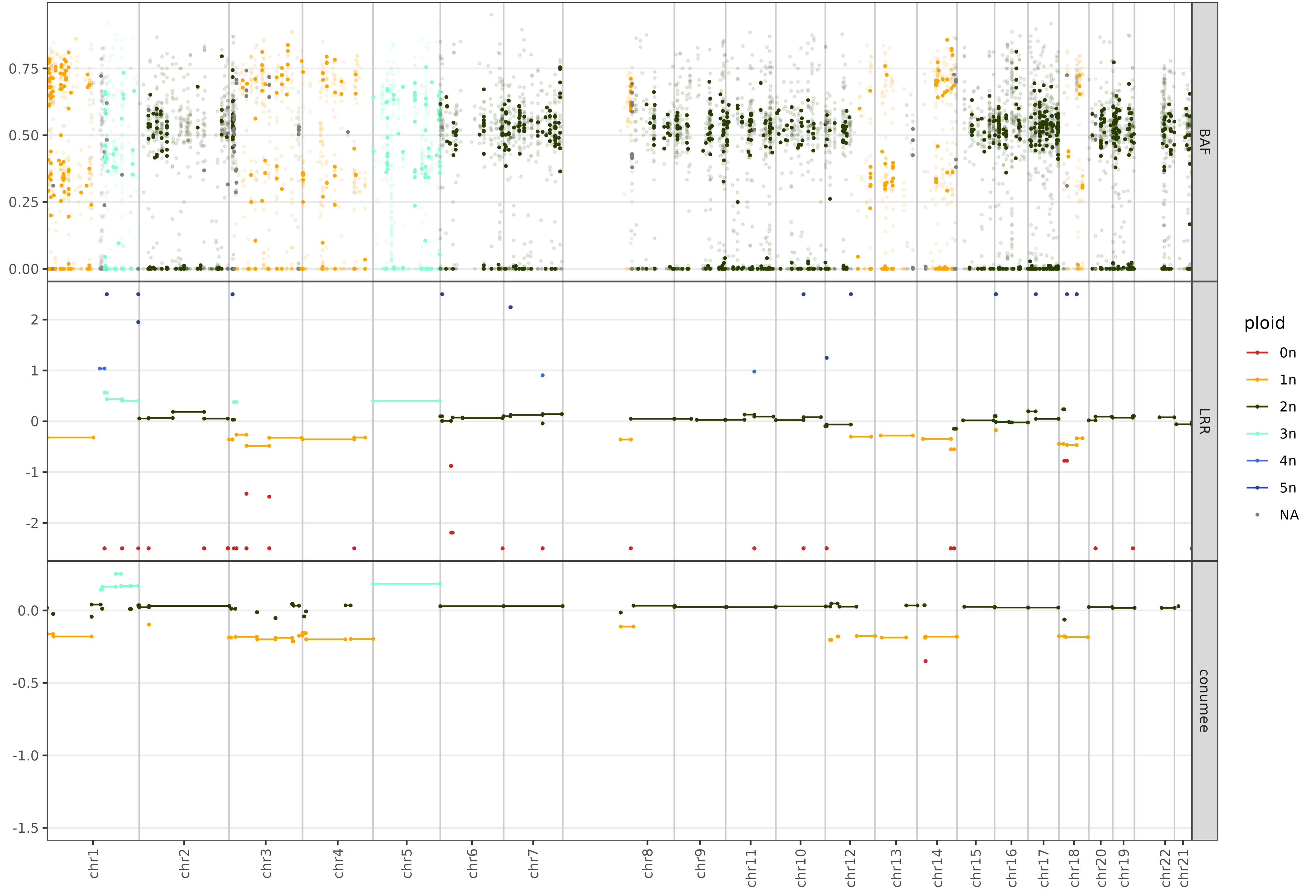

BAF
LRR
conumee
0.75
0.50
0.25
0.00
2
1
0
-1
-2
0.0
-0.5
-1.0
-1.5
chr1
chr2
chr3
chr4
chr5
chr6
chr7
chr8
chr9
chr11
chr10
chr12
chr13
chr14
chr15
chr16
chr17
chr18
chr20
chr19
chr22
chr21
ploid
0n
1n
2n
3n
4n
5n
NA

50/15

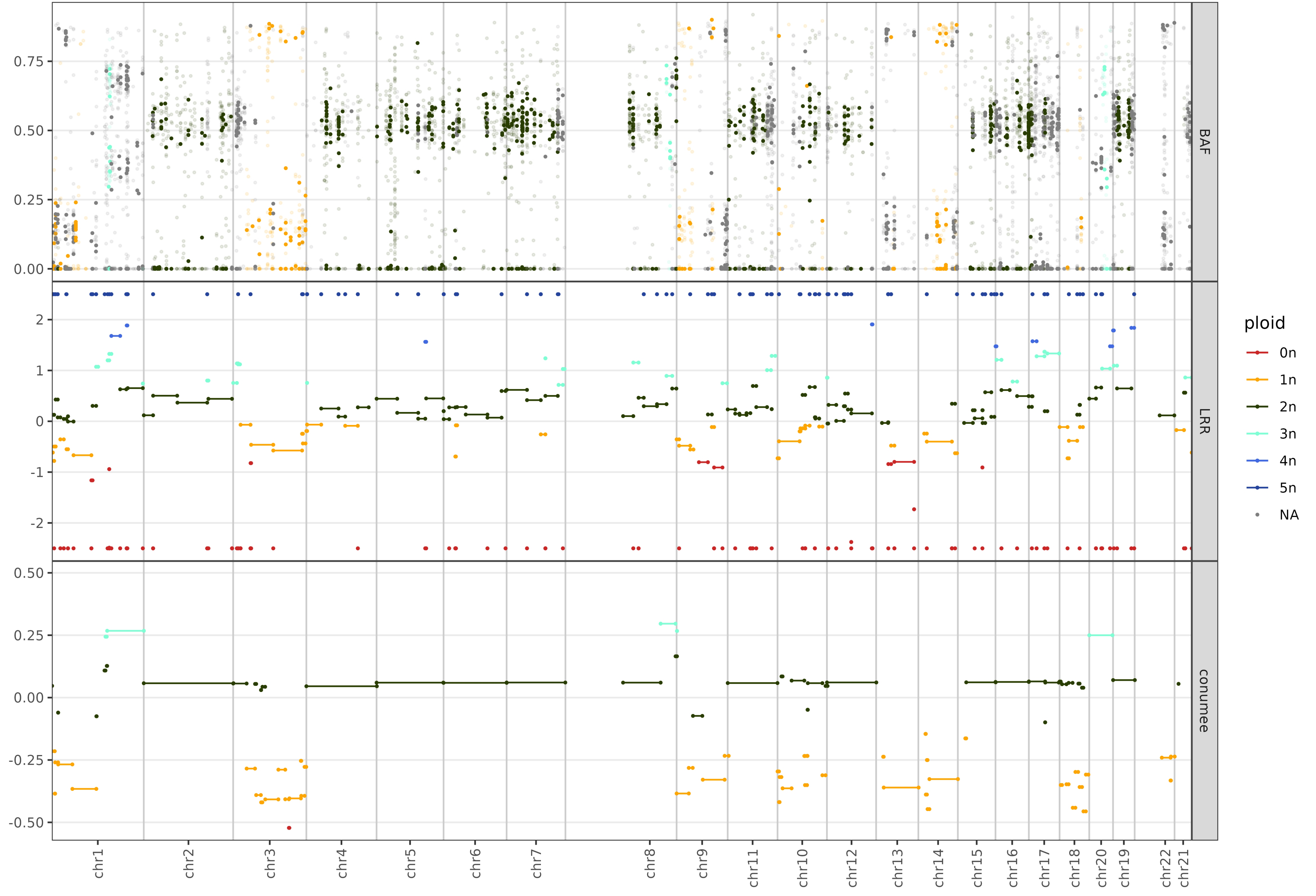
0.75
0.50
0.25
0.00
BAF
2
1
0
-1
-2
LRR
0.50
0.25
0.00
-0.25
-0.50
conumee
ploid
0n
1n
2n
3n
4n
5n
NA
chr1
chr2
chr3
chr4
chr5
chr6
chr7
chr8
chr9
chr11
chr10
chr12
chr13
chr14
chr15
chr16
chr17
chr18
chr20
chr19
chr22
chr21

50/19

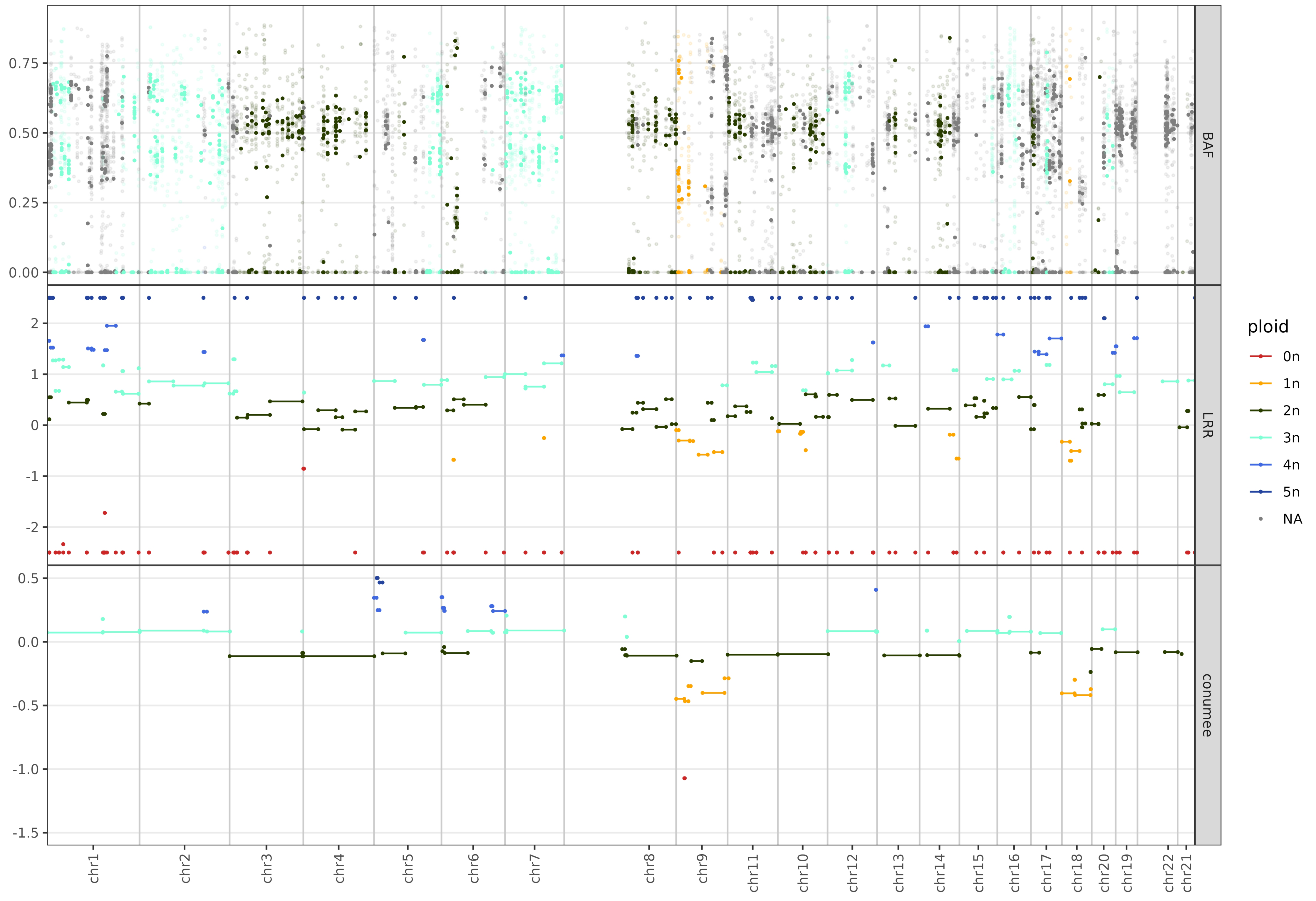

BAF
LRR
conumee
0.75
0.50
0.25
0.00
2
1
0
-1
-2
0.5
0.0
-0.5
-1.0
-1.5
chr1
chr2
chr3
chr4
chr5
chr6
chr7
chr8
chr9
chr11
chr10
chr12
chr13
chr14
chr15
chr16
chr17
chr18
chr20
chr19
chr22
chr21
ploid
0n
1n
2n
3n
4n
5n
NA

53/18

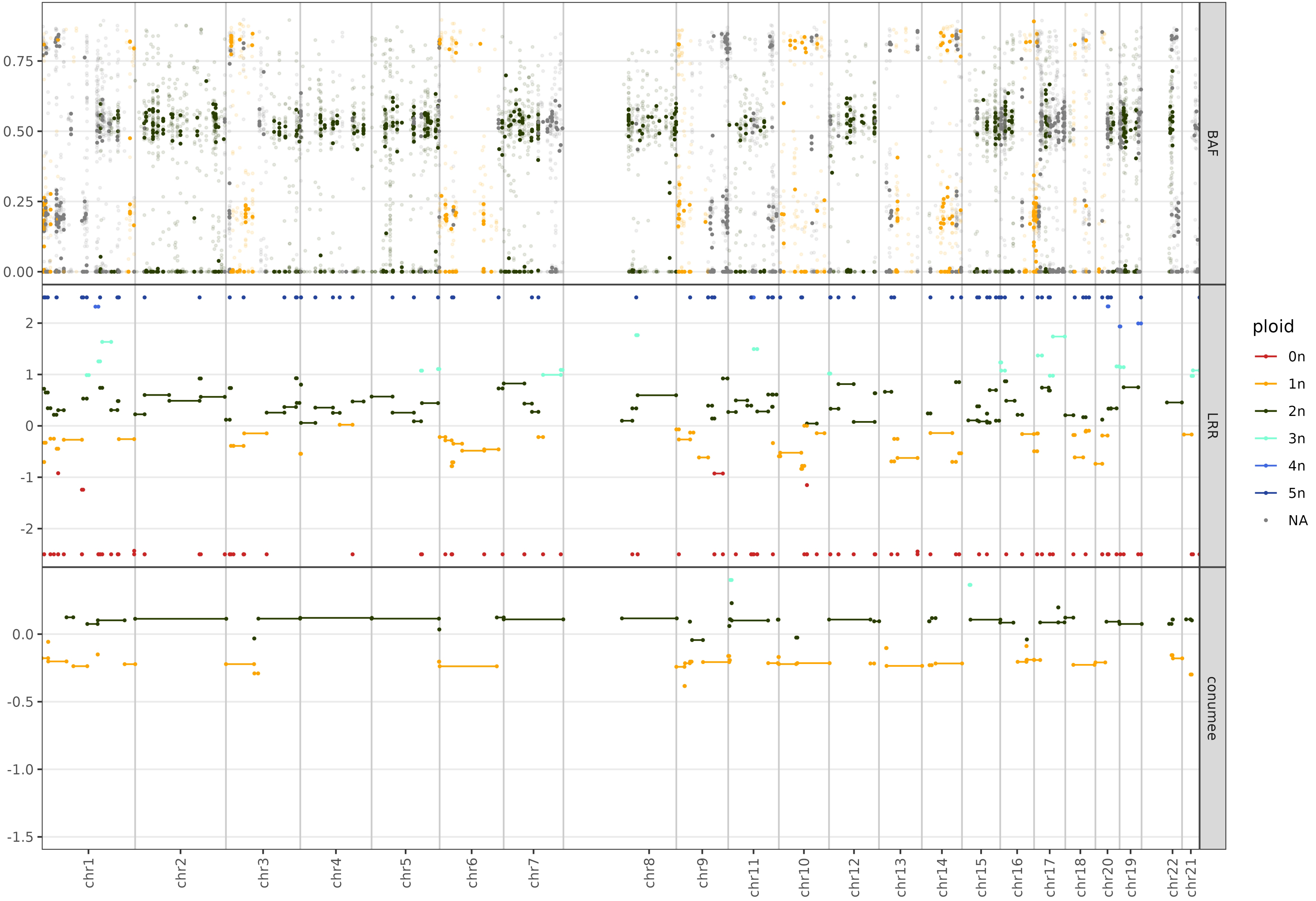

BAF
LRR
conumee
0.75
0.50
0.25
0.00
2
1
0
-1
-2
0.0
-0.5
-1.0
-1.5
ploid
0n
1n
2n
3n
4n
5n
NA
chr1
chr2
chr3
chr4
chr5
chr6
chr7
chr8
chr9
chr11
chr10
chr12
chr13
chr14
chr15
chr16
chr17
chr18
chr20
chr19
chr22
chr21

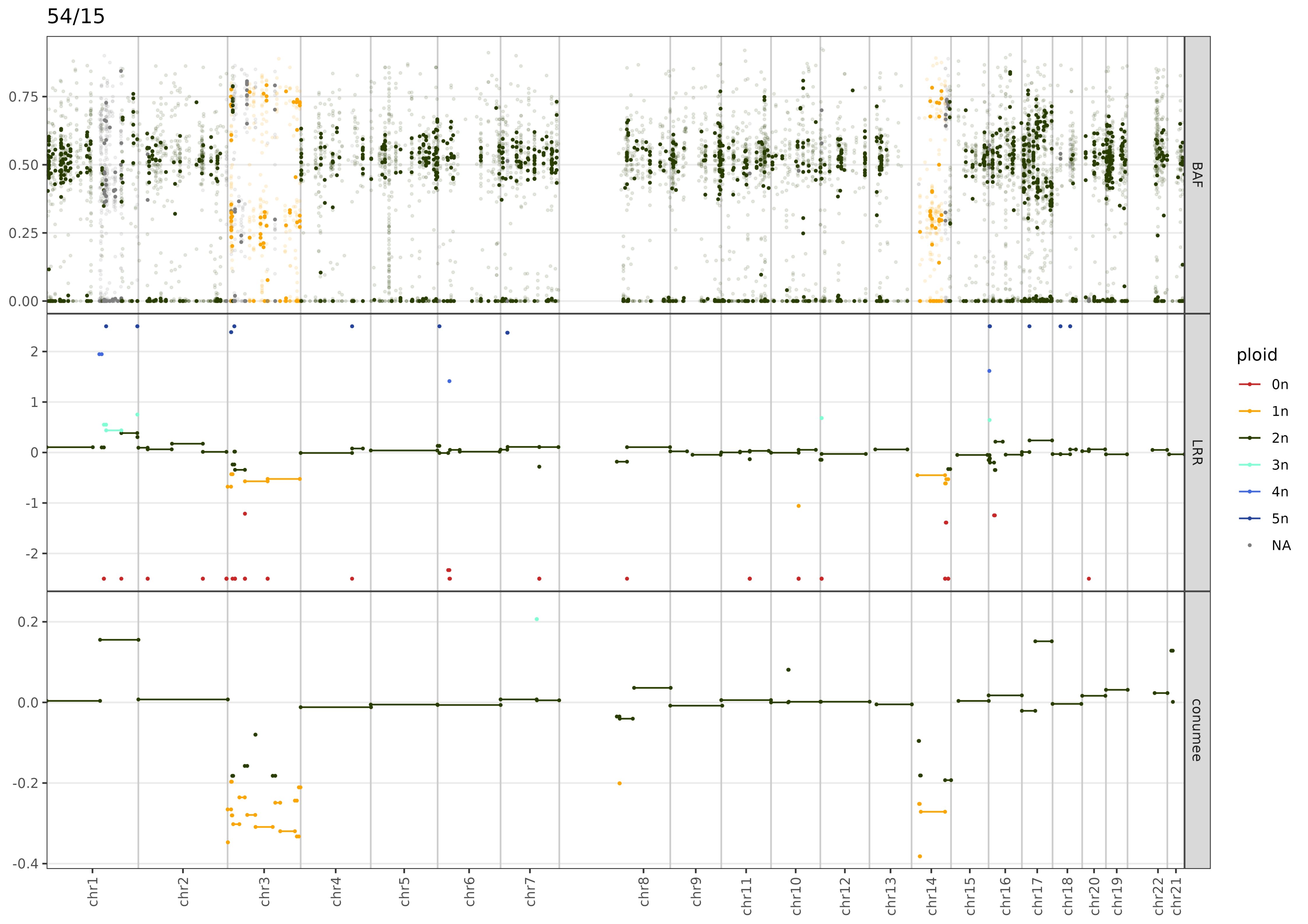
54/15
BAF
LRR
conumee
ploid
0n
1n
2n
3n
4n
5n
NA
0.75
0.50
0.25
0.00
2
1
0
-1
-2
0.2
0.0
-0.2
-0.4
chr1
chr2
chr3
chr4
chr5
chr6
chr7
chr8
chr9
chr11
chr10
chr12
chr13
chr14
chr15
chr16
chr17
chr18
chr20
chr19
chr22
chr21

55/19

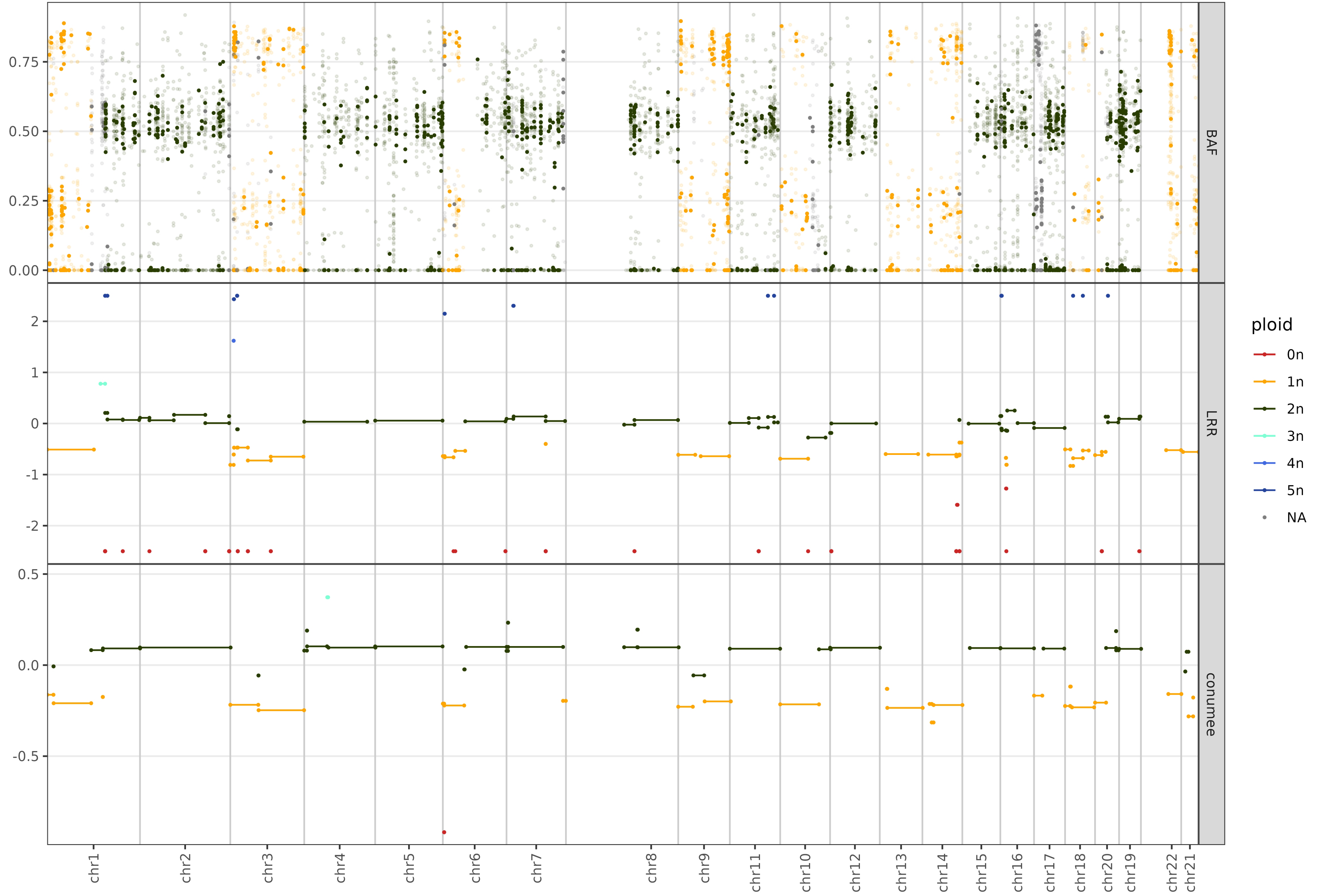

BAF
LRR
conumee
0.75
0.50
0.25
0.00
2
1
0
-1
-2
0.5
0.0
-0.5
chr1
chr2
chr3
chr4
chr5
chr6
chr7
chr8
chr9
chr11
chr10
chr12
chr13
chr14
chr15
chr16
chr17
chr18
chr20
chr19
chr22
chr21
ploid
0n
1n
2n
3n
4n
5n
NA

62/15

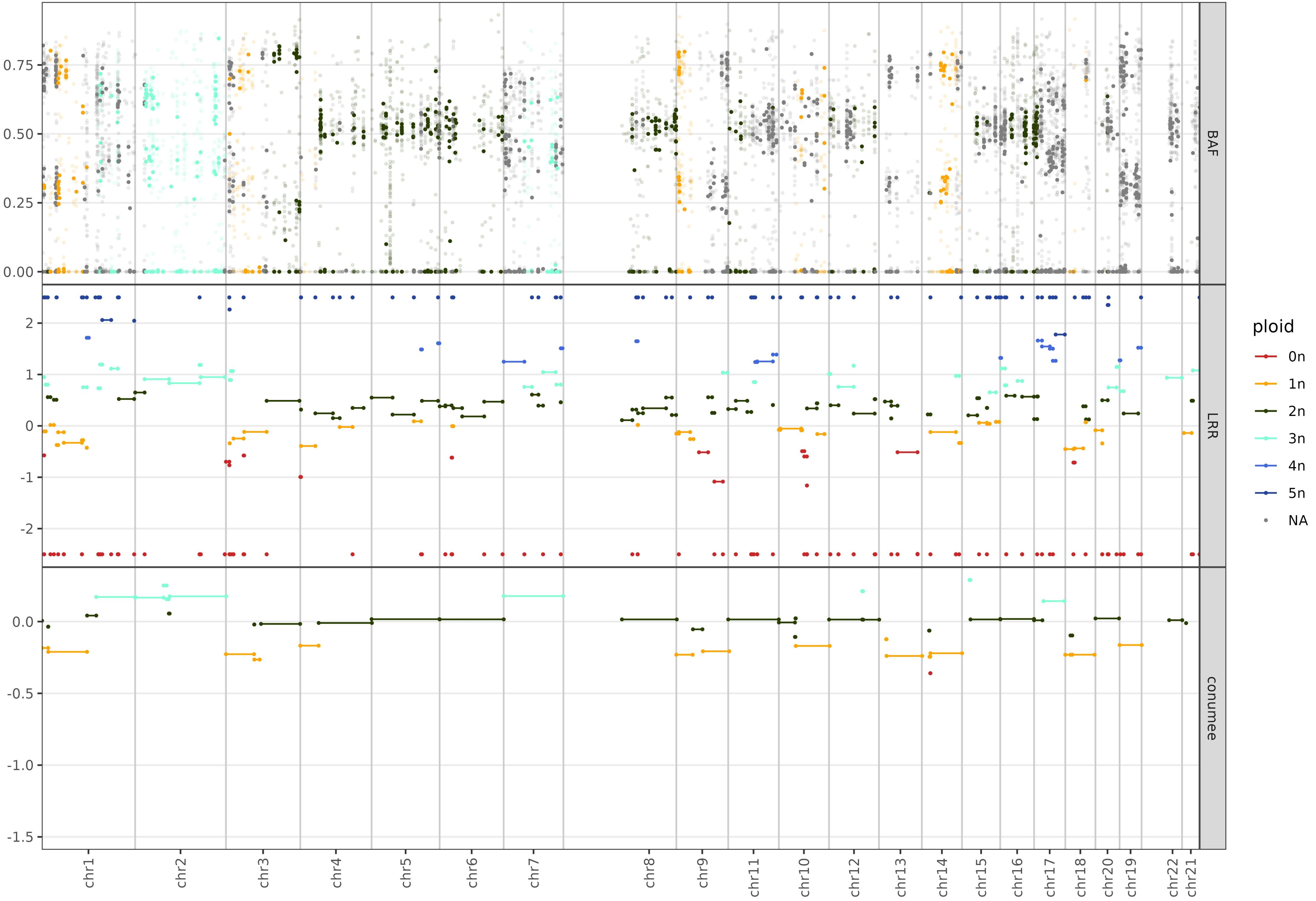

BAF
LRR
conumee
0.75
0.50
0.25
0.00
2
1
0
-1
-2
0.0
-0.5
-1.0
-1.5
chr1
chr2
chr3
chr4
chr5
chr6
chr7
chr8
chr9
chr11
chr10
chr12
chr13
chr14
chr15
chr16
chr17
chr18
chr20
chr19
chr22
chr21
ploid
0n
1n
2n
3n
4n
5n
NA

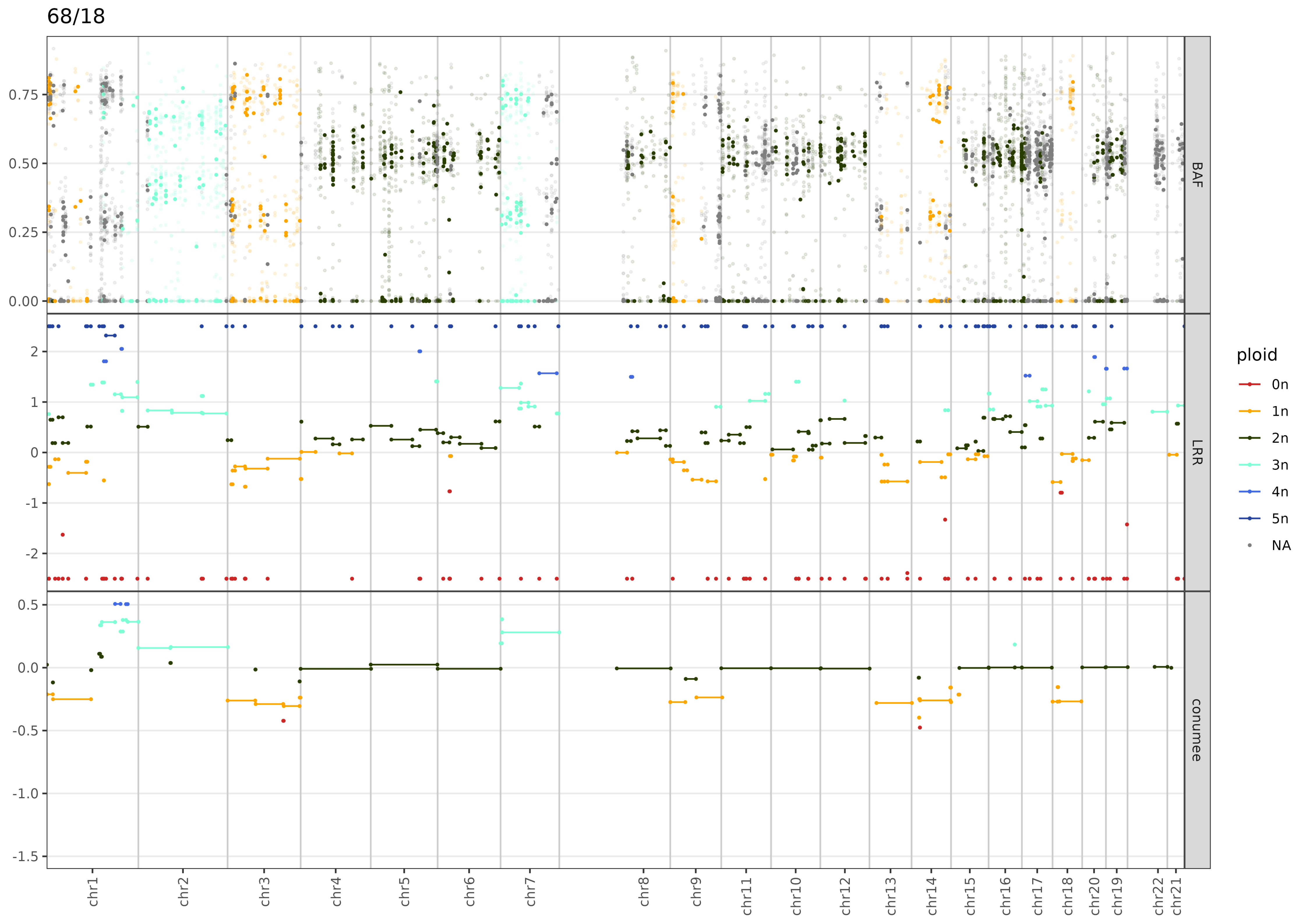
68/18
BAF
LRR
conumee
ploid
0n
1n
2n
3n
4n
5n
NA
chr1
chr2
chr3
chr4
chr5
chr6
chr7
chr8
chr9
chr11
chr10
chr12
chr13
chr14
chr15
chr16
chr17
chr18
chr20
chr19
chr22
chr21

74/19

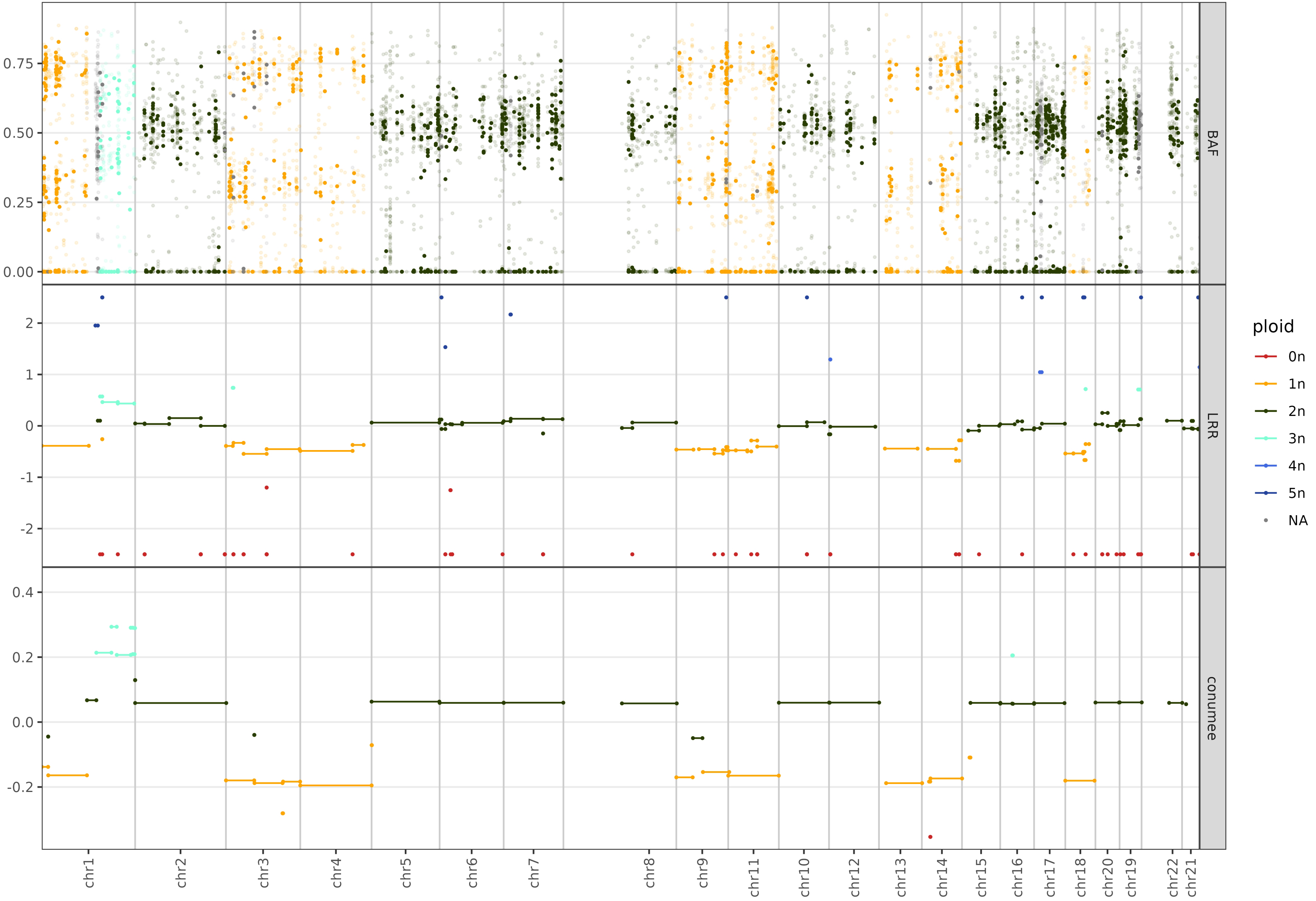

BAF
LRR
conumee
0.75
0.50
0.25
0.00
2
1
0
-1
-2
0.4
0.2
0.0
-0.2
chr1
chr2
chr3
chr4
chr5
chr6
chr7
chr8
chr9
chr11
chr10
chr12
chr13
chr14
chr15
chr16
chr17
chr18
chr20
chr19
chr22
chr21
ploid
0n
1n
2n
3n
4n
5n
NA

81/15

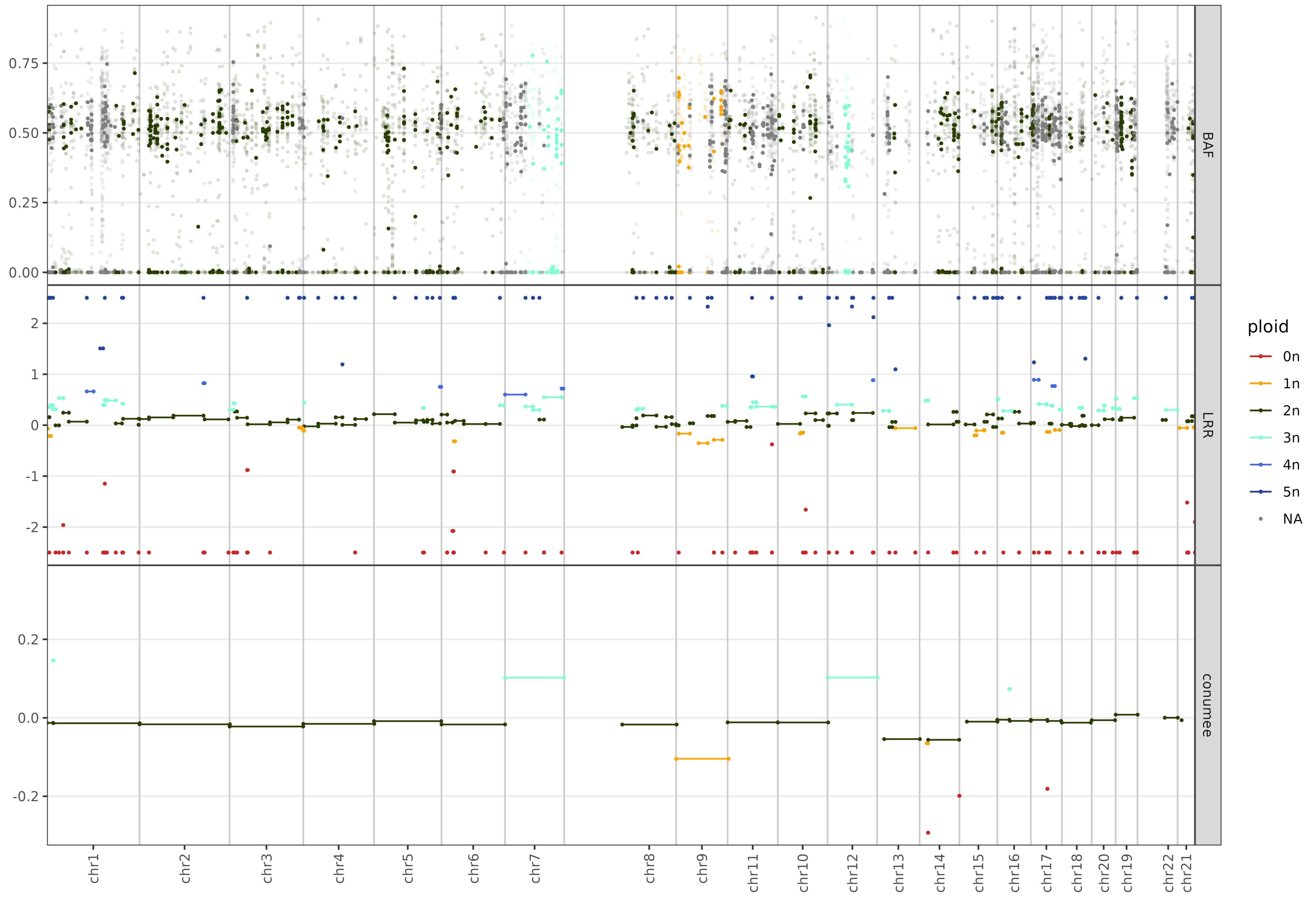
BAF
LRR
conumee
0.75
0.50
0.25
0.00
2
1
0
-1
-2
0.2
0.0
-0.2
chr1
chr2
chr3
chr4
chr5
chr6
chr7
chr8
chr9
chr11
chr10
chr12
chr13
chr14
chr15
chr16
chr17
chr18
chr20
chr19
chr22
chr21
ploid
0n
1n
2n
3n
4n
5n
NA

92/15

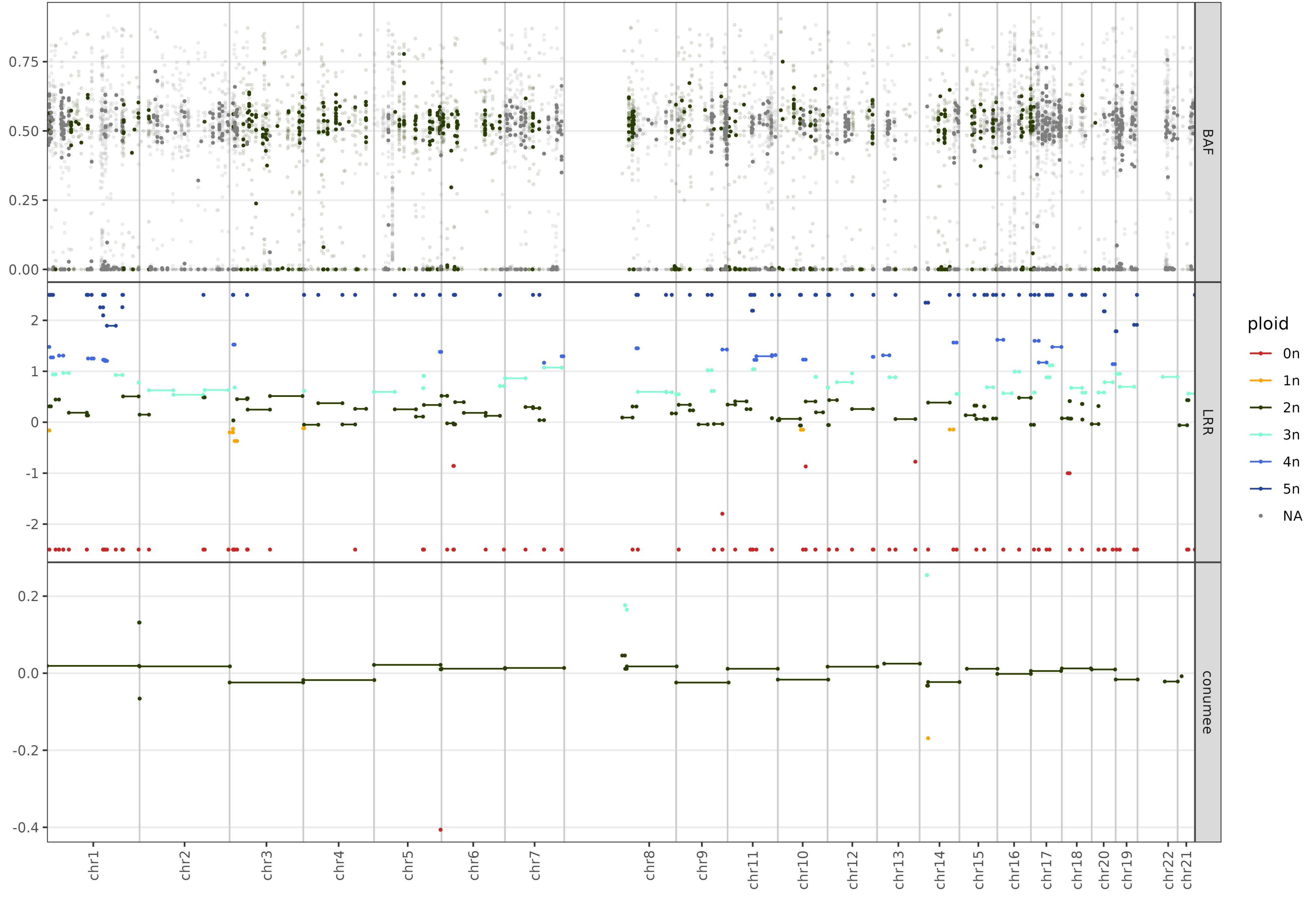

BAF
LRR
conumee
0.75
0.50
0.25
0.00
2
1
0
-1
-2
0.2
0.0
-0.2
-0.4
ploid
0n
1n
2n
3n
4n
5n
NA
chr1
chr2
chr3
chr4
chr5
chr6
chr7
chr8
chr9
chr11
chr10
chr12
chr13
chr14
chr15
chr16
chr17
chr18
chr20
chr19
chr22
chr21

4/19

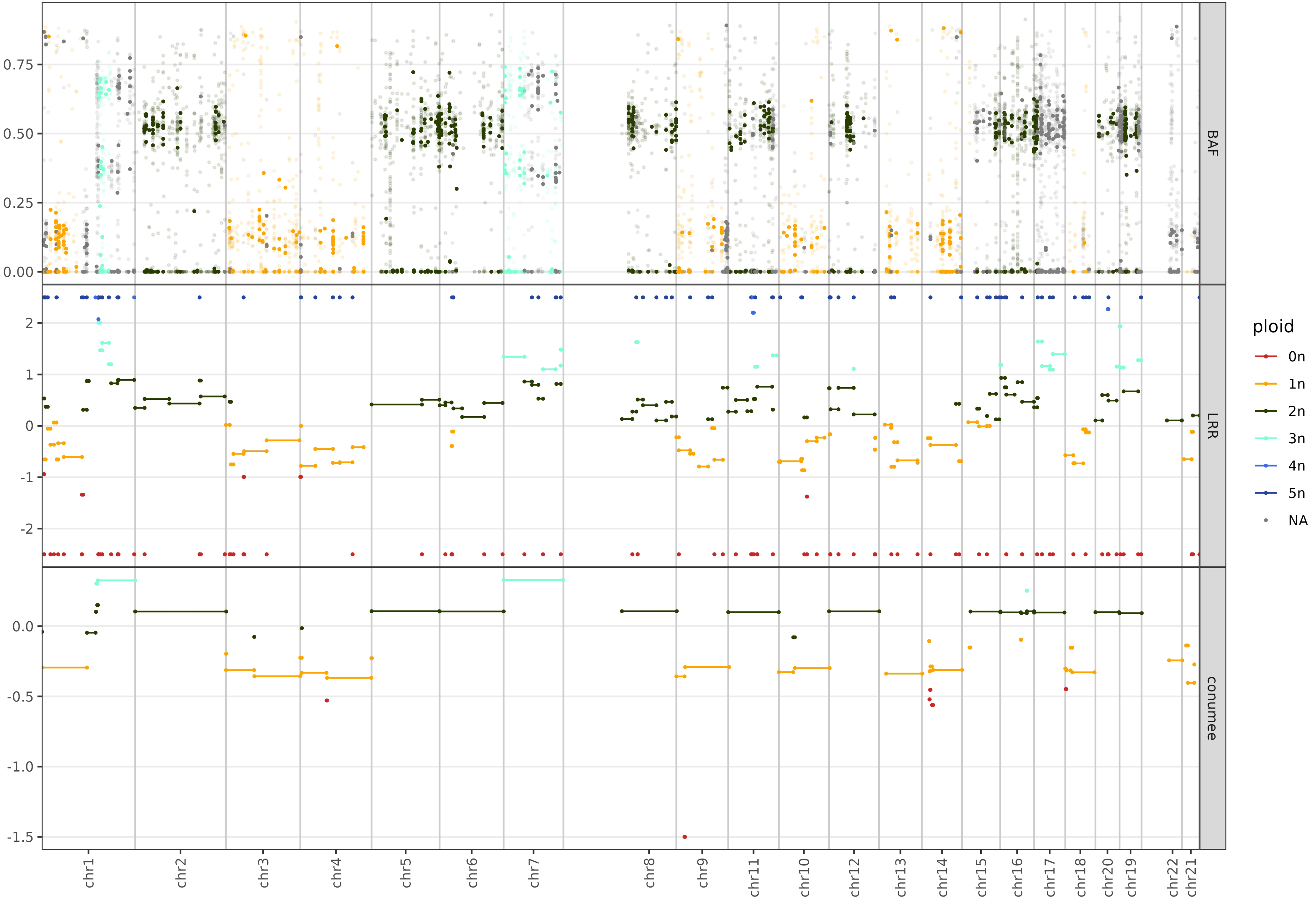

BAF
LRR
conumee
ploid
0n
1n
2n
3n
4n
5n
NA
0.75
0.50
0.25
0.00
2
1
0
-1
-2
0.0
-0.5
-1.0
-1.5
chr1
chr2
chr3
chr4
chr5
chr6
chr7
chr8
chr9
chr11
chr10
chr12
chr13
chr14
chr15
chr16
chr17
chr18
chr20
chr19
chr22
chr21

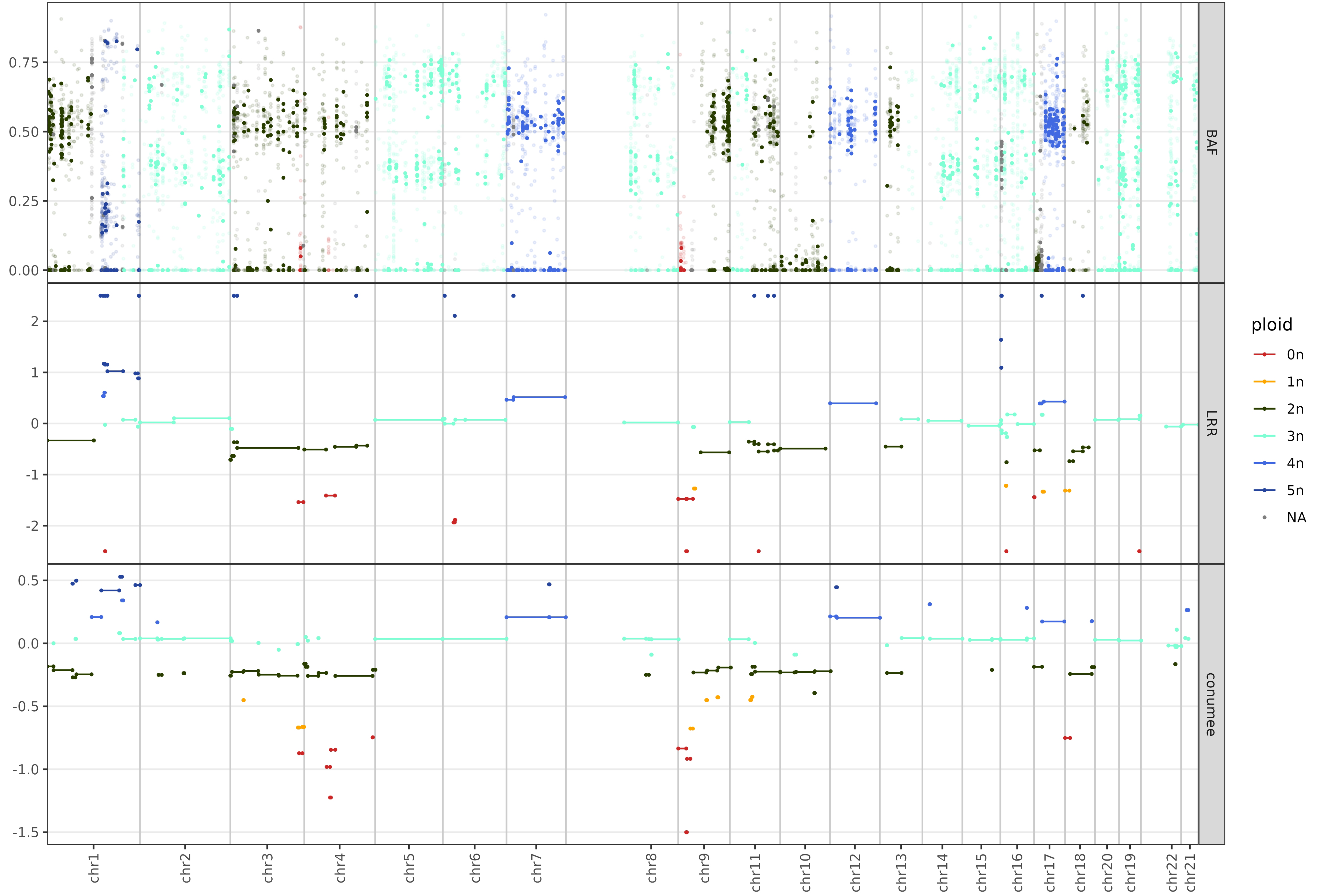
BAF
LRR
conumee
ploid
0n
1n
2n
3n
4n
5n
NA
0.75
0.50
0.25
0.00
2
1
0
-1
-2
0.5
0.0
-0.5
-1.0
-1.5
chr1
chr2
chr3
chr4
chr5
chr6
chr7
chr8
chr9
chr11
chr10
chr12
chr13
chr14
chr15
chr16
chr17
chr18
chr20
chr19
chr22
chr21

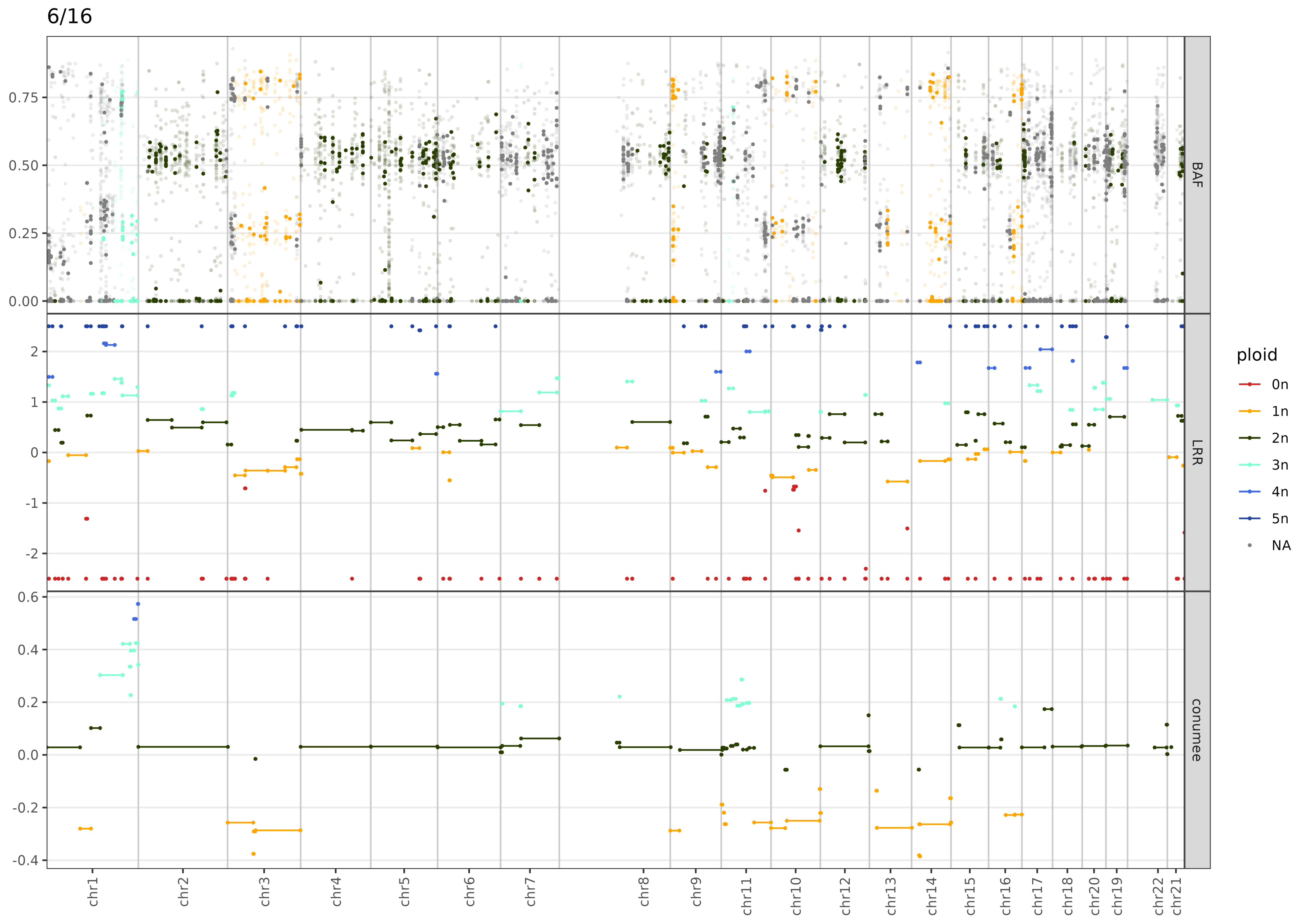

6/16
BAF
LRR
conumee
0.75
0.50
0.25
0.00
2
1
0
-1
-2
0.6
0.4
0.2
0.0
-0.2
-0.4
chr1
chr2
chr3
chr4
chr5
chr6
chr7
chr8
chr9
chr11
chr10
chr12
chr13
chr14
chr15
chr16
chr17
chr18
chr20
chr19
chr22
chr21
ploid
0n
1n
2n
3n
4n
5n
NA

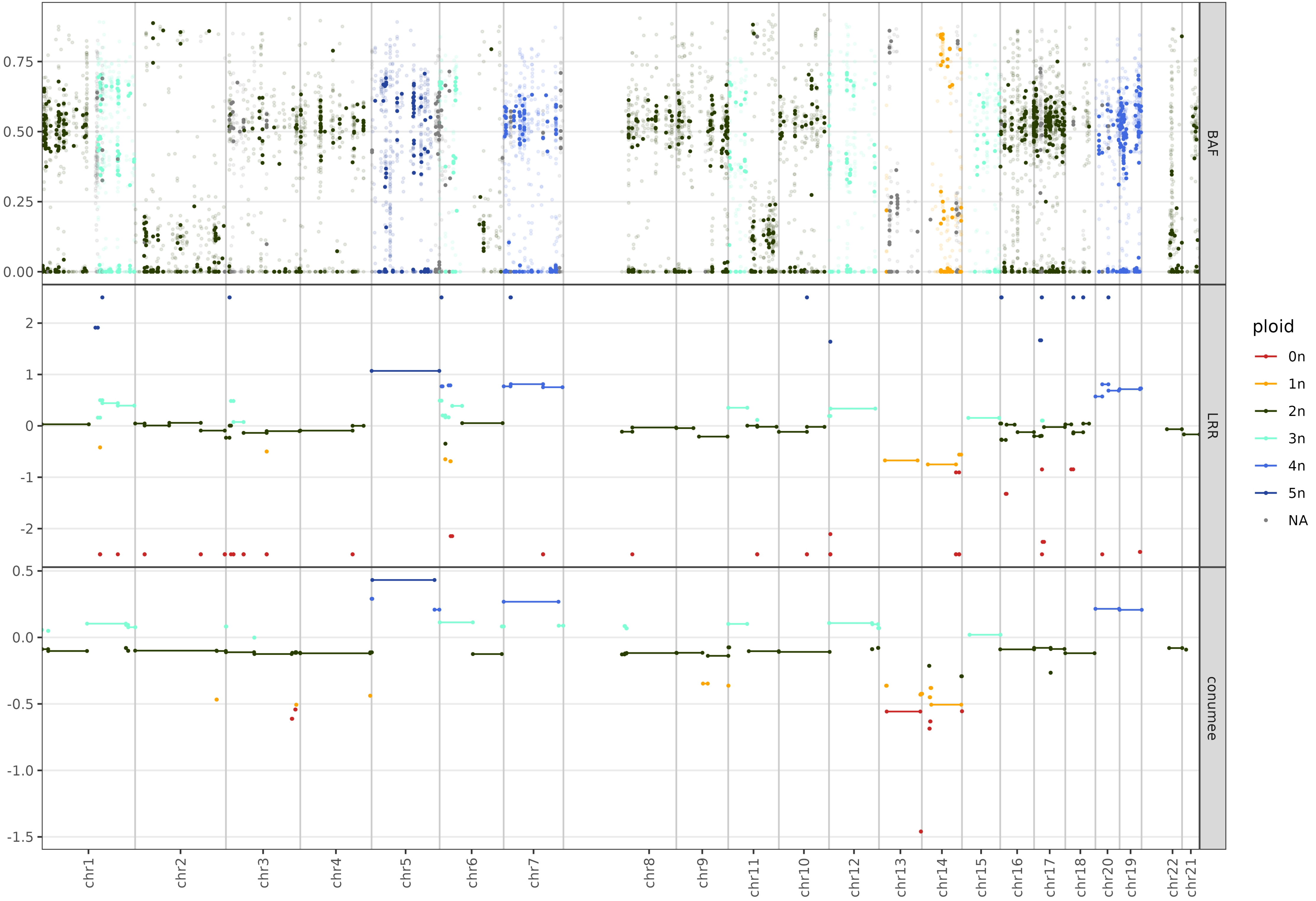

BAF
LRR
conumee
0.75
0.50
0.25
0.00
2
1
0
-1
-2
0.5
0.0
-0.5
-1.0
-1.5
chr1
chr2
chr3
chr4
chr5
chr6
chr7
chr8
chr9
chr11
chr10
chr12
chr13
chr14
chr15
chr16
chr17
chr18
chr20
chr19
chr22
chr21
ploid
0n
1n
2n
3n
4n
5n
NA

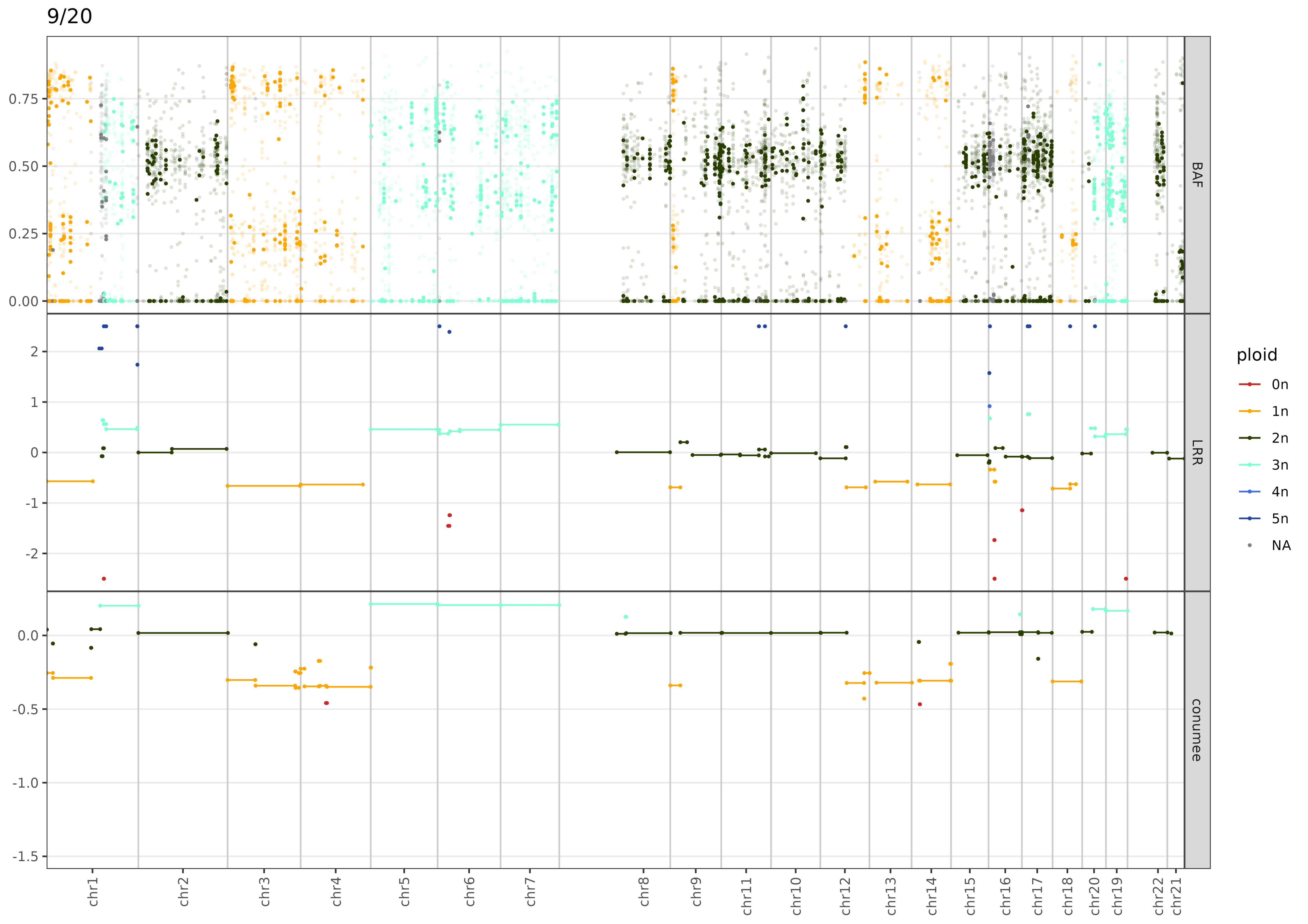

9/20
BAF
LRR
conumee
ploid
0n
1n
2n
3n
4n
5n
NA
0.00
0.25
0.50
0.75
-2
-1
0
1
2
-1.5
-1.0
-0.5
0.0
chr1 chr2 chr3 chr4 chr5 chr6 chr7 chr8 chr9 chr11 chr10 chr12 chr13 chr14 chr15 chr16 chr17 chr18 chr20 chr19 chr22 chr21

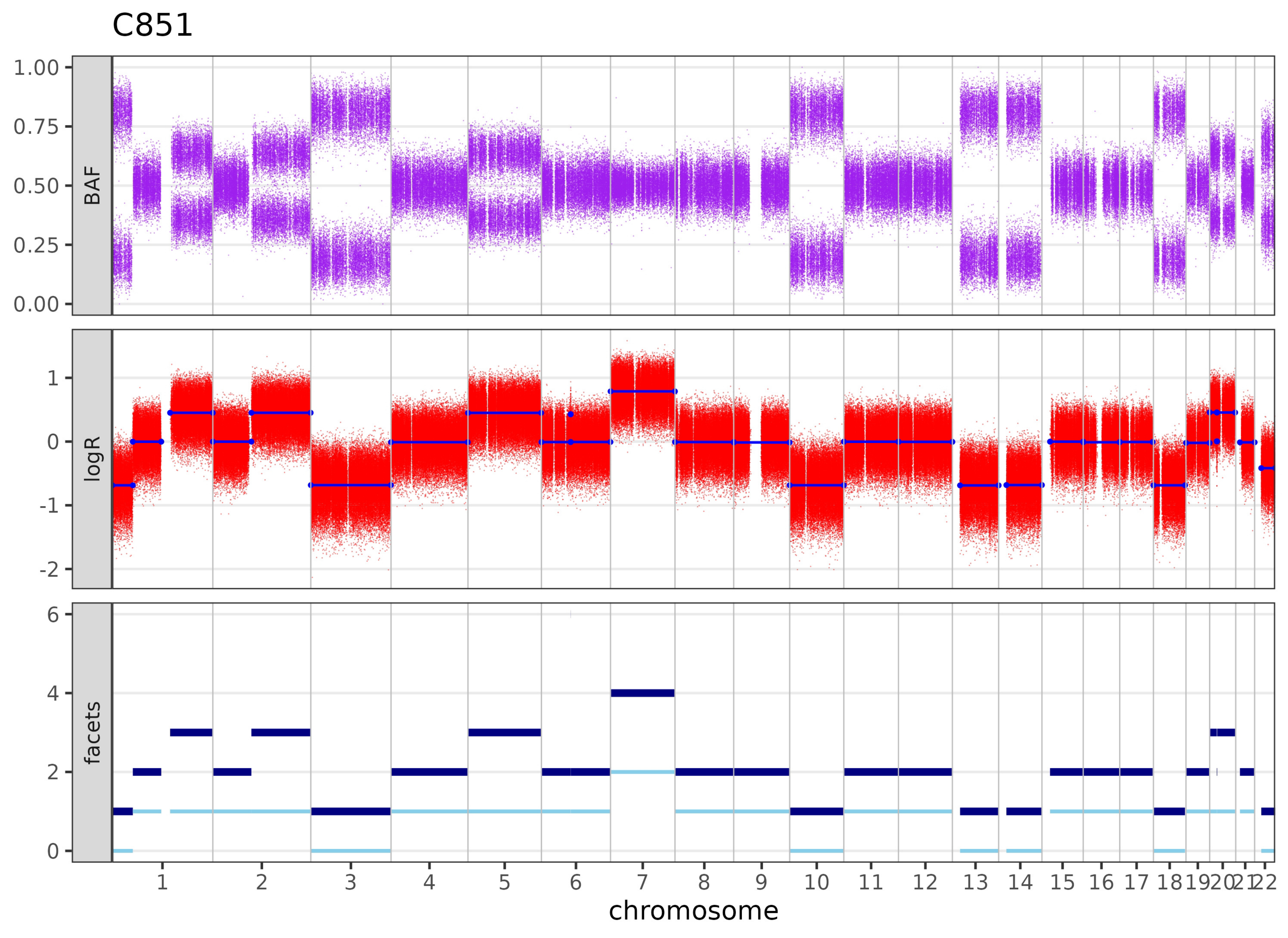
C851
BAF
1.00
0.75
0.50
0.25
0.00
logR
1
0
-1
-2
facets
6
4
2
0
1 2 3 4 5 6 7 8 9 10 11 12 13 14 15 16 17 18 19 20 21 22
chromosome

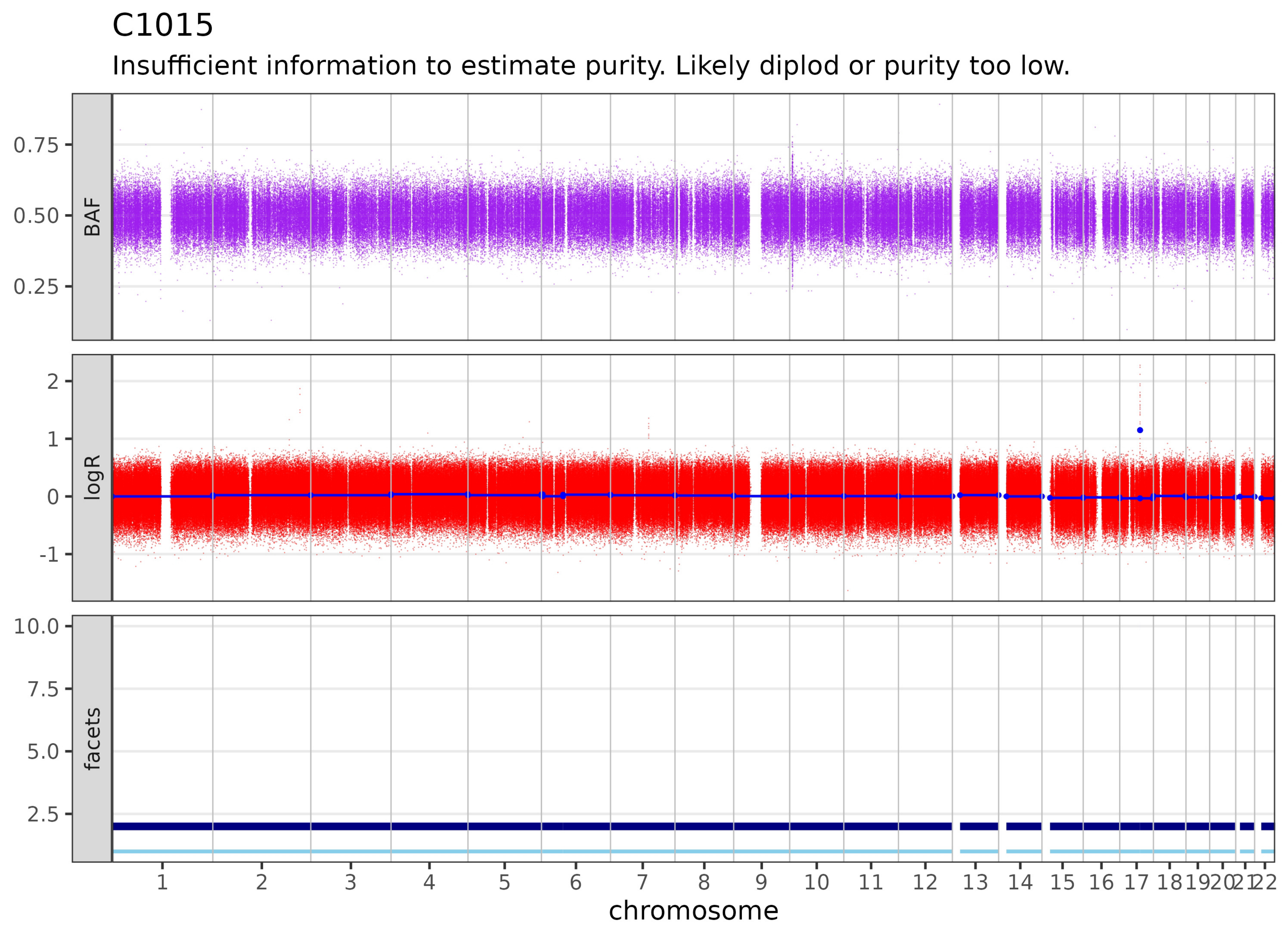
C1015
Insufficient information to estimate purity. Likely diplod or purity too low.
BAF
0.75
0.50
0.25
logR
2
1
0
-1
facets
10.0
7.5
5.0
2.5
1
2
3
4
5
6
7
8
9
10
11
12
13
14
15
16
17
18
19
20
21
22
chromosome

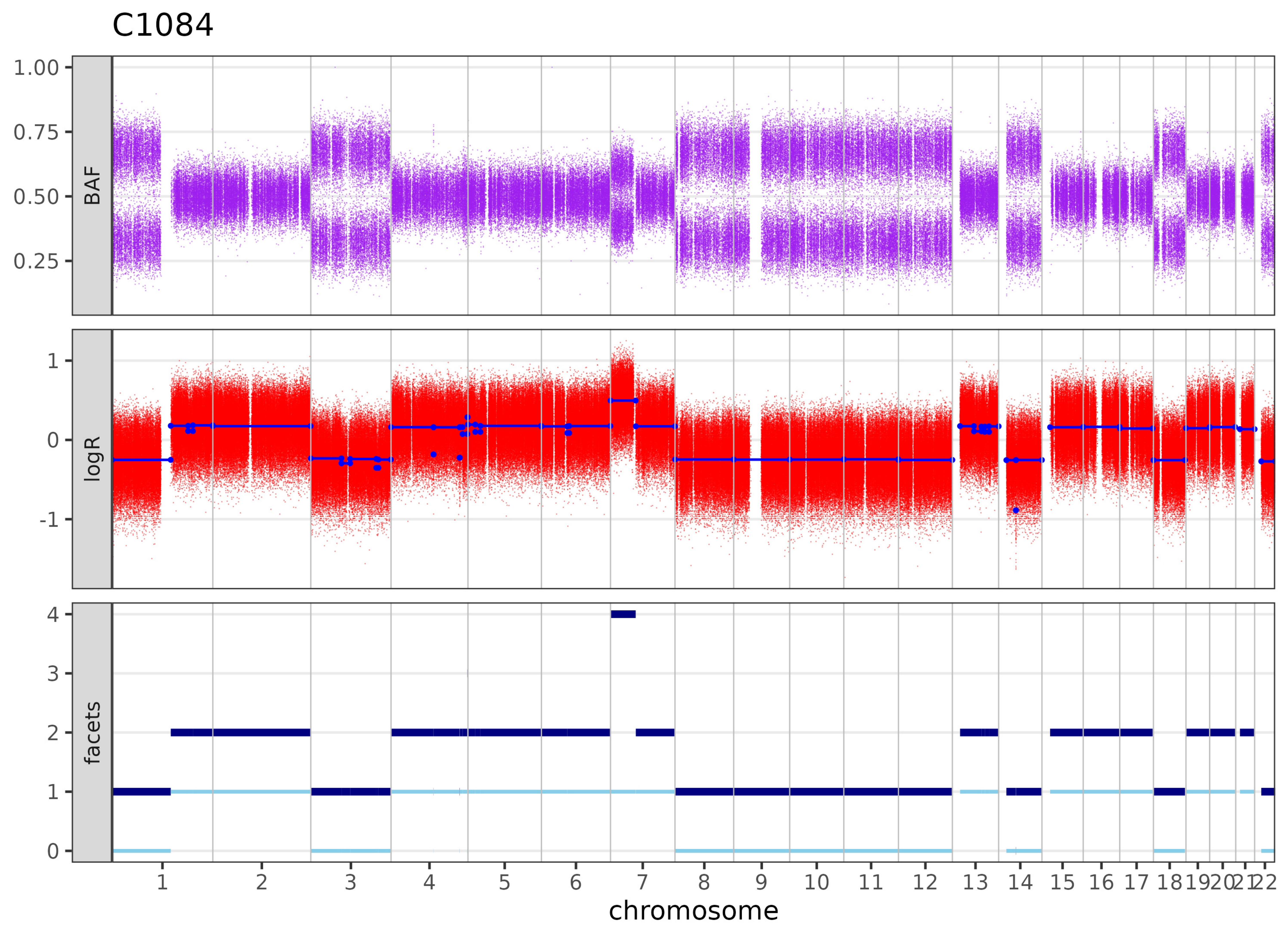
C1084
BAF
logR
facets
chromosome
1.00
0.75
0.50
0.25
1
0
-1
4
3
2
1
0
1 2 3 4 5 6 7 8 9 10 11 12 13 14 15 16 17 18 19 20 21 22

C1172

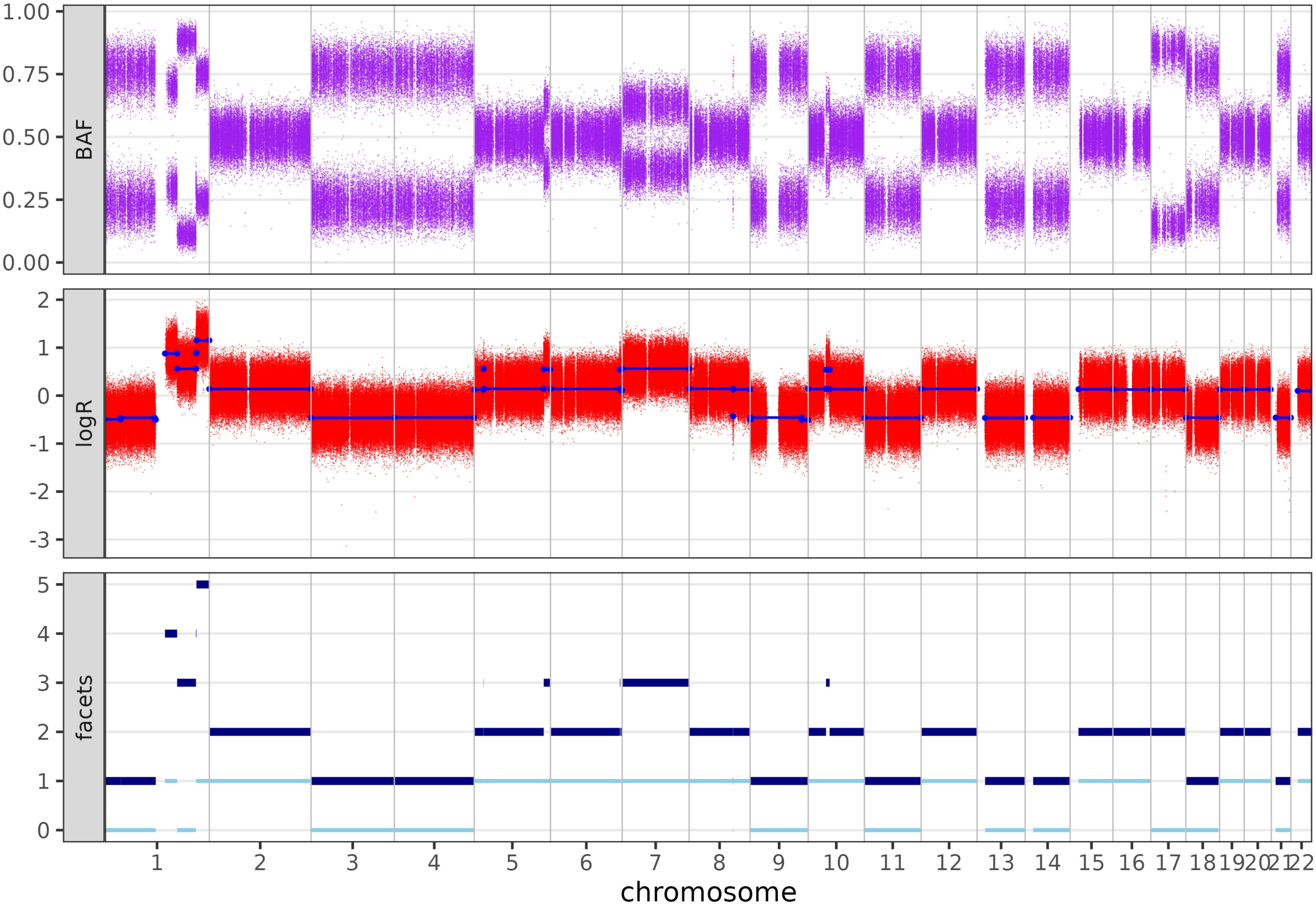

BAF
logR
facets
1.00
0.75
0.50
0.25
0.00
2
1
0
-1
-2
-3
5
4
3
2
1
0
1
2
3
4
5
6
7
8
9
10
11
12
13
14
15
16
17
18
19
20
21
22
chromosome

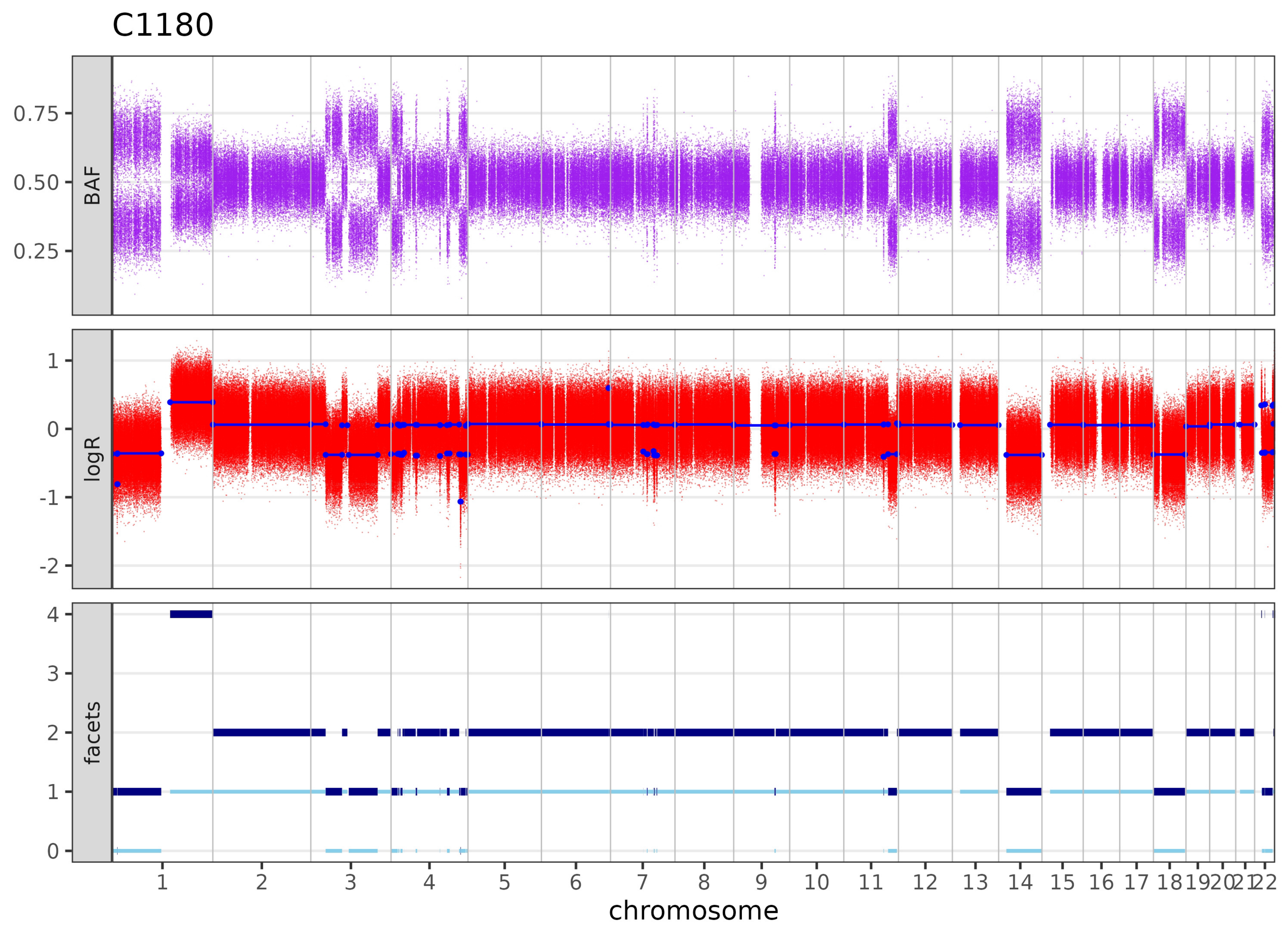
C1180
BAF
0.75
0.50
0.25
logR
1
0
-1
-2
facets
4
3
2
1
0
1 2 3 4 5 6 7 8 9 10 11 12 13 14 15 16 17 18 19 20 21 22
chromosome

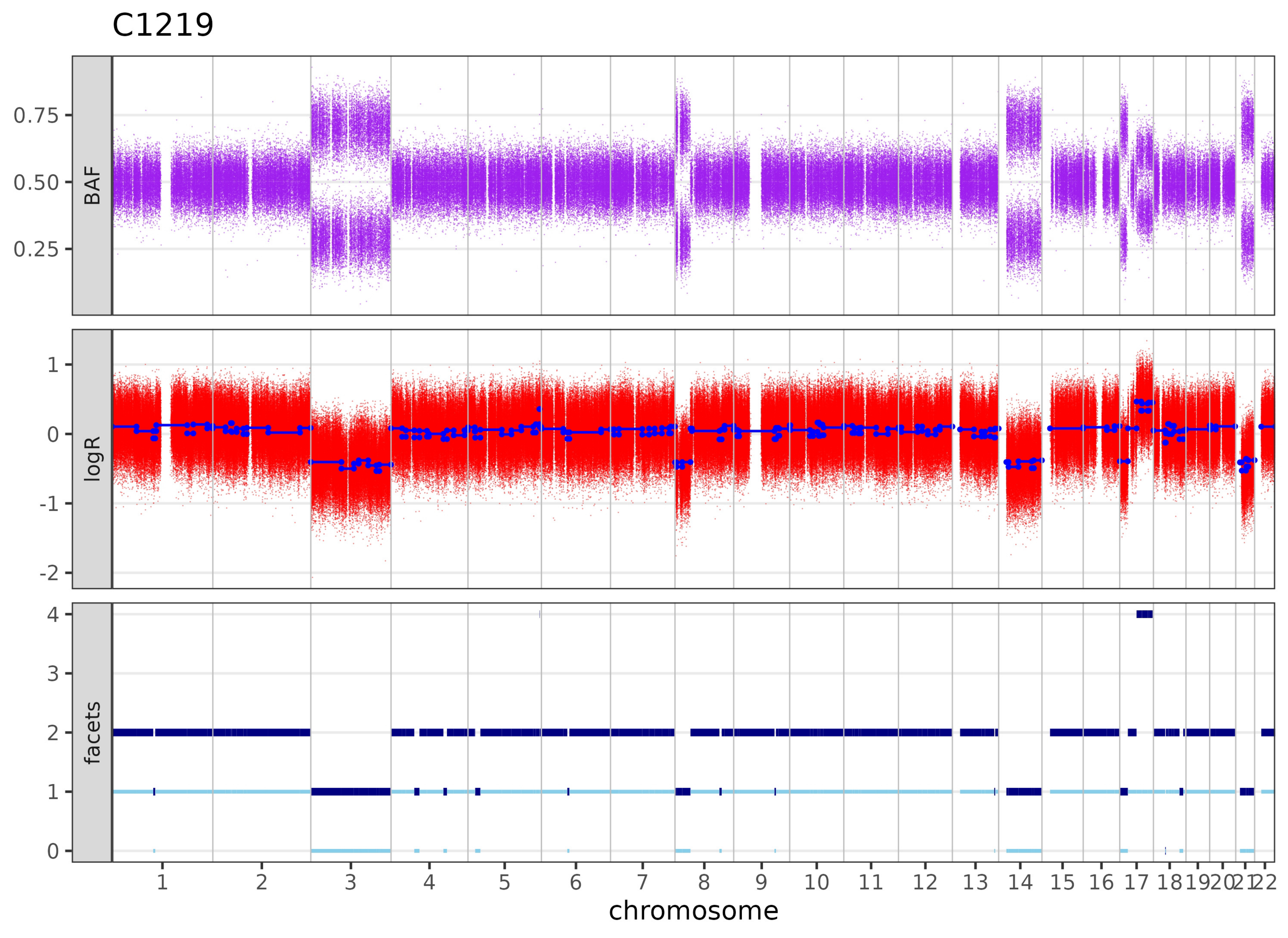
C1219
BAF
0.75
0.50
0.25
logR
1
0
-1
-2
facets
4
3
2
1
0
1
2
3
4
5
6
7
8
9
10
11
12
13
14
15
16
17
18
19
20
21
22
chromosome

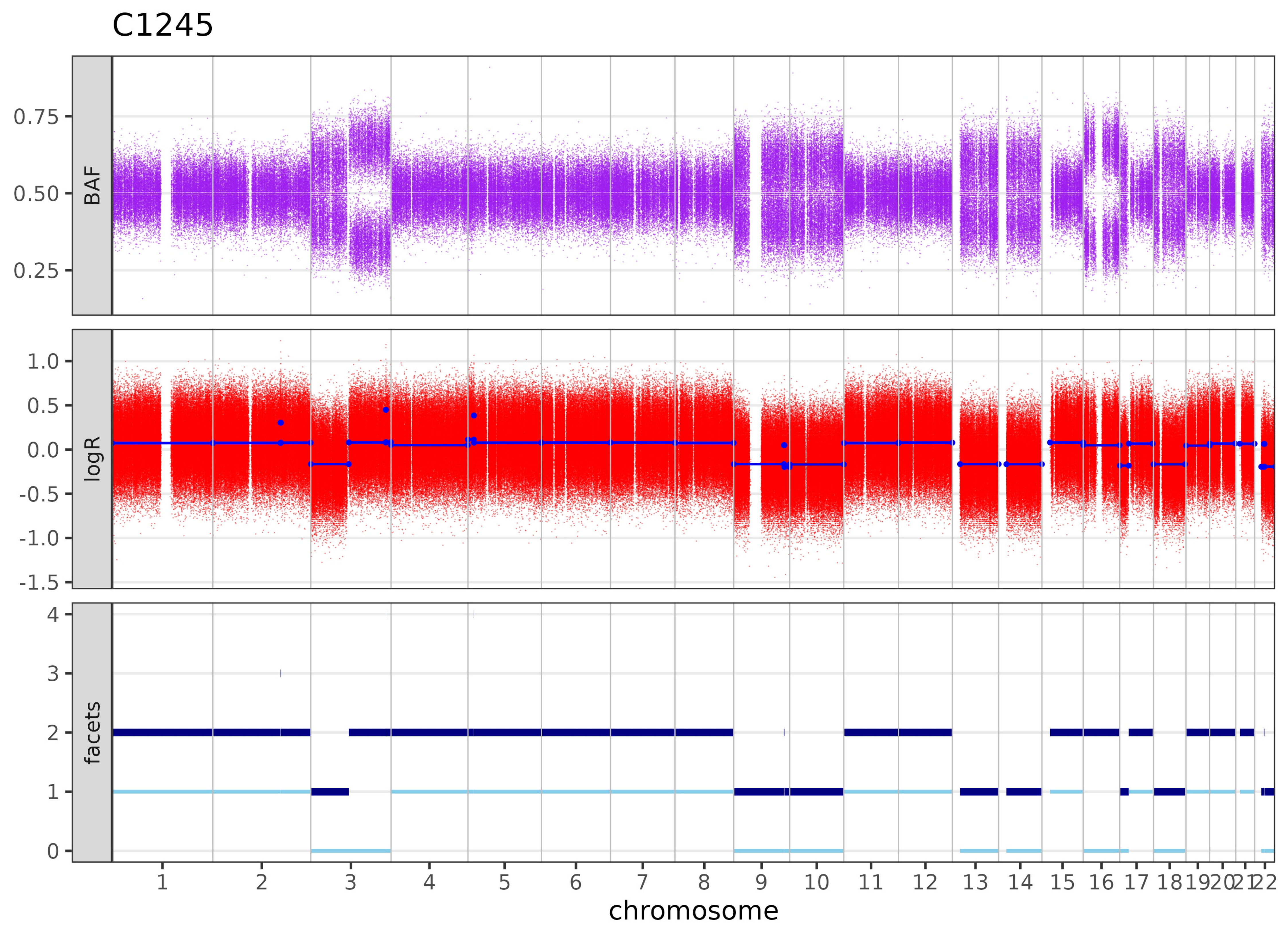
C1245
BAF
0.75
0.50
0.25
logR
1.0
0.5
0.0
-0.5
-1.0
-1.5
facets
4
3
2
1
0
1 2 3 4 5 6 7 8 9 10 11 12 13 14 15 16 17 18 19 20 21 22
chromosome

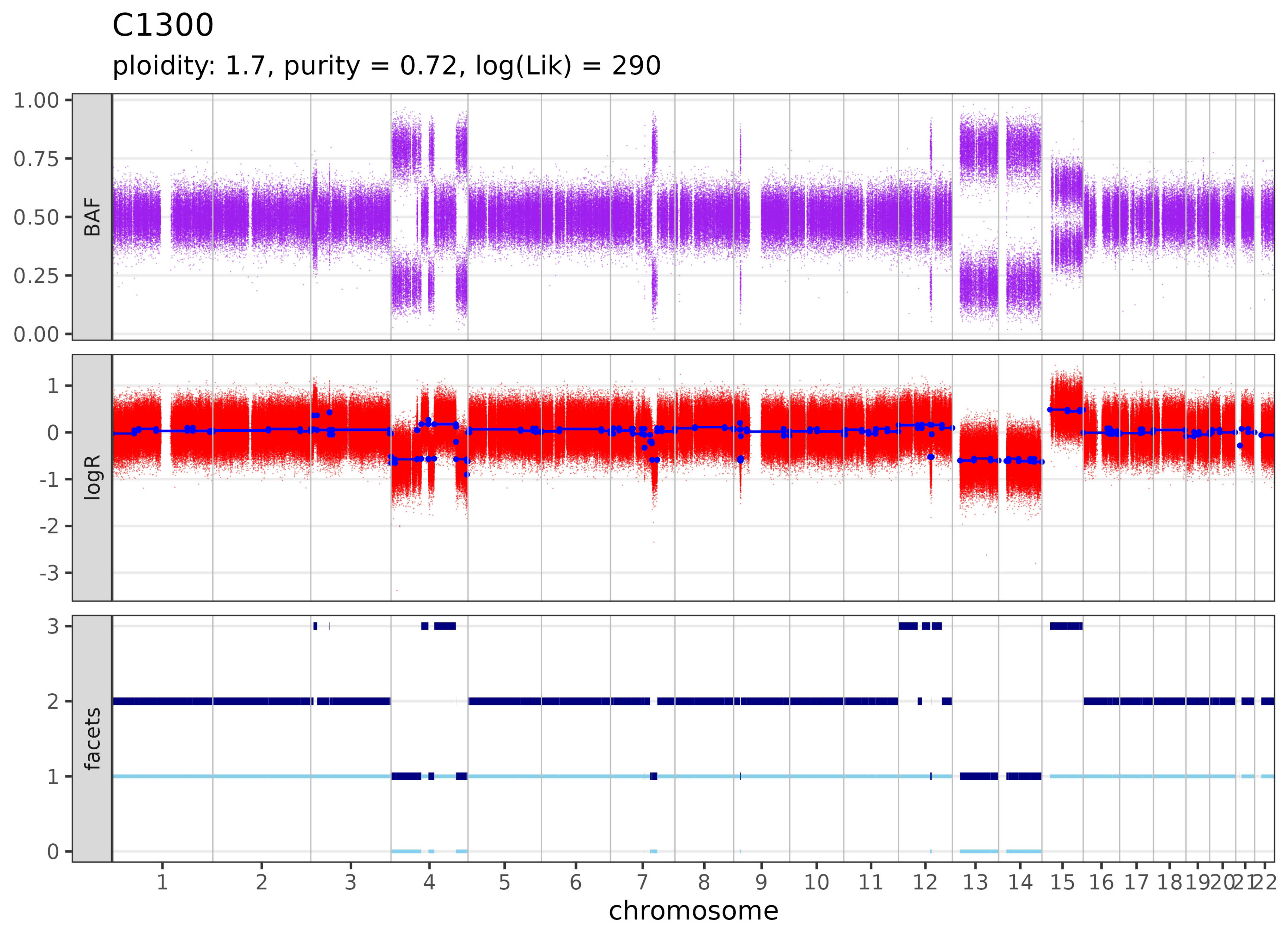

C1300
ploidity: 1.7, purity = 0.72, log(Lik) = 290
BAF
logR
facets
1.00
0.75
0.50
0.25
0.00
1
0
-1
-2
-3
3
2
1
0
1 2 3 4 5 6 7 8 9 10 11 12 13 14 15 16 17 18 19 20 21 22
chromosome

C1303

Insufficient information to estimate purity. Likely diplod or purity too low.

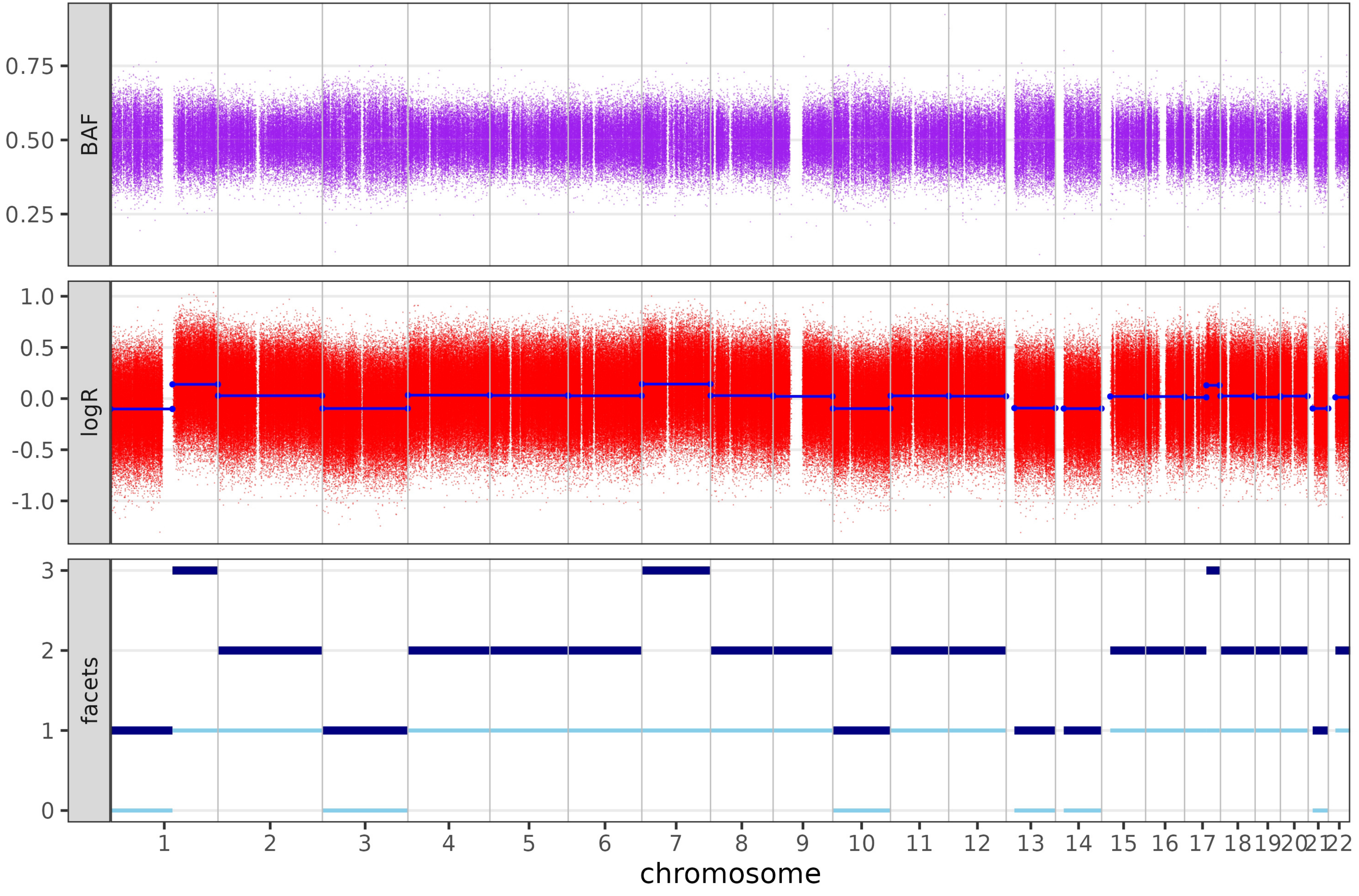

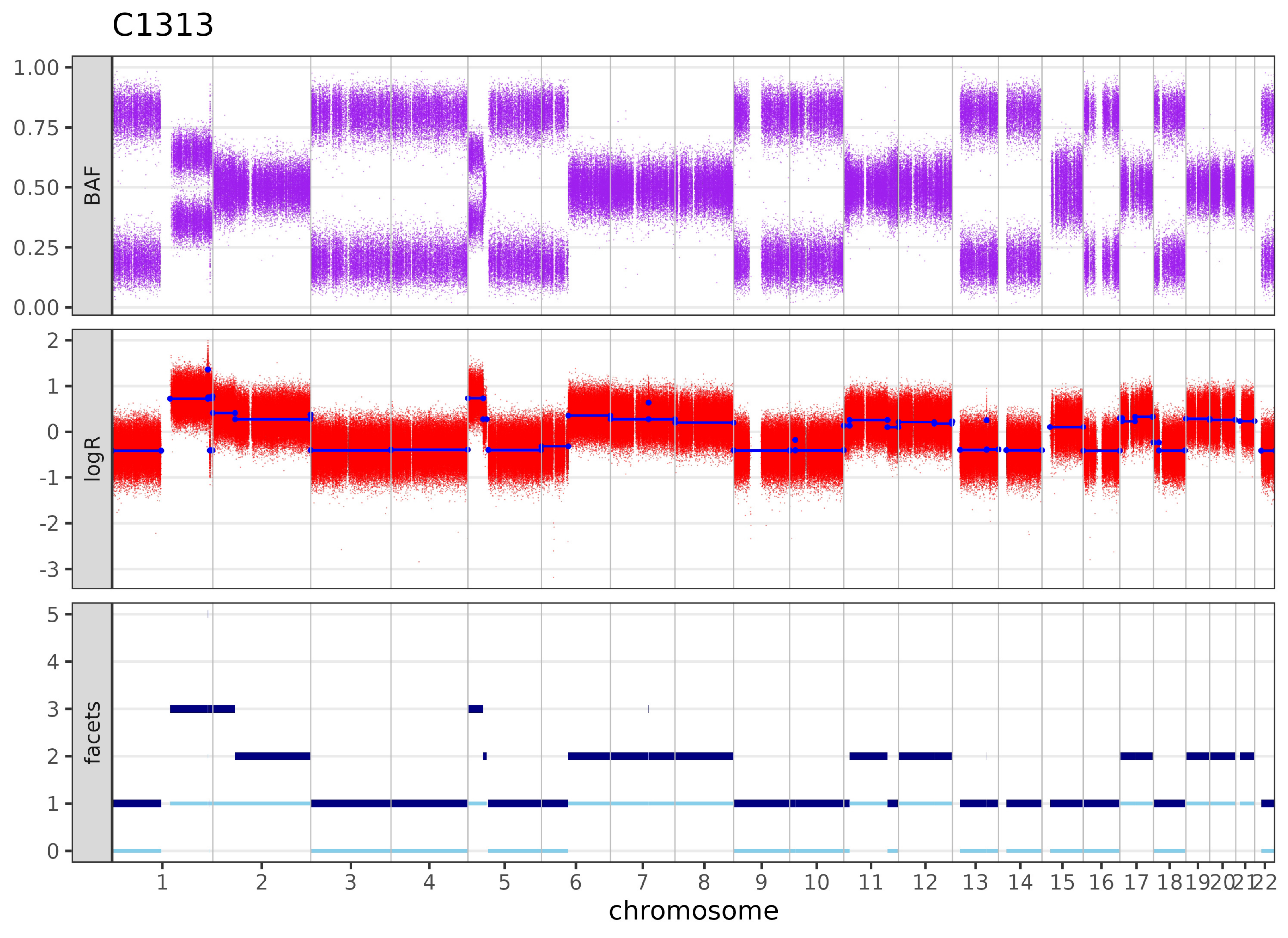
C1313
BAF
1.00
0.75
0.50
0.25
0.00
logR
2
1
0
-1
-2
-3
facets
5
4
3
2
1
0
1 2 3 4 5 6 7 8 9 10 11 12 13 14 15 16 17 18 19 20 21 22
chromosome

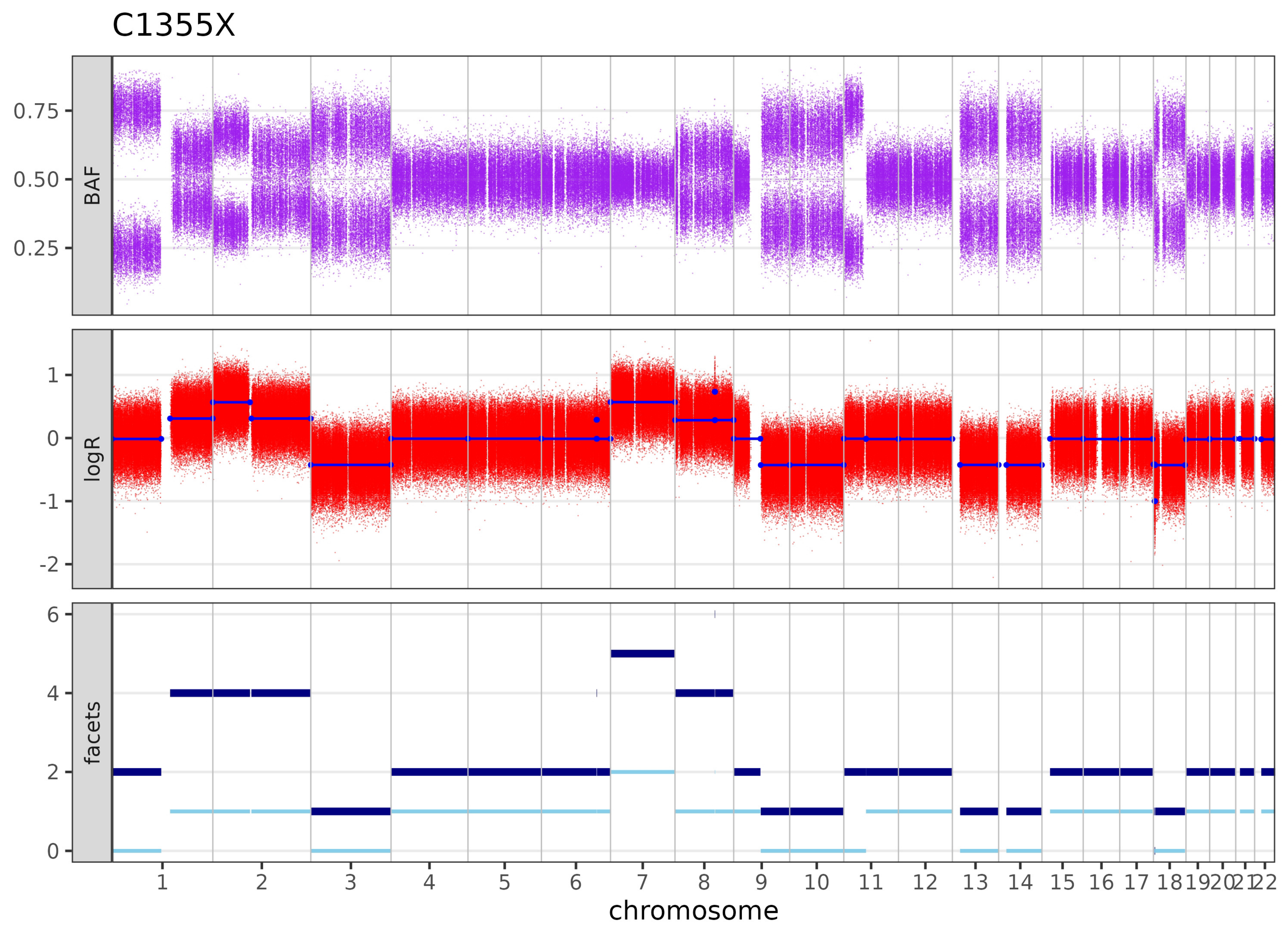
C1355X
BAF
0.75
0.50
0.25
logR
1
0
-1
-2
facets
6
4
2
0
1
2
3
4
5
6
7
8
9
10
11
12
13
14
15
16
17
18
19
20
21
22
chromosome

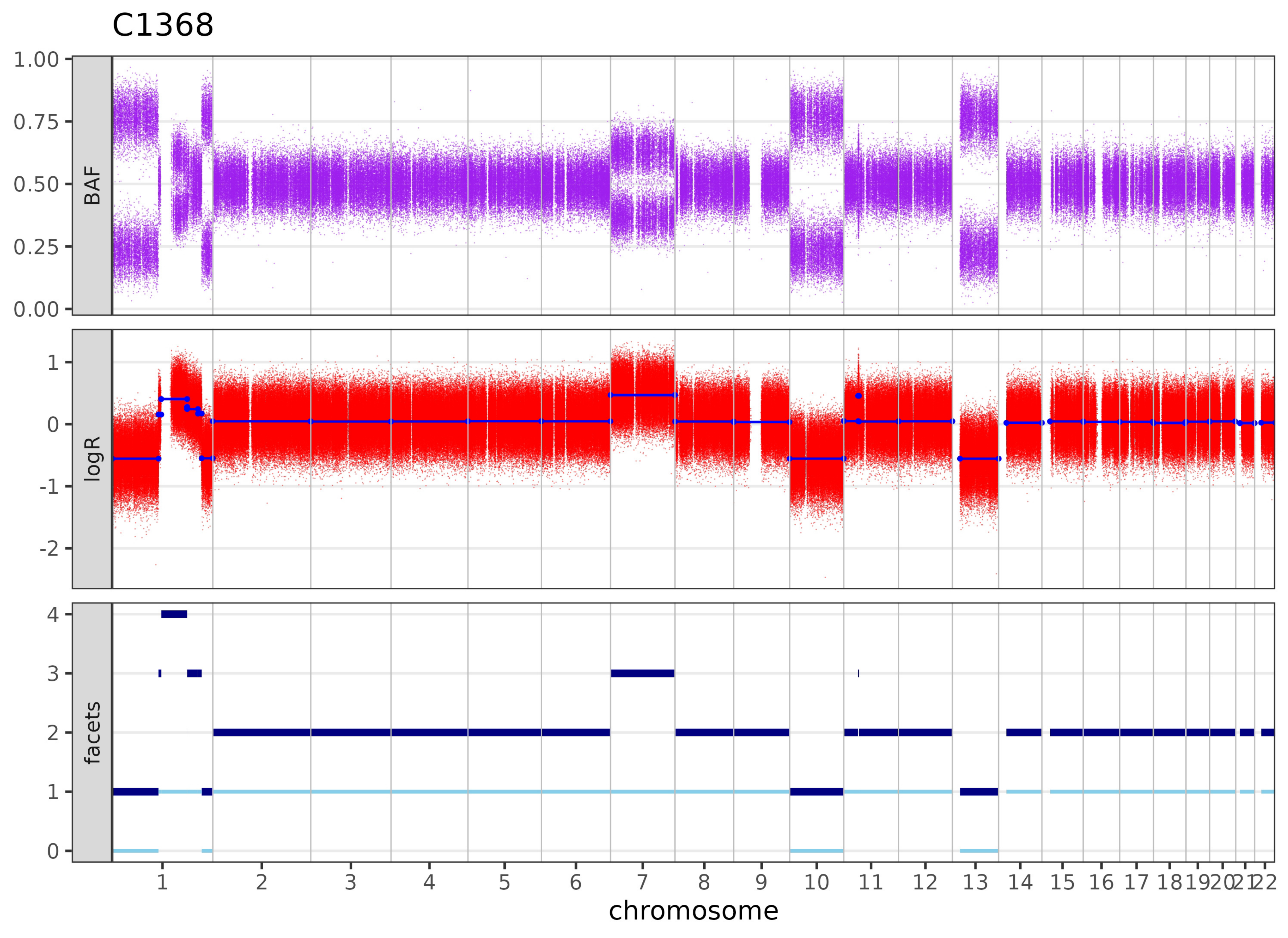
C1368
BAF
1.00
0.75
0.50
0.25
0.00
logR
1
0
-1
-2
facets
4
3
2
1
0
1 2 3 4 5 6 7 8 9 10 11 12 13 14 15 16 17 18 19 20 21 22
chromosome

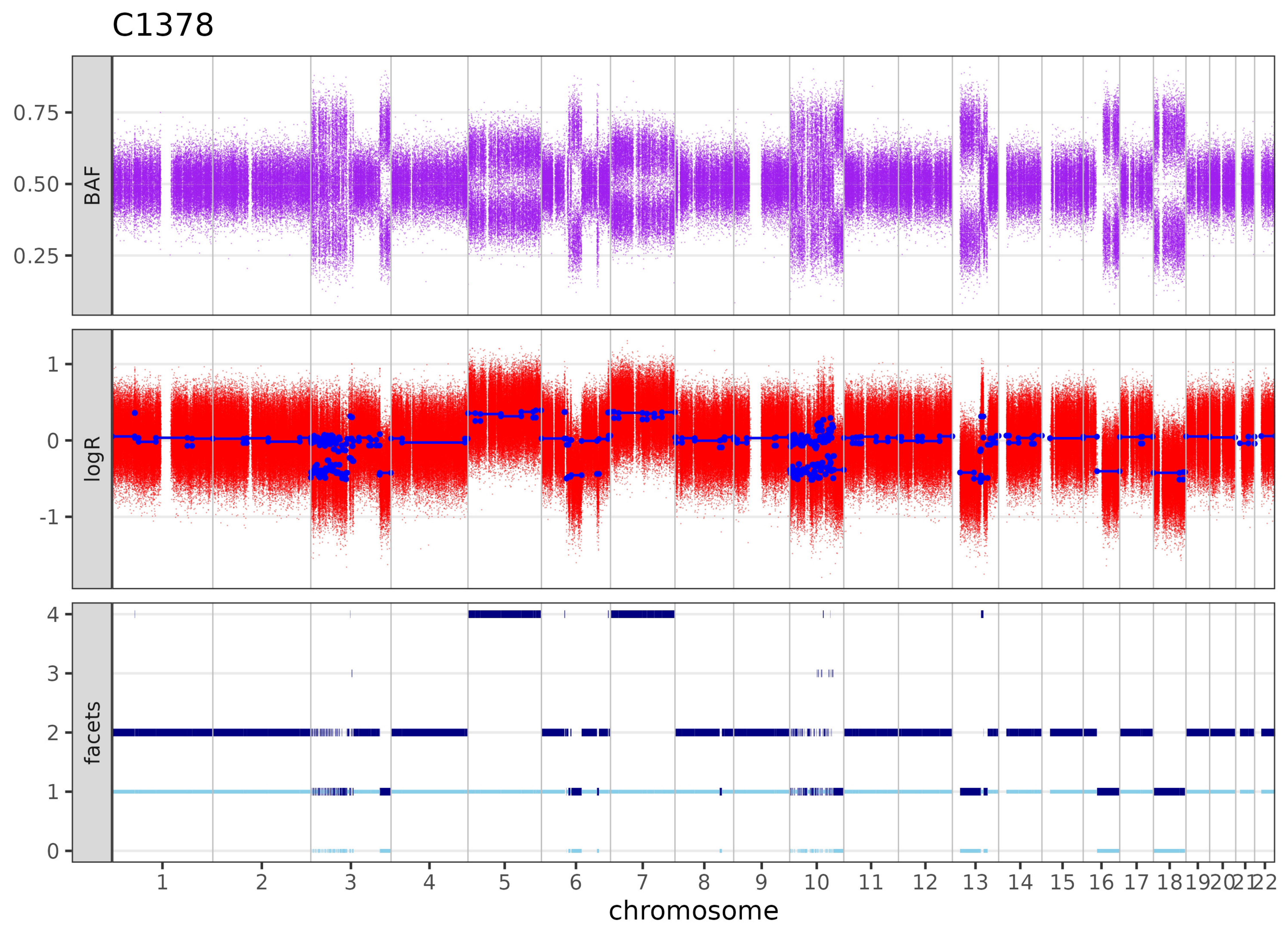
C1378
BAF
0.75
0.50
0.25
logR
1
0
-1
facets
4
3
2
1
0
1
2
3
4
5
6
7
8
9
10
11
12
13
14
15
16
17
18
19
20
21
22
chromosome

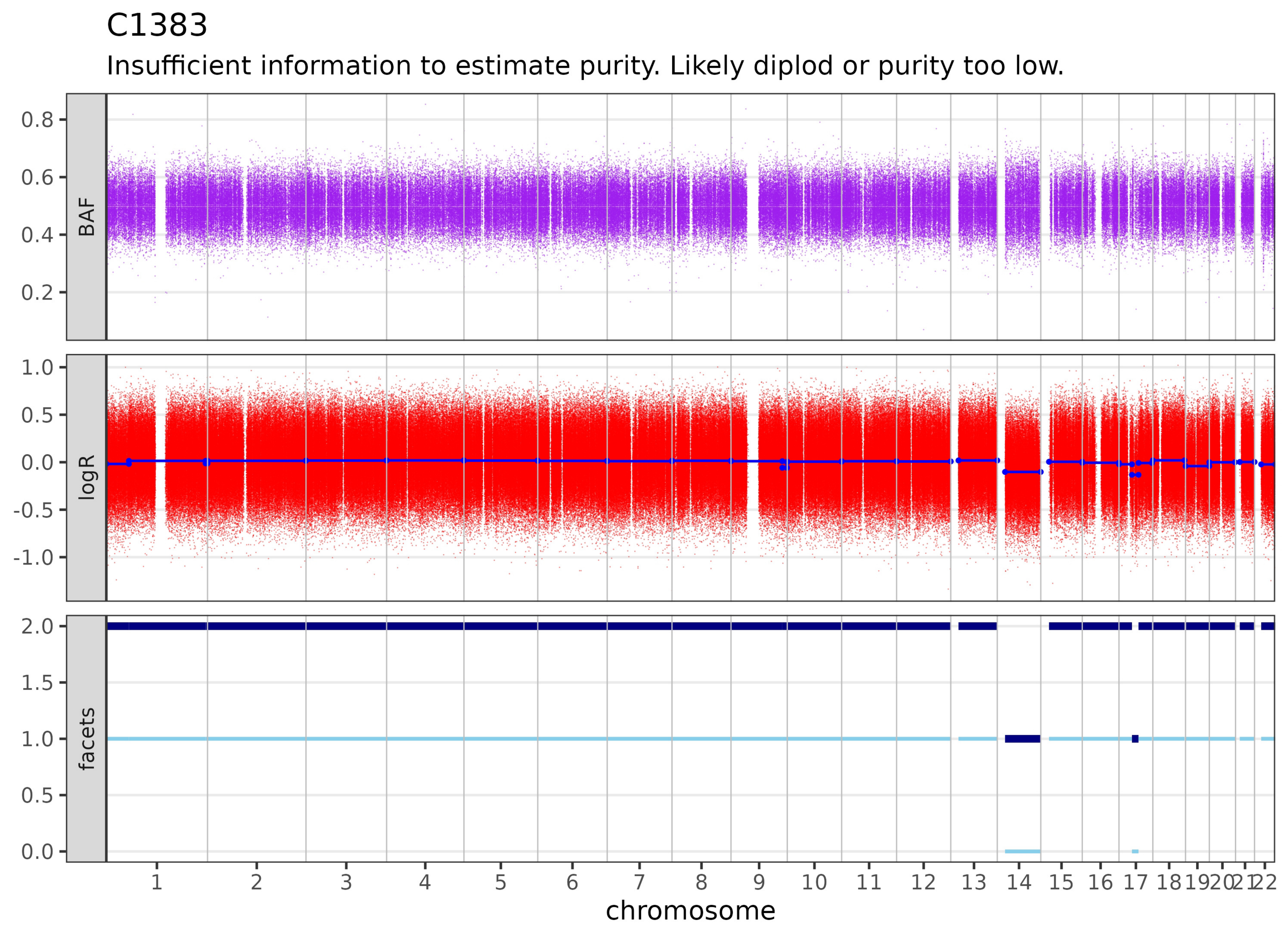
C1383
Insufficient information to estimate purity. Likely diplod or purity too low.
BAF
0.8
0.6
0.4
0.2
logR
1.0
0.5
0.0
-0.5
-1.0
facets
2.0
1.5
1.0
0.5
0.0
1 2 3 4 5 6 7 8 9 10 11 12 13 14 15 16 17 18 19 20 21 22
chromosome

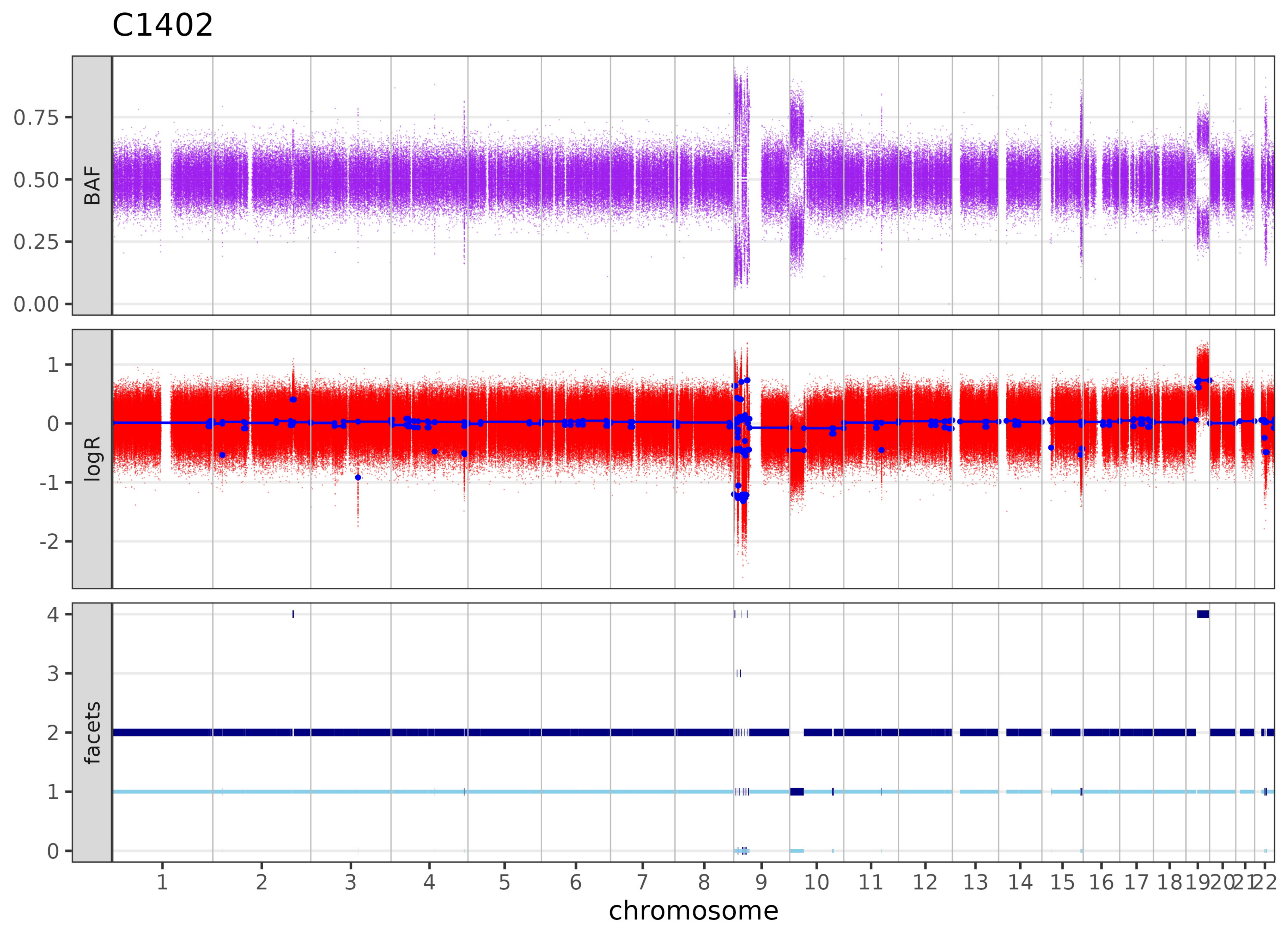
C1402
BAF
0.75
0.50
0.25
0.00
logR
1
0
-1
-2
facets
4
3
2
1
0
1
2
3
4
5
6
7
8
9
10
11
12
13
14
15
16
17
18
19
20
21
22
chromosome

C1417

ploidity: 2.4, purity = 0.55, log(Lik) = 640

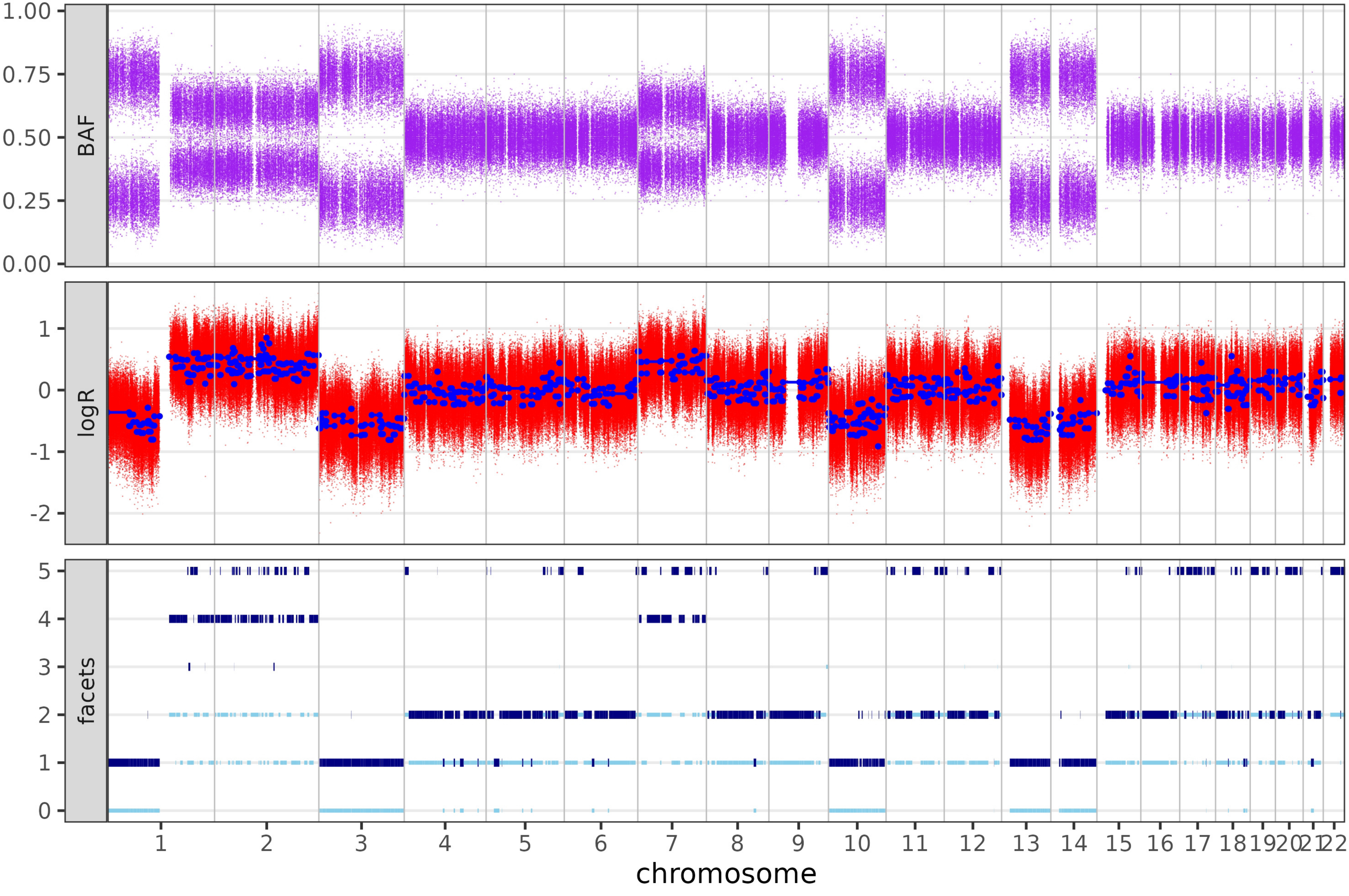

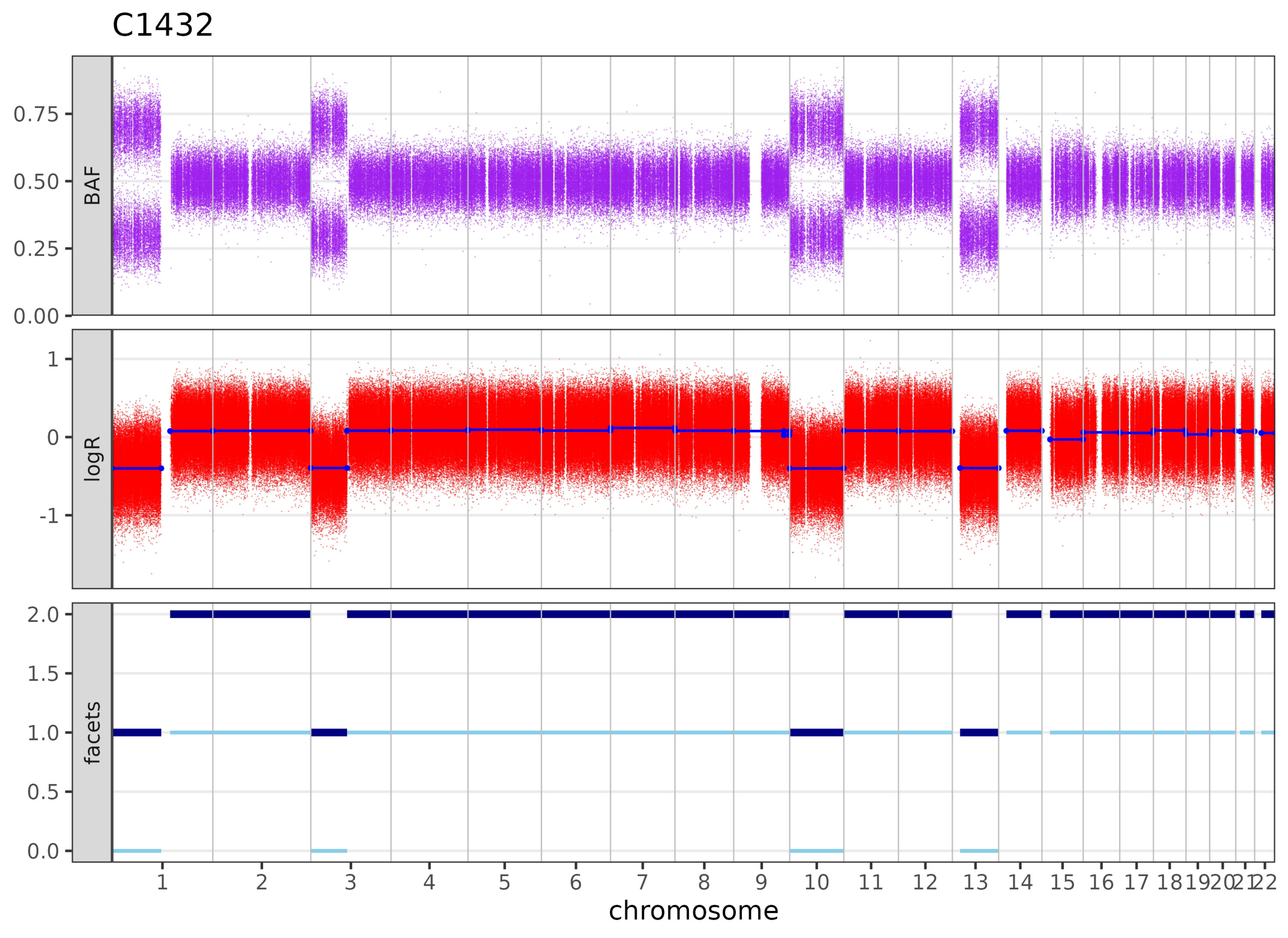

C1432
BAF
0.00
0.25
0.50
0.75
logR
1
0
-1
facets
0.0
0.5
1.0
1.5
2.0
1 2 3 4 5 6 7 8 9 10 11 12 13 14 15 16 17 18 19 20 21 22
chromosome

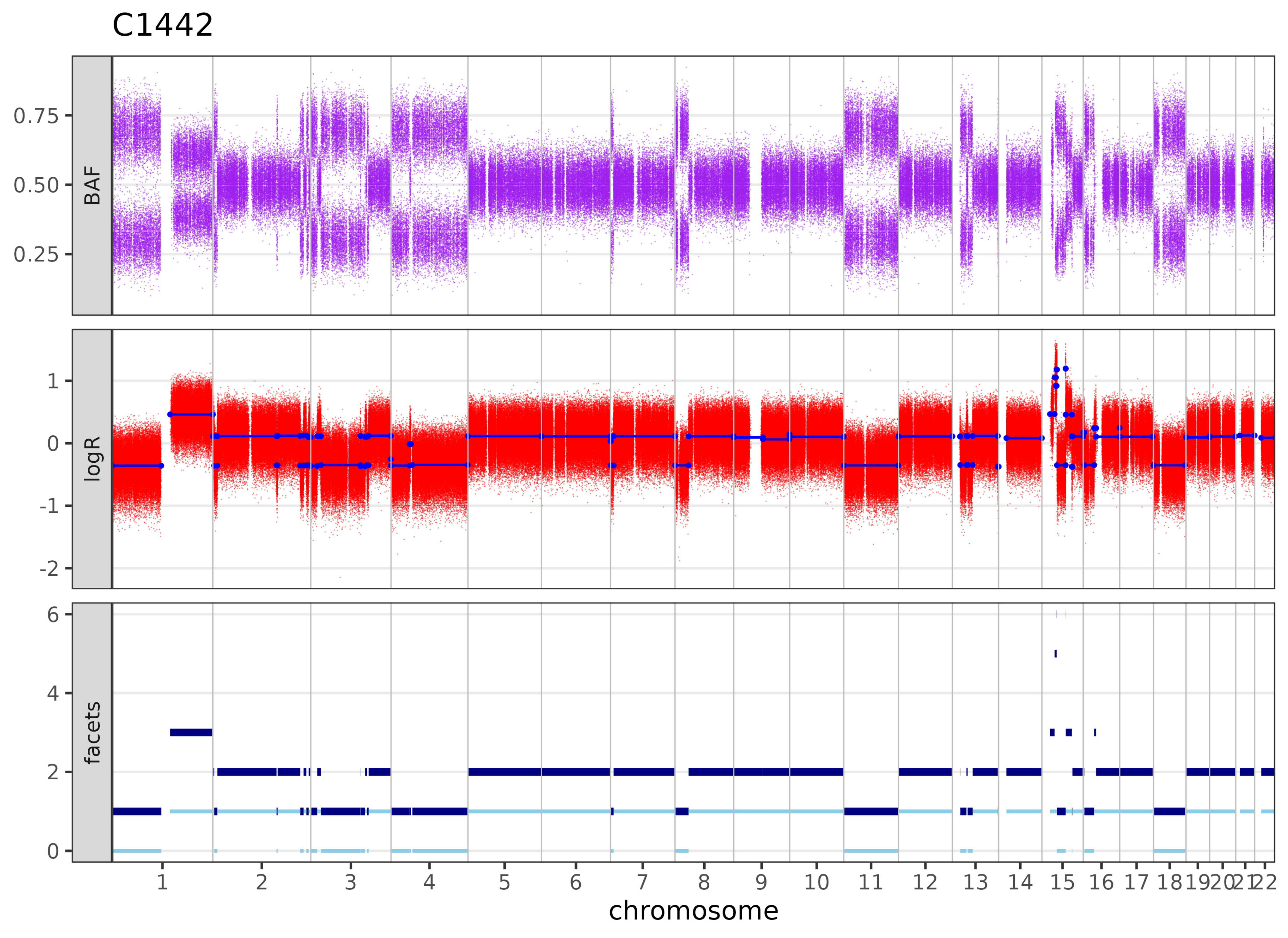
C1442
BAF
0.75
0.50
0.25
logR
1
0
-1
-2
facets
6
4
2
0
1
2
3
4
5
6
7
8
9
10
11
12
13
14
15
16
17
18
19
20
21
22
chromosome

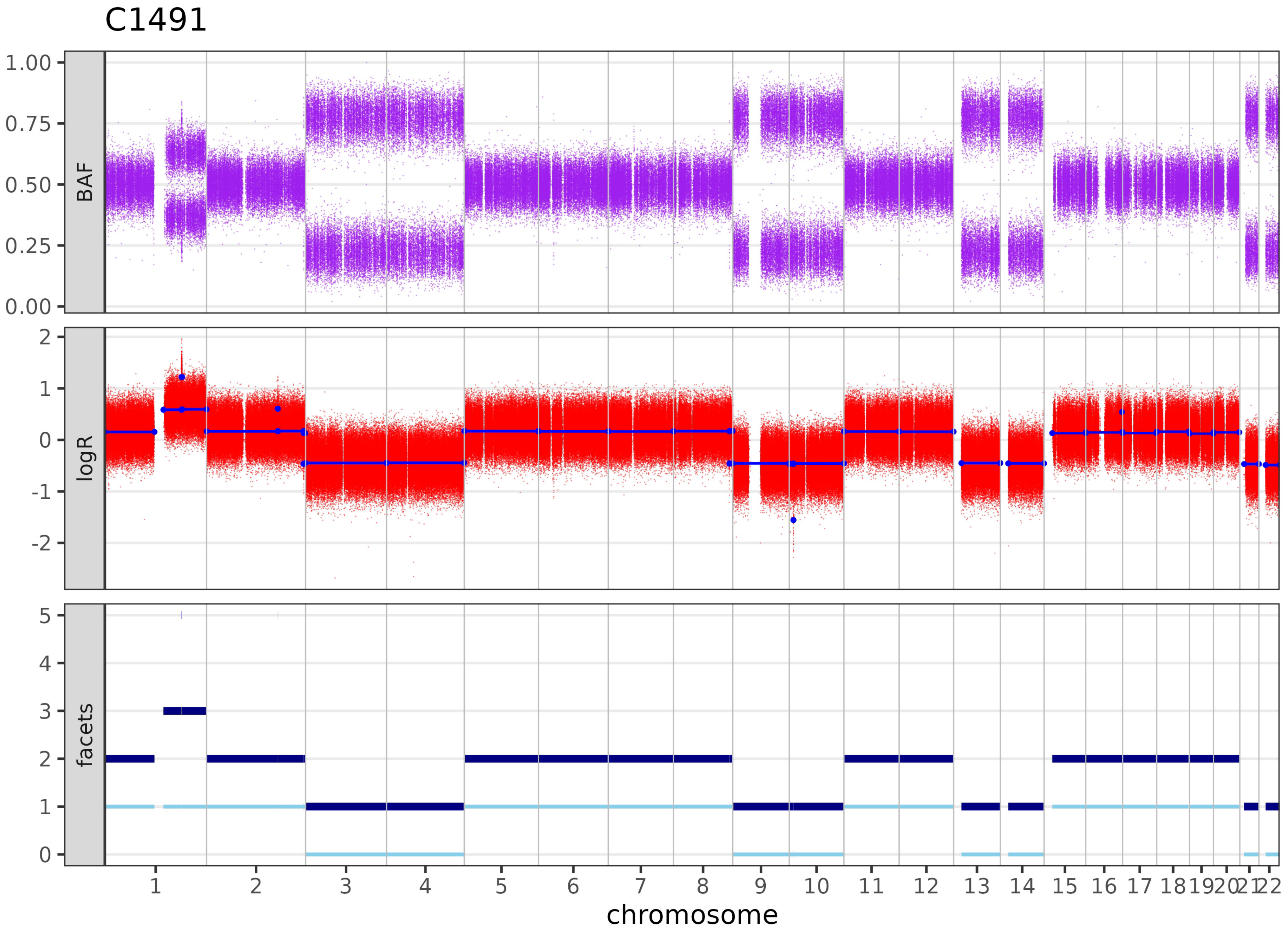
C1491
BAF
1.00
0.75
0.50
0.25
0.00
logR
2
1
0
-1
-2
facets
5
4
3
2
1
0
1 2 3 4 5 6 7 8 9 10 11 12 13 14 15 16 17 18 19 20 21 22
chromosome

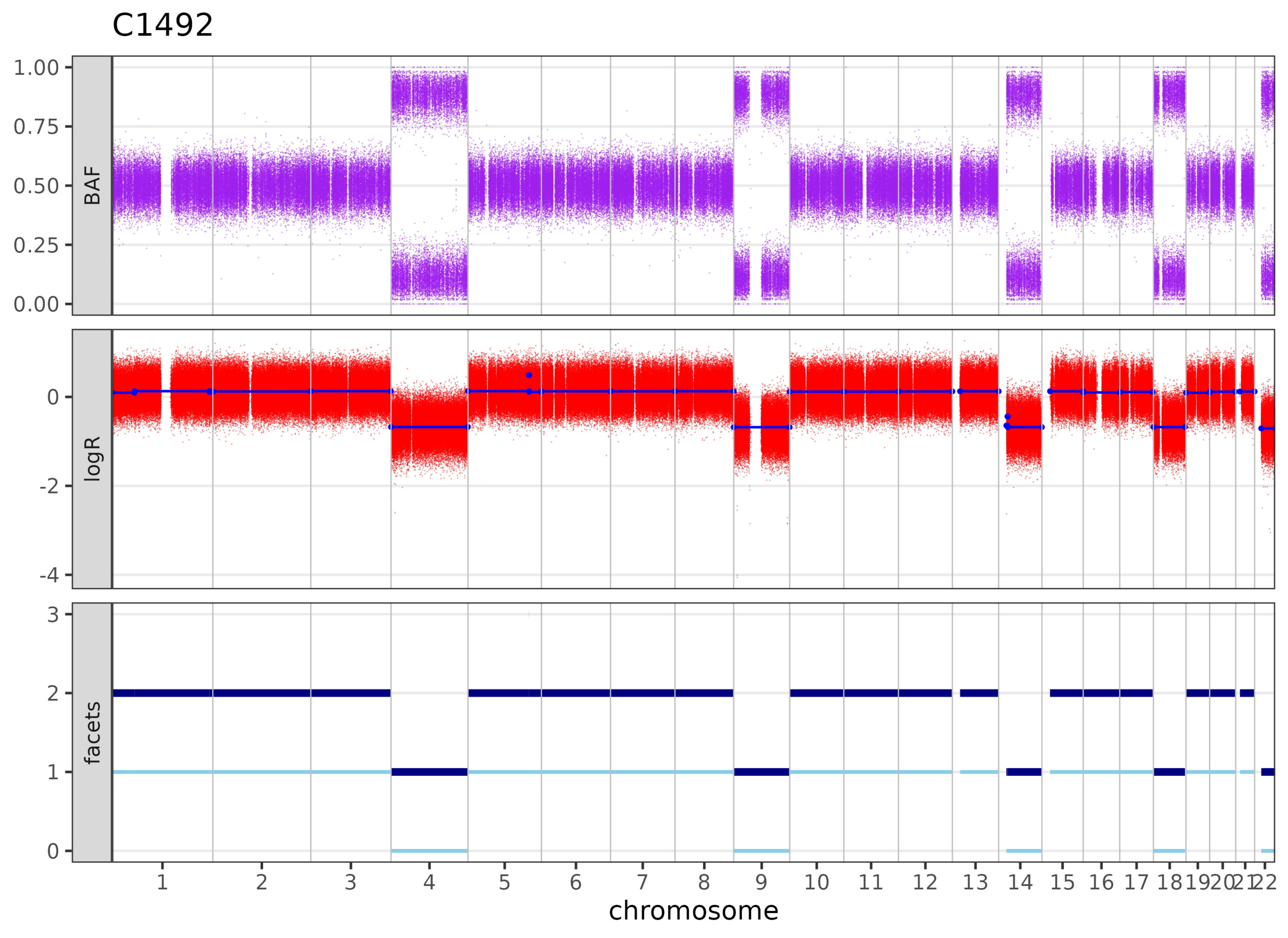

C1492
BAF
logR
facets
1.00
0.75
0.50
0.25
0.00
0
-2
-4
3
2
1
0
1 2 3 4 5 6 7 8 9 10 11 12 13 14 15 16 17 18 19 20 21 22
chromosome

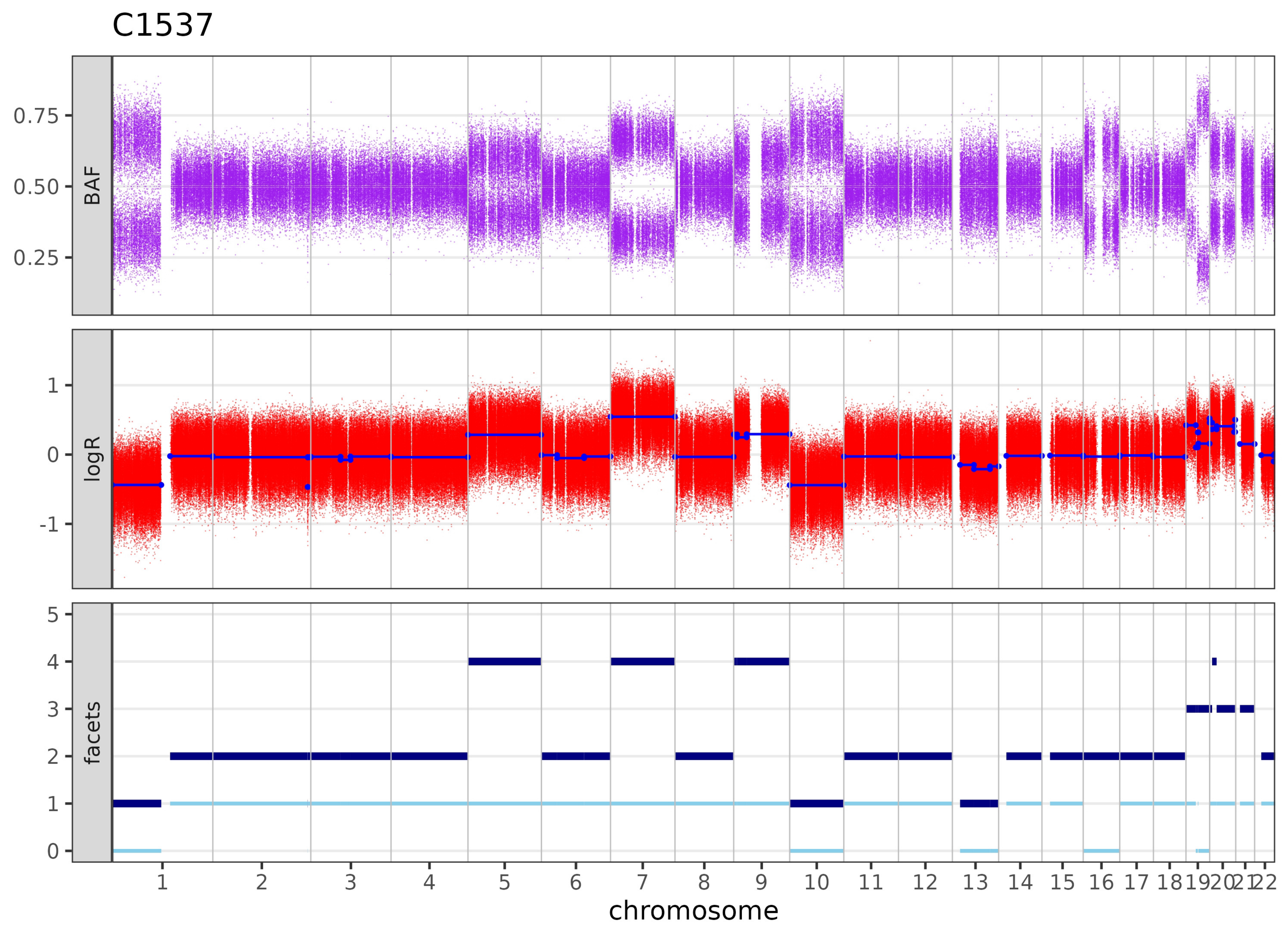
C1537
BAF
logR
facets
0.75
0.50
0.25
1
0
-1
5
4
3
2
1
0
1 2 3 4 5 6 7 8 9 10 11 12 13 14 15 16 17 18 19 20 21 22
chromosome

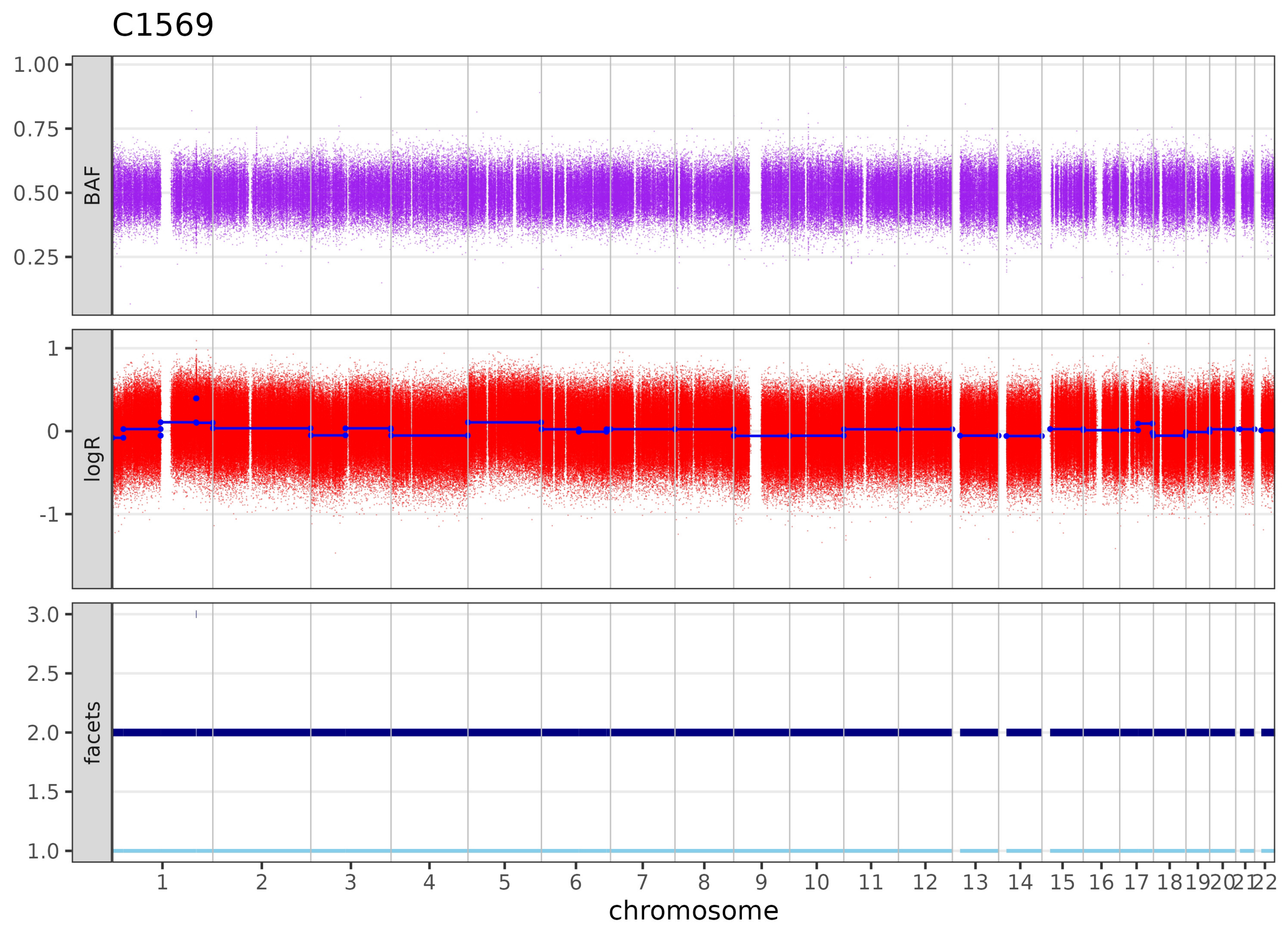
C1569
BAF
logR
facets
1.00
0.75
0.50
0.25
1
0
-1
3.0
2.5
2.0
1.5
1.0
1 2 3 4 5 6 7 8 9 10 11 12 13 14 15 16 17 18 19 20 21 22
chromosome

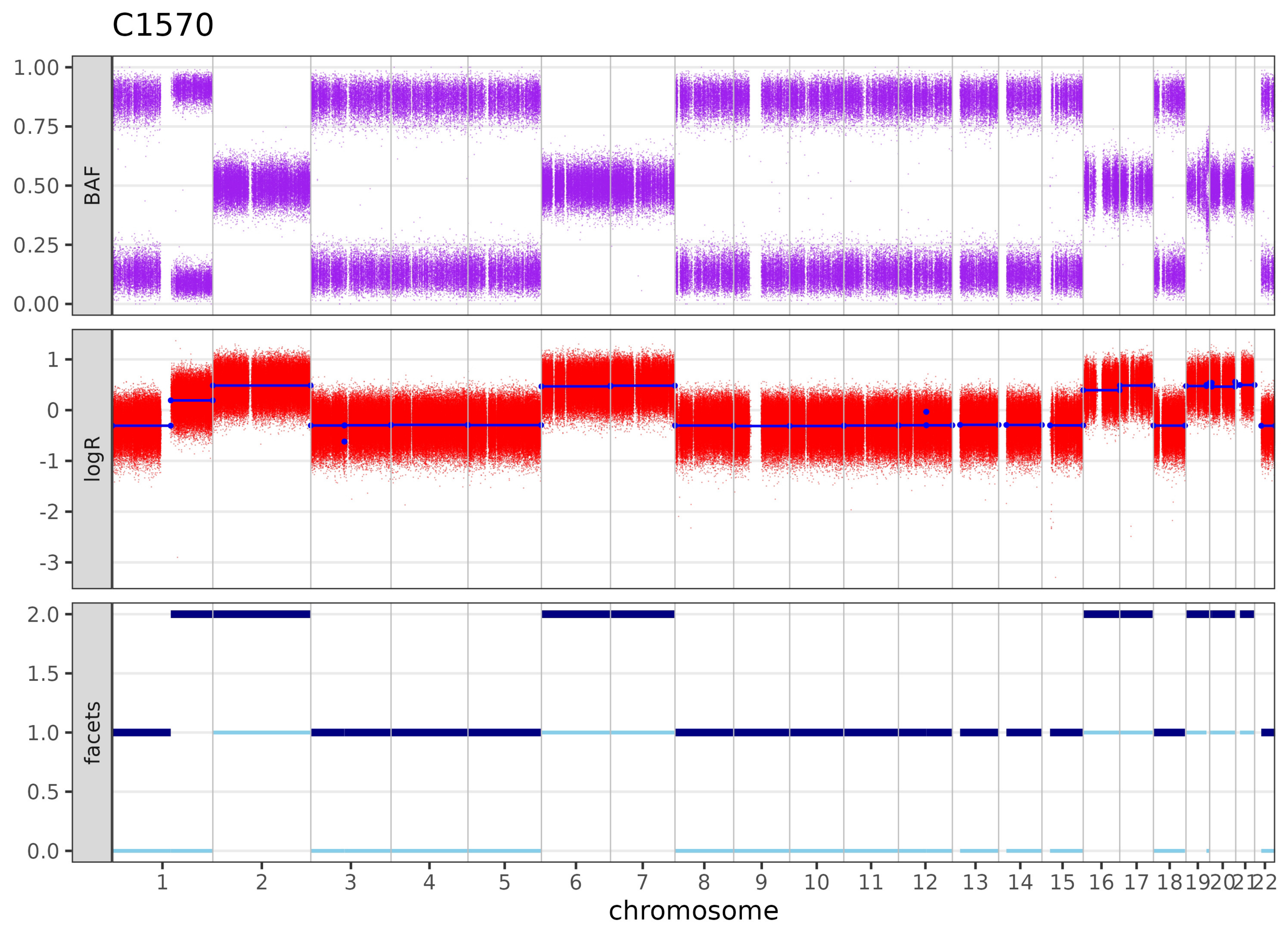
C1570
BAF
logR
facets
1.00
0.75
0.50
0.25
0.00
1
0
-1
-2
-3
2.0
1.5
1.0
0.5
0.0
1 2 3 4 5 6 7 8 9 10 11 12 13 14 15 16 17 18 19 20 21 22
chromosome

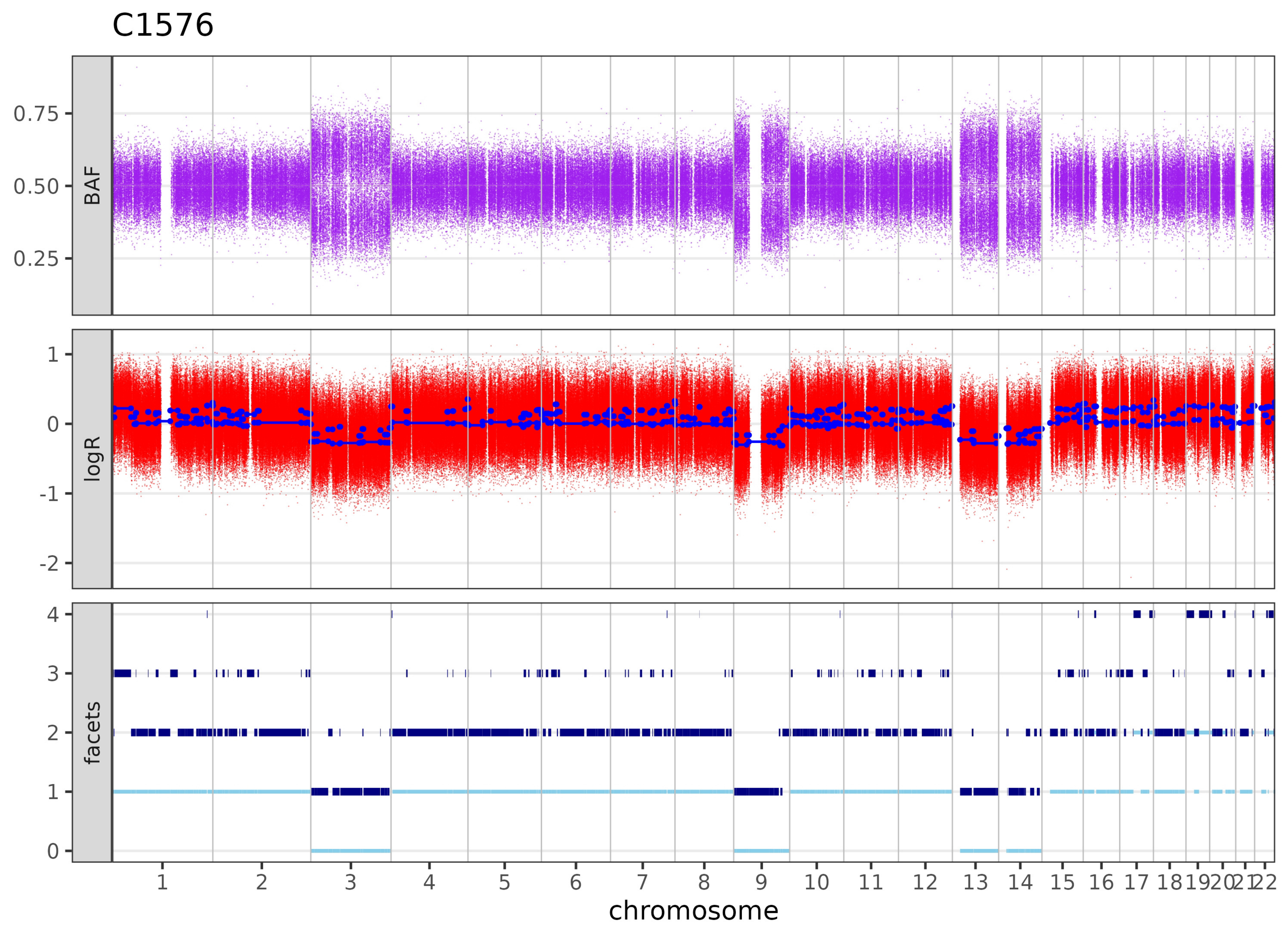
C1576
BAF
0.75
0.50
0.25
logR
1
0
-1
-2
facets
4
3
2
1
0
1 2 3 4 5 6 7 8 9 10 11 12 13 14 15 16 17 18 19 20 21 22
chromosome

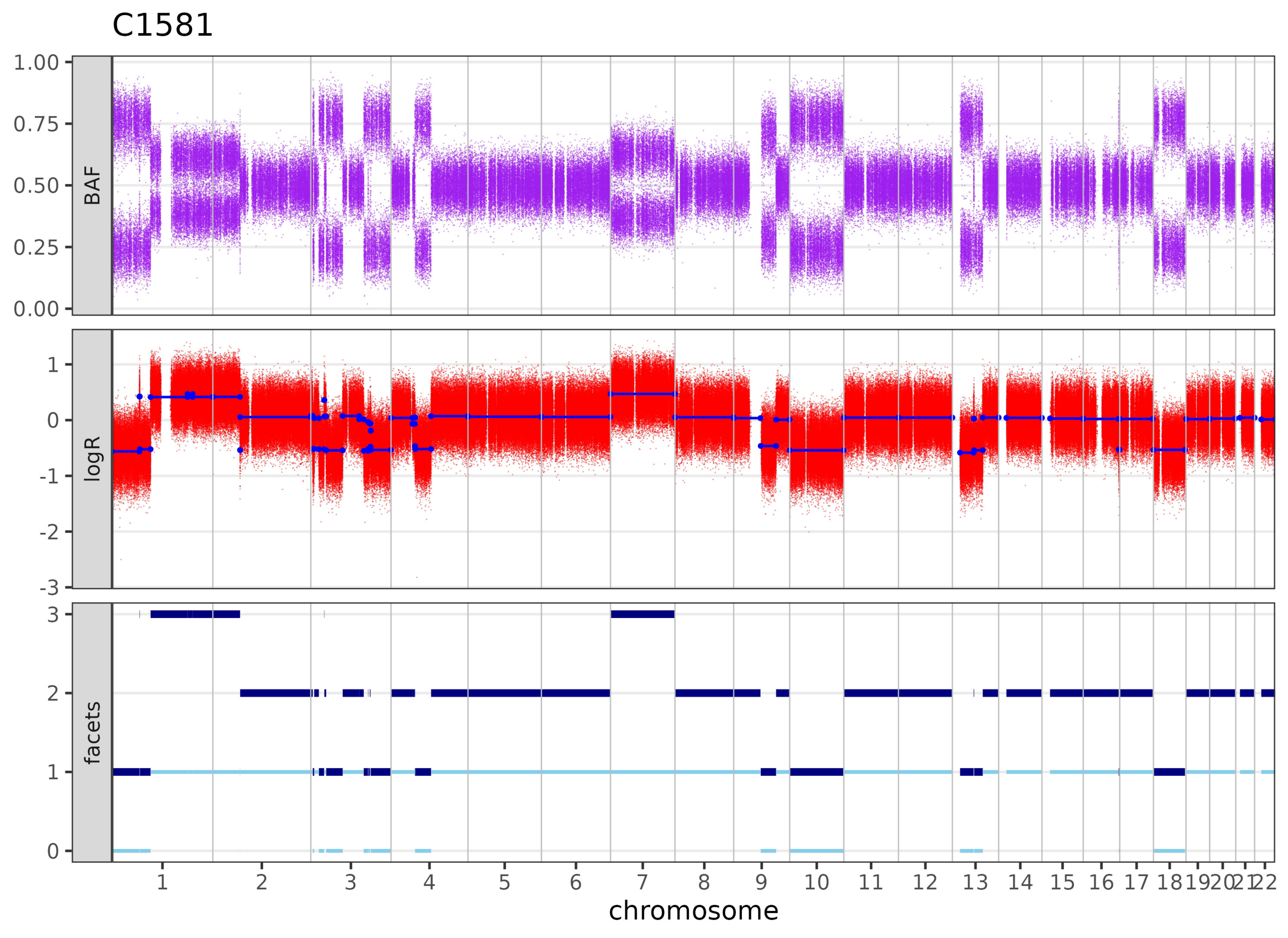
C1581
BAF
logR
facets
1.00
0.75
0.50
0.25
0.00
1
0
-1
-2
-3
3
2
1
0
1 2 3 4 5 6 7 8 9 10 11 12 13 14 15 16 17 18 19 20 21 22
chromosome

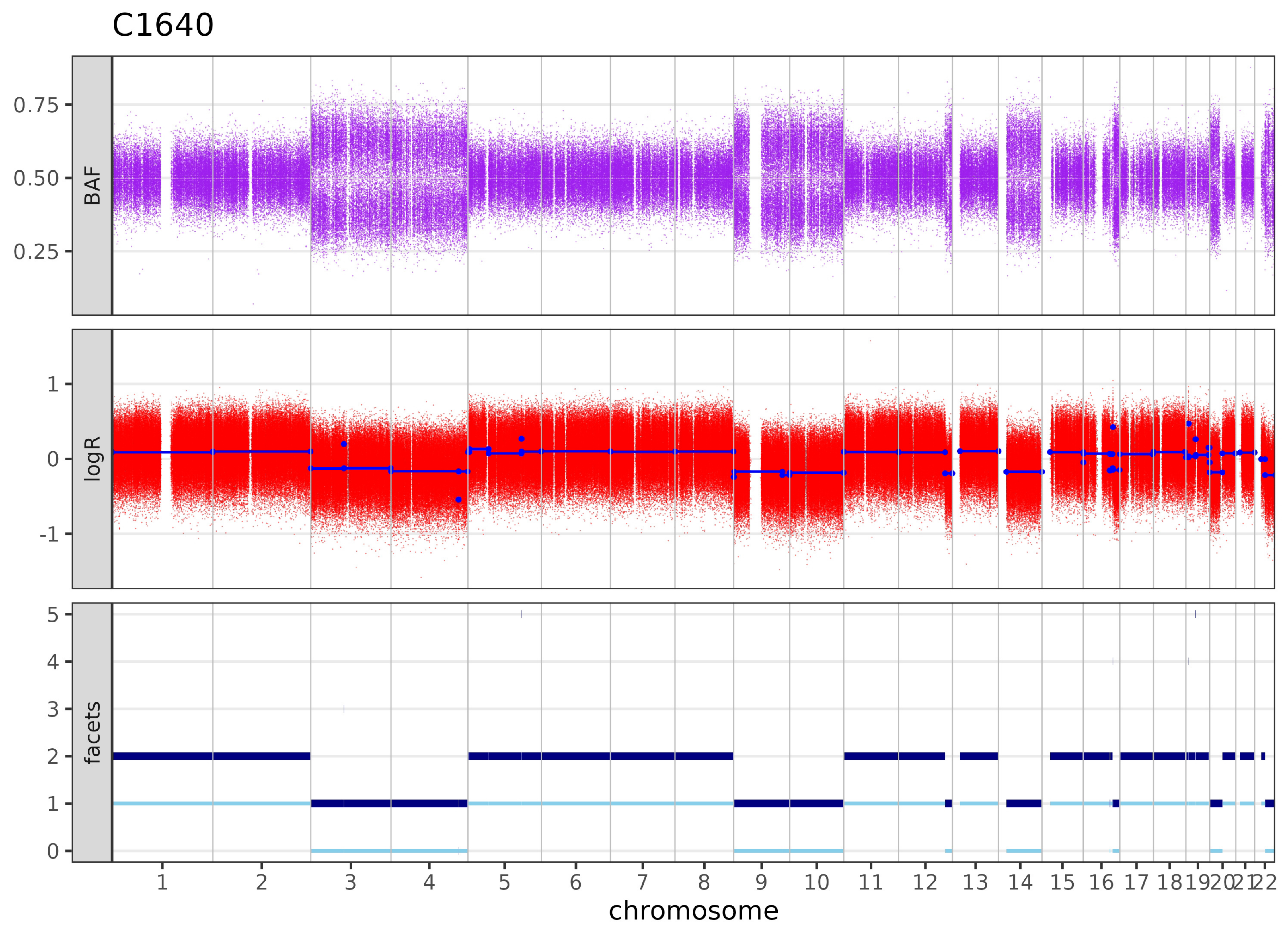
C1640
BAF
0.75
0.50
0.25
logR
1
0
-1
facets
5
4
3
2
1
0
1
2
3
4
5
6
7
8
9
10
11
12
13
14
15
16
17
18
19
20
21
22
chromosome

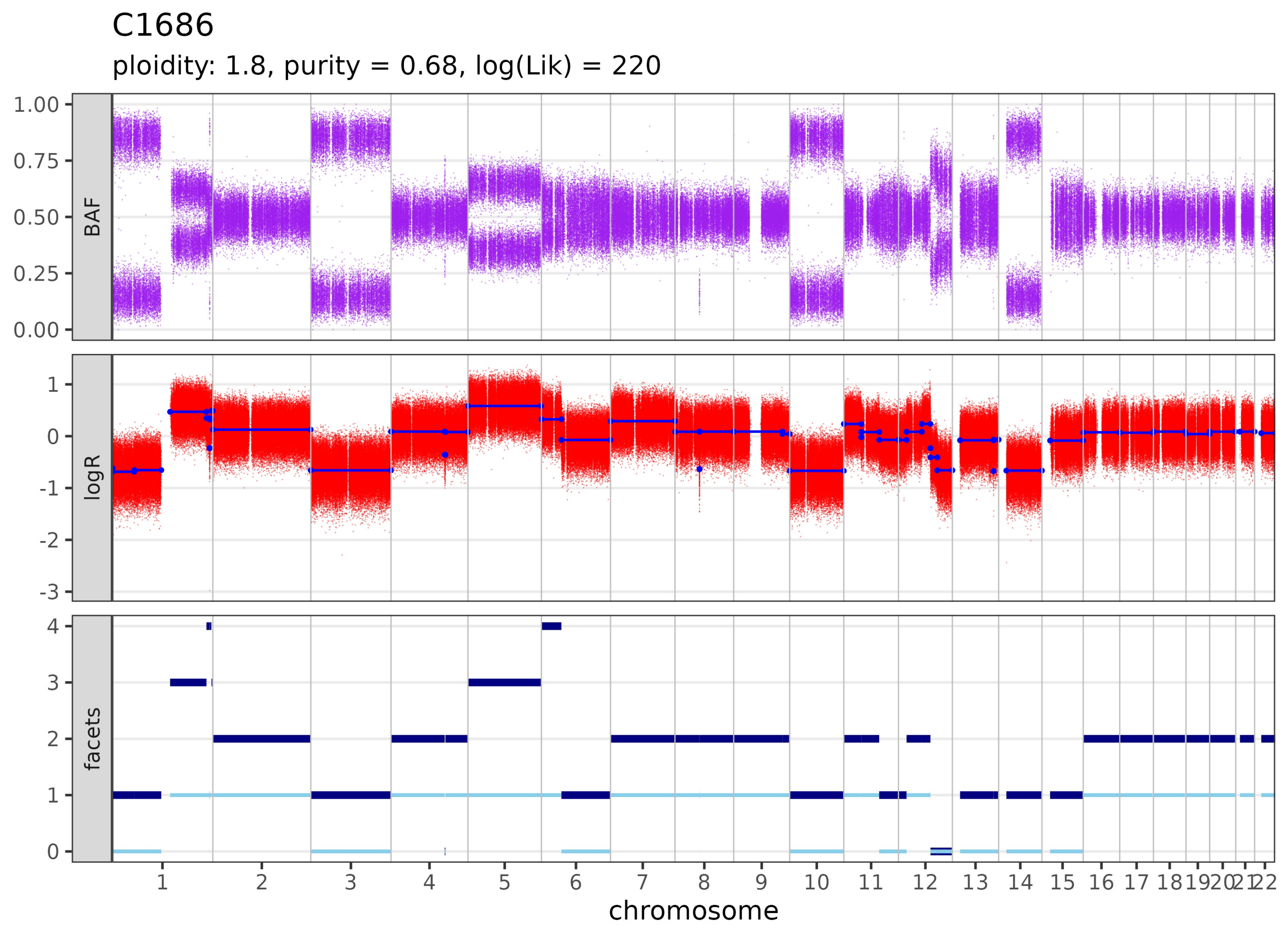
C1686
ploidity: 1.8, purity = 0.68, log(Lik) = 220
BAF
logR
facets
chromosome
1.00
0.75
0.50
0.25
0.00
1
0
-1
-2
-3
4
3
2
1
0
1 2 3 4 5 6 7 8 9 10 11 12 13 14 15 16 17 18 19 20 21 22

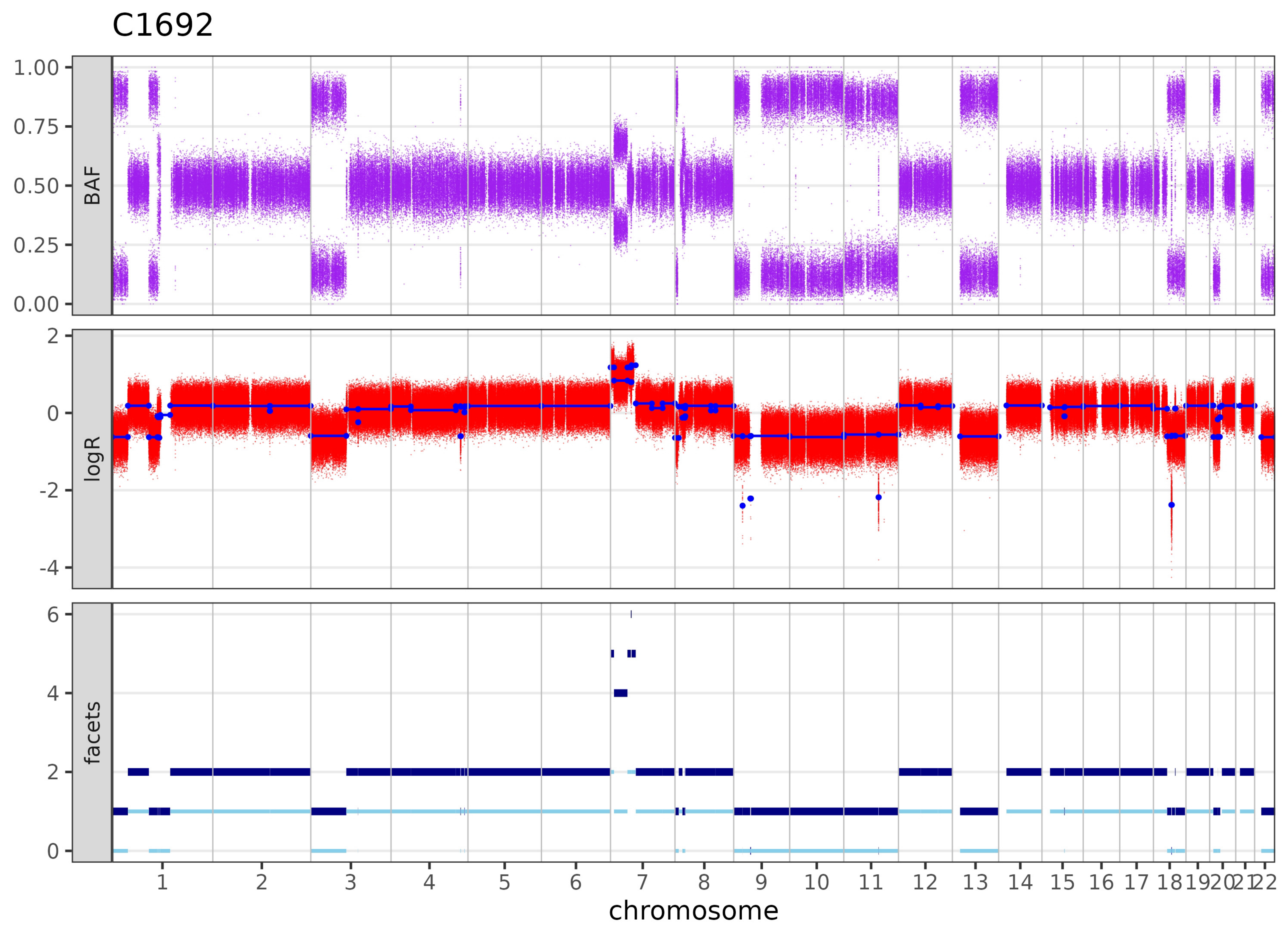

C1692
BAF
1.00
0.75
0.50
0.25
0.00
logR
2
0
-2
-4
facets
6
4
2
0
1
2
3
4
5
6
7
8
9
10
11
12
13
14
15
16
17
18
19
20
21
22
chromosome

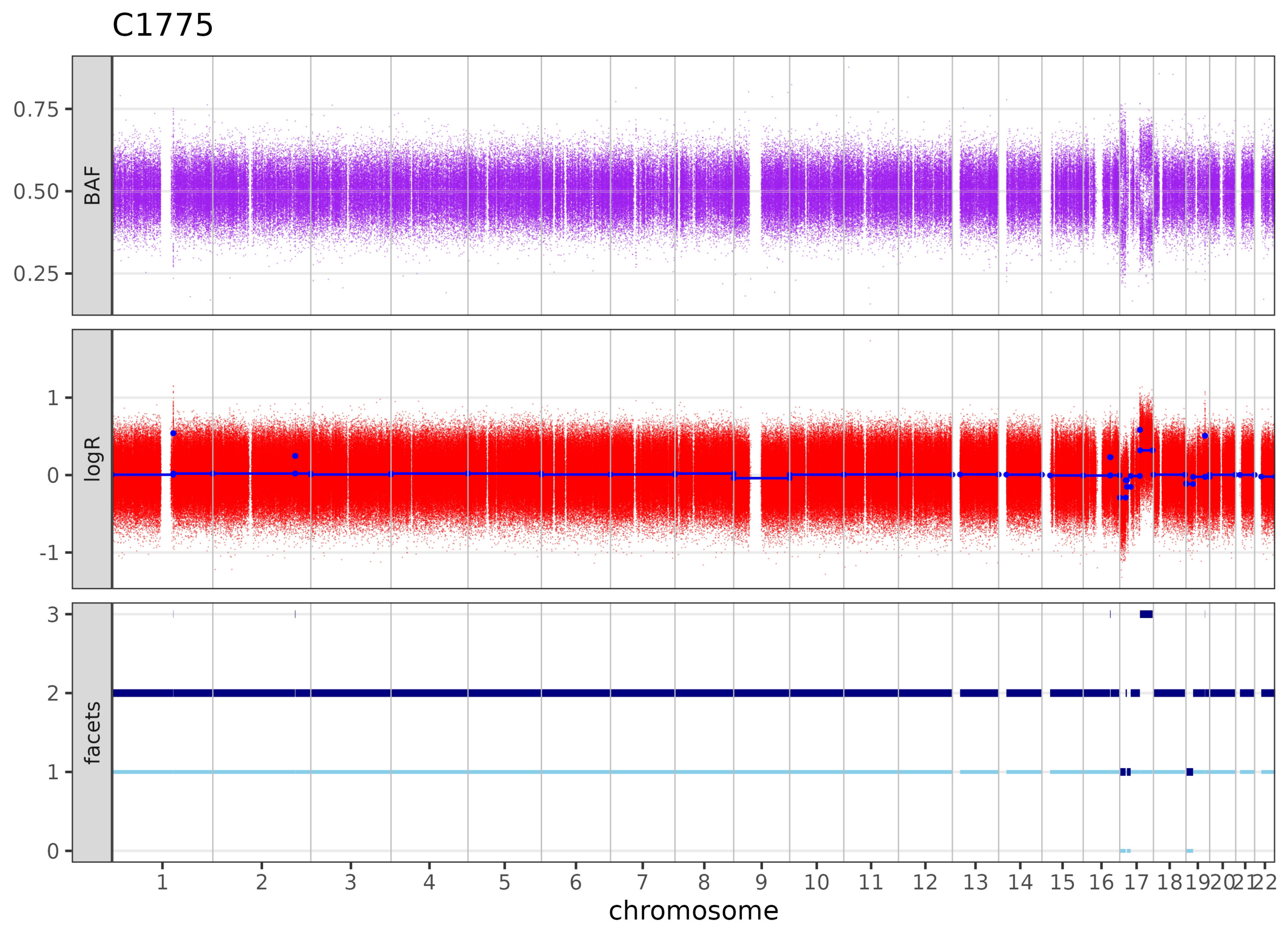

C1775
BAF
0.75
0.50
0.25
logR
1
0
-1
facets
3
2
1
0
1 2 3 4 5 6 7 8 9 10 11 12 13 14 15 16 17 18 19 20 21 22
chromosome

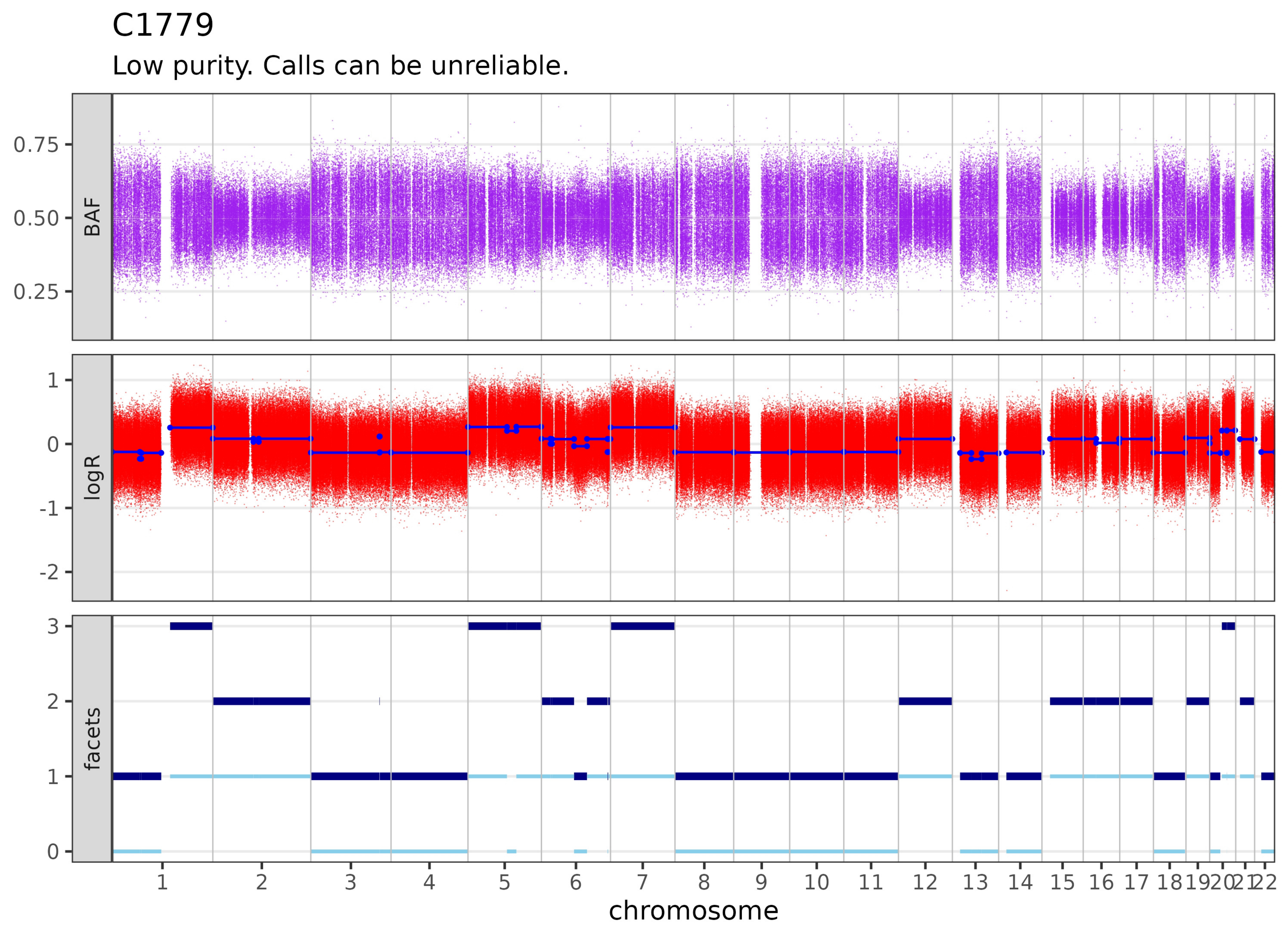
C1779
Low purity. Calls can be unreliable.
BAF
logR
facets
0.75
0.50
0.25
1
0
-1
-2
3
2
1
0
1 2 3 4 5 6 7 8 9 10 11 12 13 14 15 16 17 18 19 20 21 22
chromosome

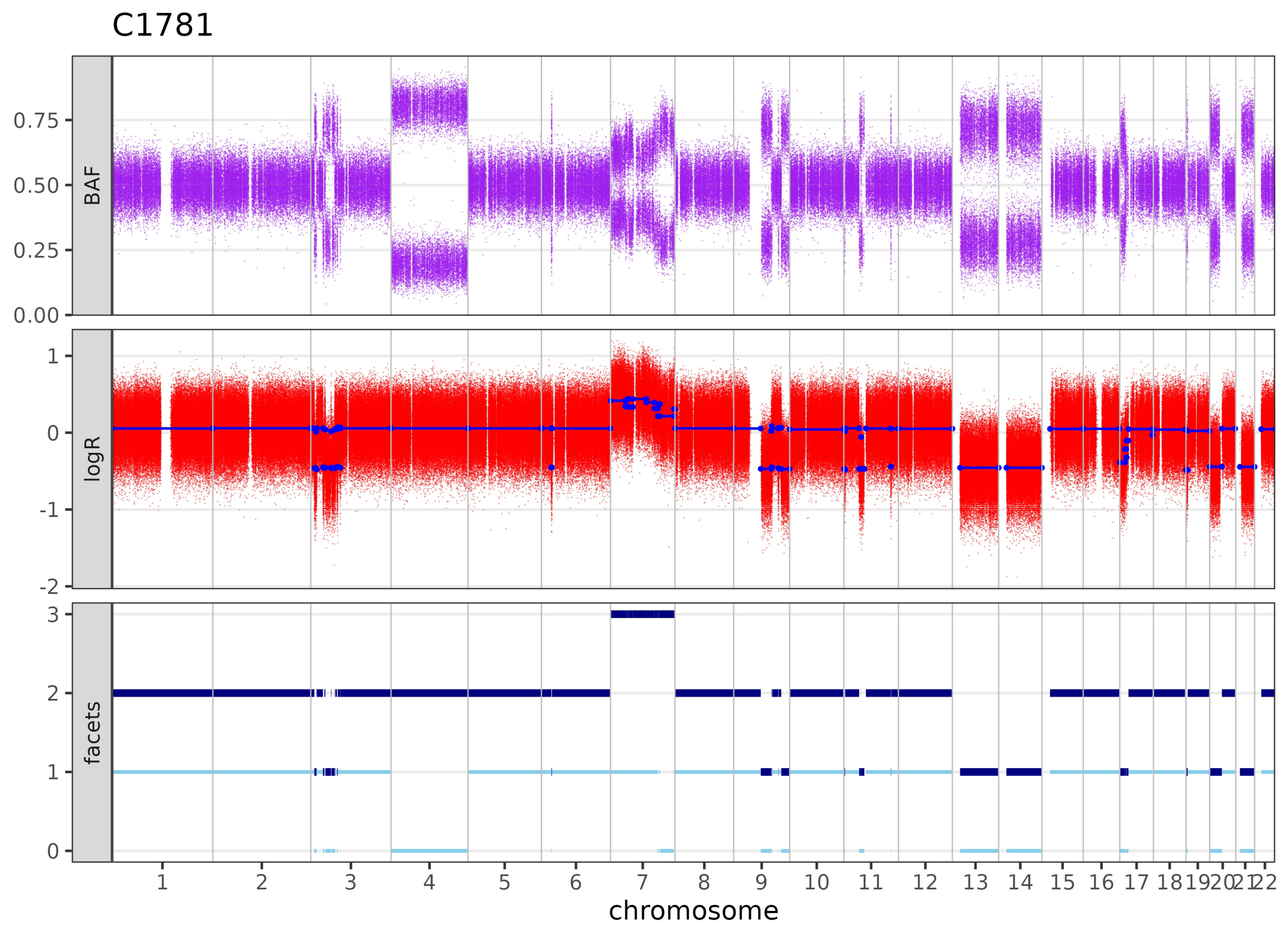
C1781
BAF
0.75
0.50
0.25
0.00
logR
1
0
-1
-2
facets
3
2
1
0
1
2
3
4
5
6
7
8
9
10
11
12
13
14
15
16
17
18
19
20
21
22
chromosome

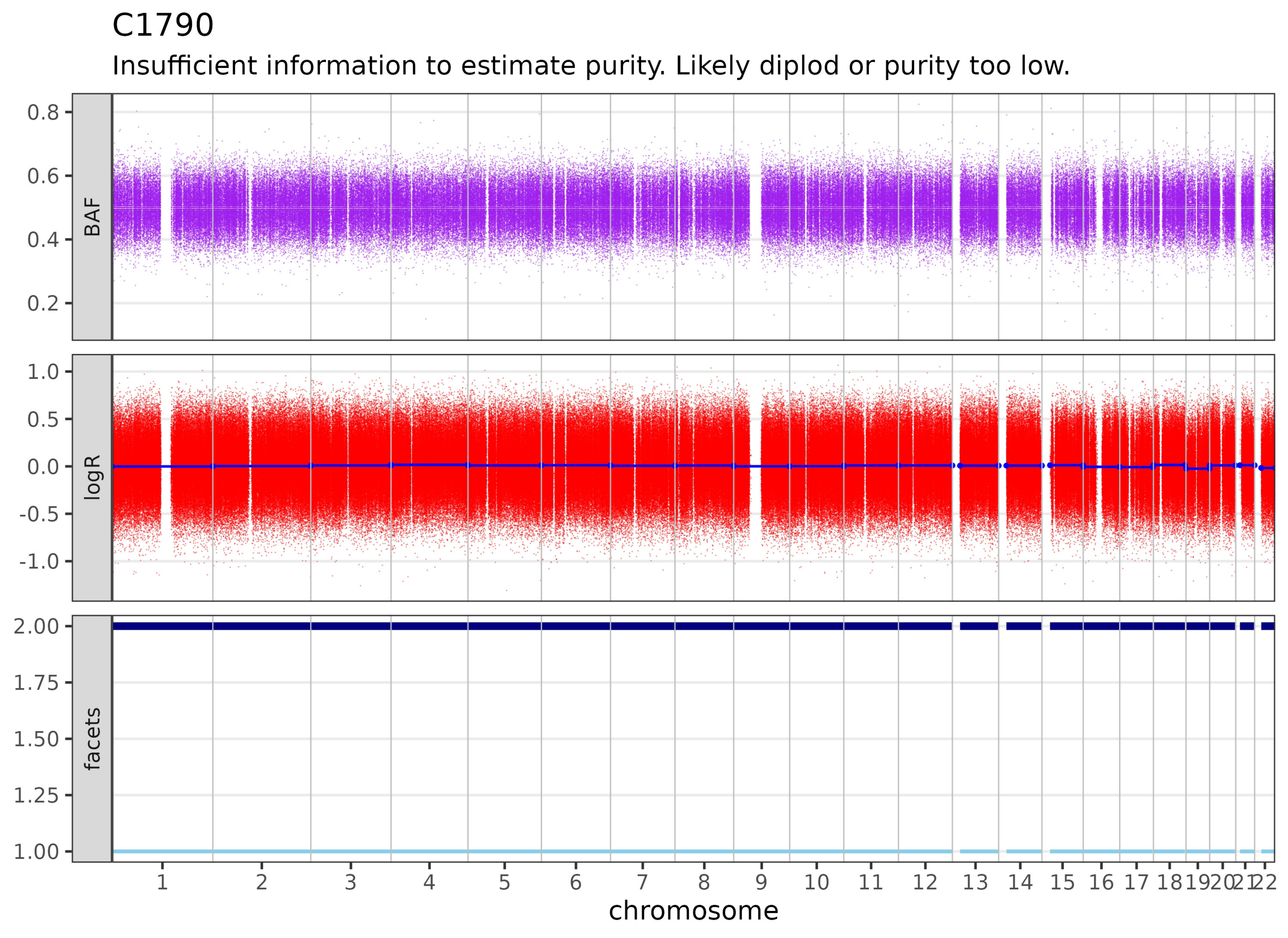
C1790
Insufficient information to estimate purity. Likely diplod or purity too low.
BAF
0.8
0.6
0.4
0.2
logR
1.0
0.5
0.0
-0.5
-1.0
facets
2.00
1.75
1.50
1.25
1.00
1 2 3 4 5 6 7 8 9 10 11 12 13 14 15 16 17 18 19 20 21 22
chromosome

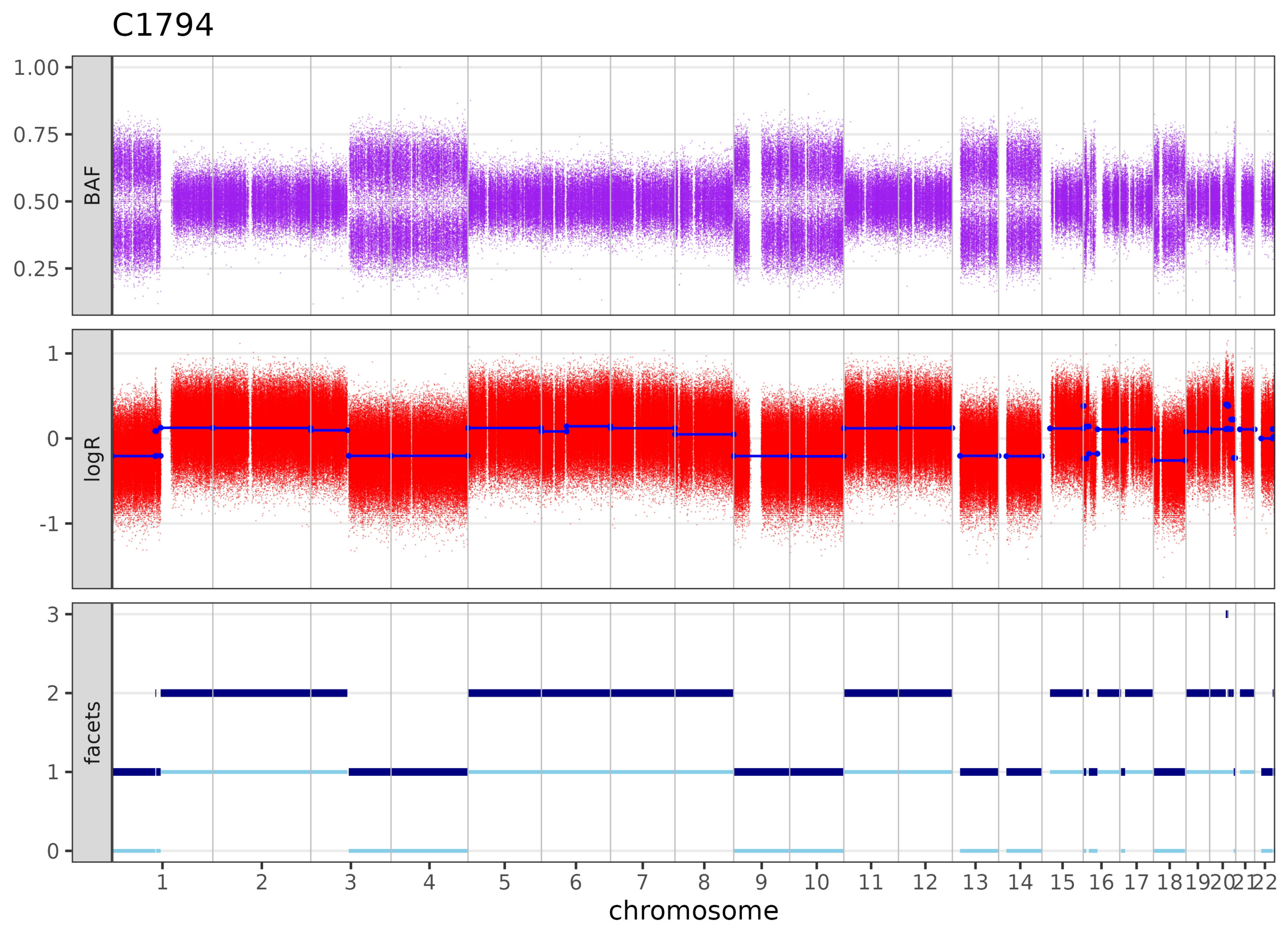

C1794
BAF
1.00
0.75
0.50
0.25
logR
1
0
-1
facets
3
2
1
0
1 2 3 4 5 6 7 8 9 10 11 12 13 14 15 16 17 18 19 20 21 22
chromosome

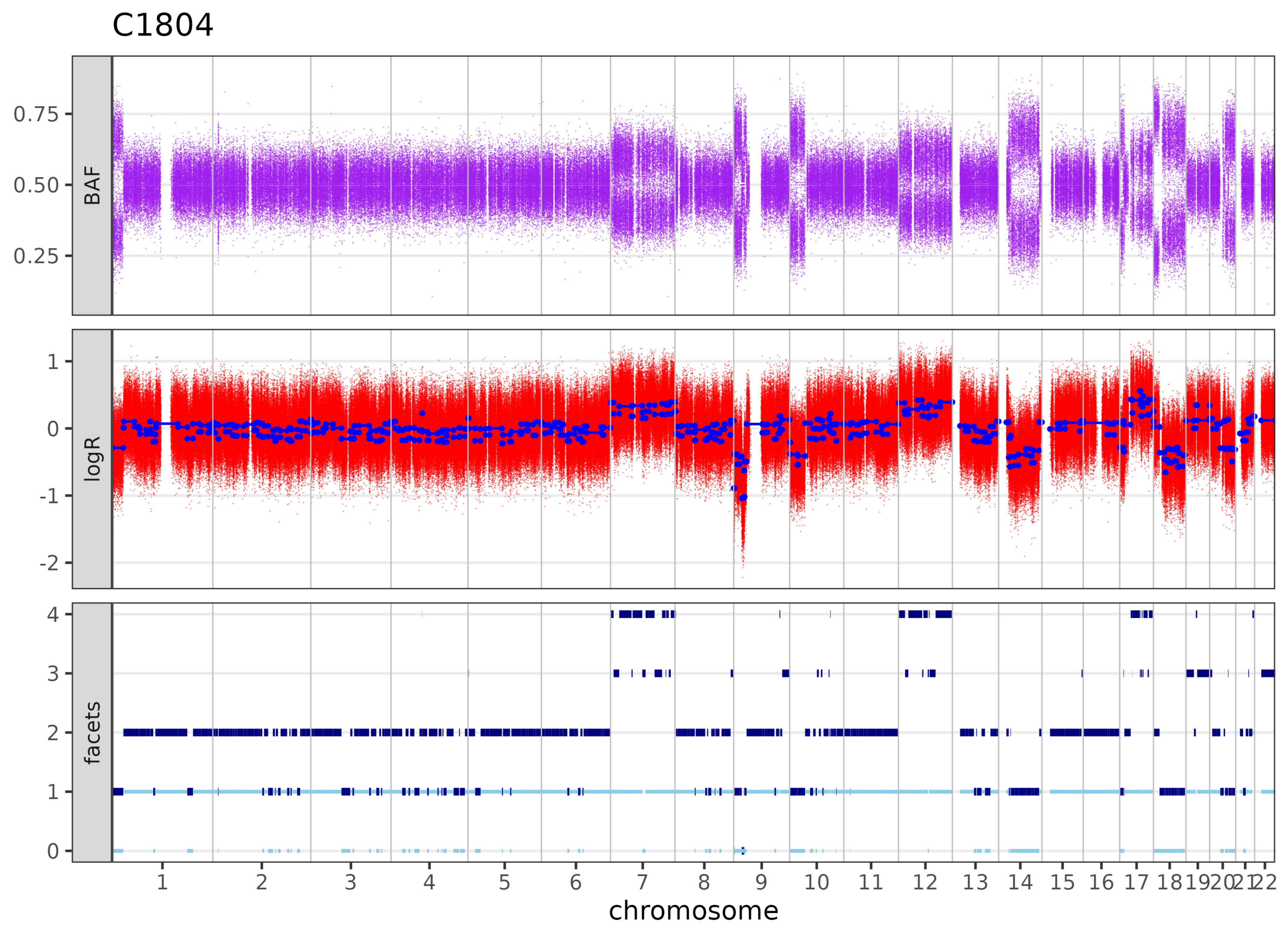
C1804
BAF
0.75
0.50
0.25
logR
1
0
-1
-2
facets
4
3
2
1
0
1
2
3
4
5
6
7
8
9
10
11
12
13
14
15
16
17
18
19
20
21
22
chromosome

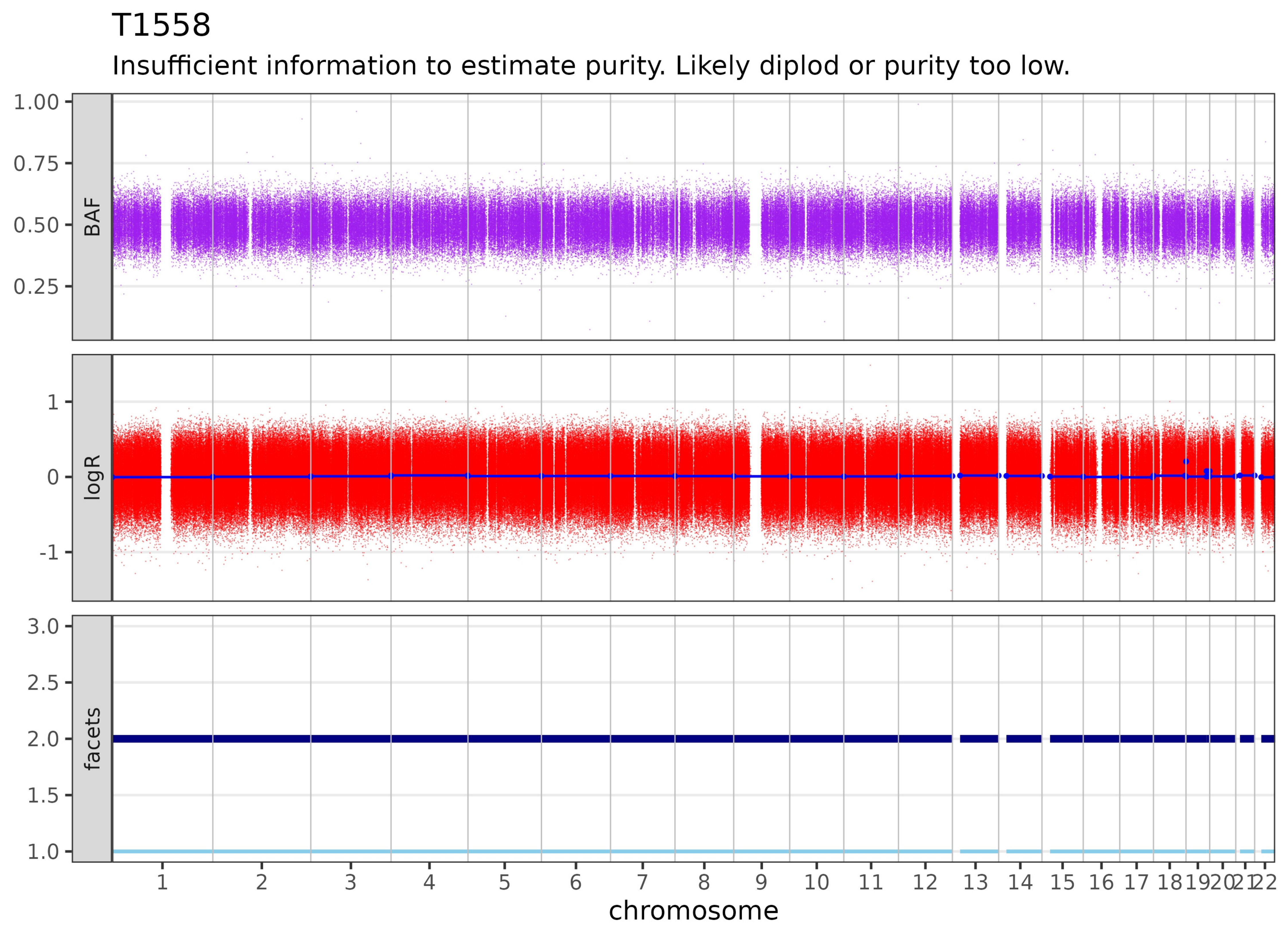
T1558
Insufficient information to estimate purity. Likely diplod or purity too low.
BAF
logR
facets
1.00
0.75
0.50
0.25
1
0
-1
3.0
2.5
2.0
1.5
1.0
1 2 3 4 5 6 7 8 9 10 11 12 13 14 15 16 17 18 19 20 21 22
chromosome

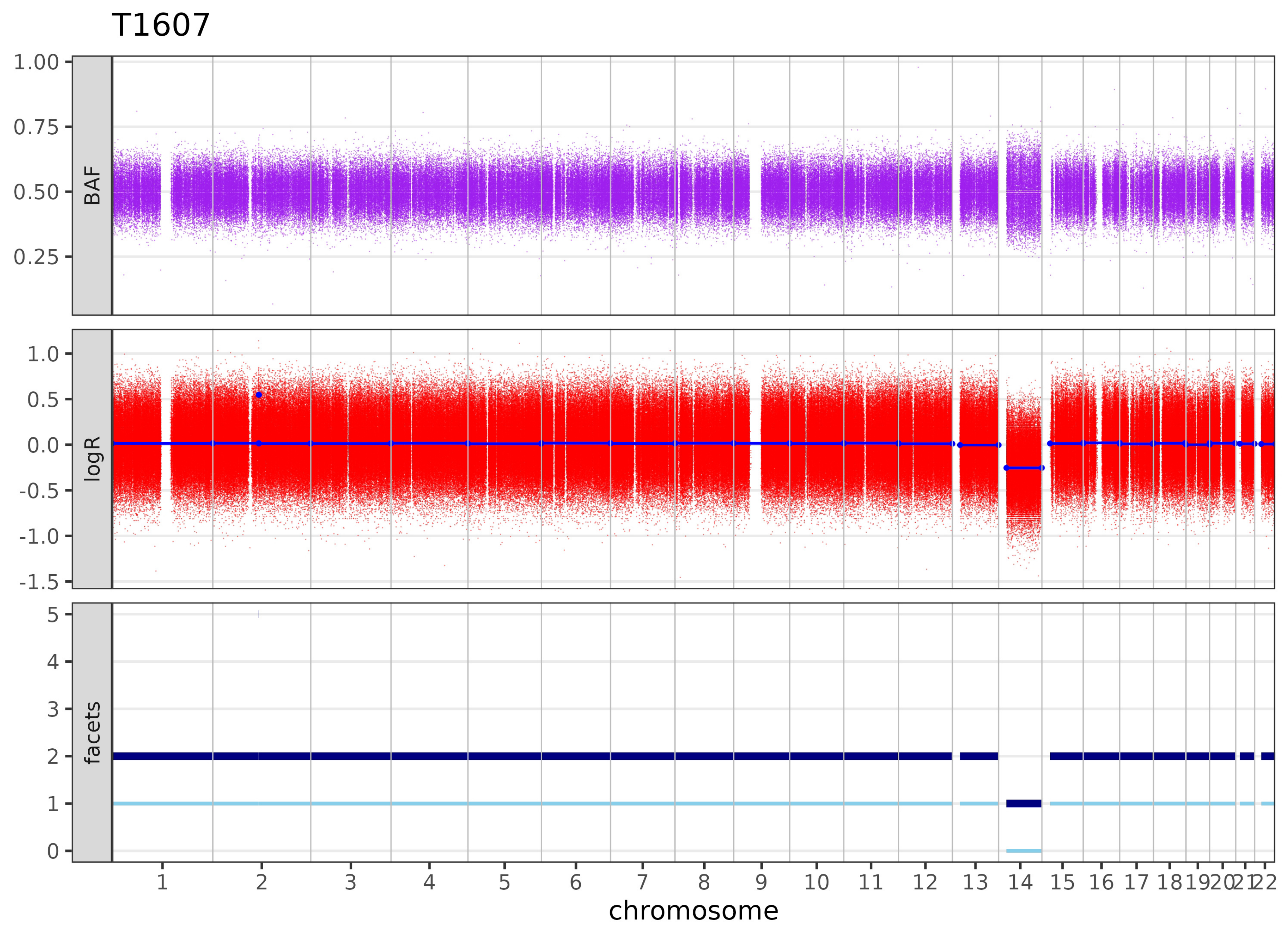

T1607
BAF
logR
facets
1.00
0.75
0.50
0.25
1.0
0.5
0.0
-0.5
-1.0
-1.5
5
4
3
2
1
0
1 2 3 4 5 6 7 8 9 10 11 12 13 14 15 16 17 18 19 20 21 22
chromosome

T1641

Low purity. Calls can be unreliable.

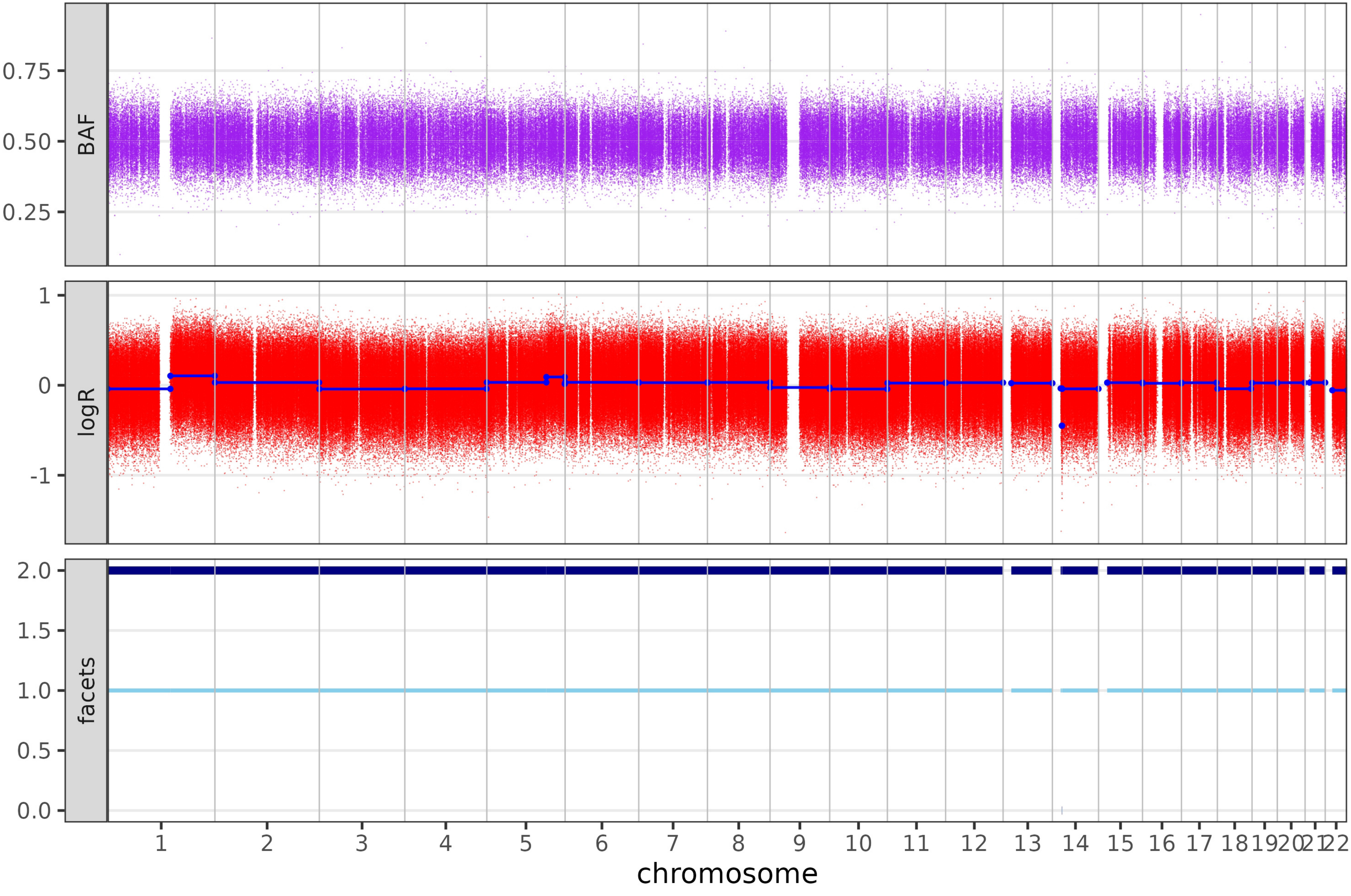

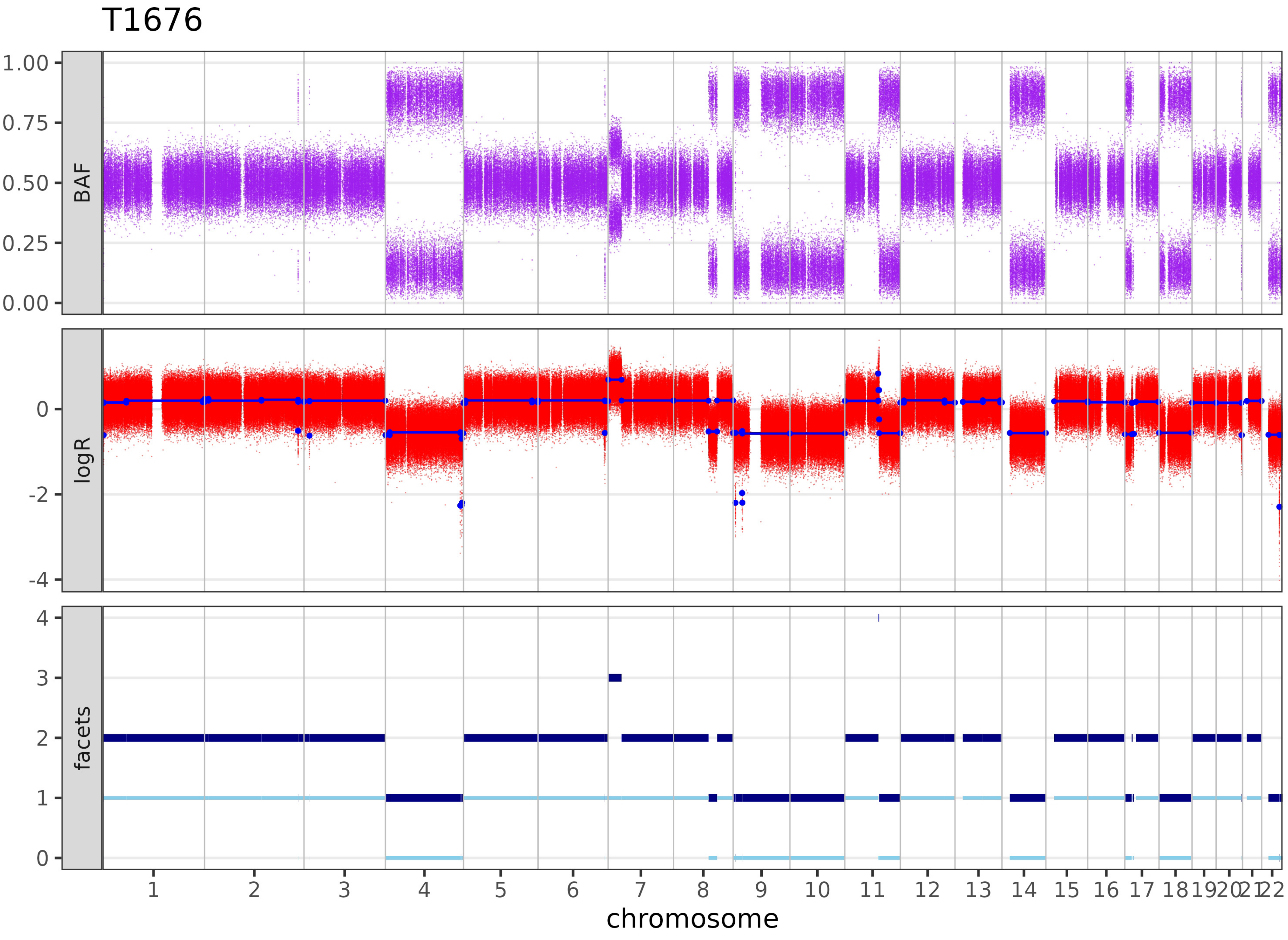

T1676
BAF
logR
facets
1.00
0.75
0.50
0.25
0.00
0
-2
-4
4
3
2
1
0
1 2 3 4 5 6 7 8 9 10 11 12 13 14 15 16 17 18 19 20 21 22
chromosome

T1706

Insufficient information to estimate purity. Likely diplod or purity too low.

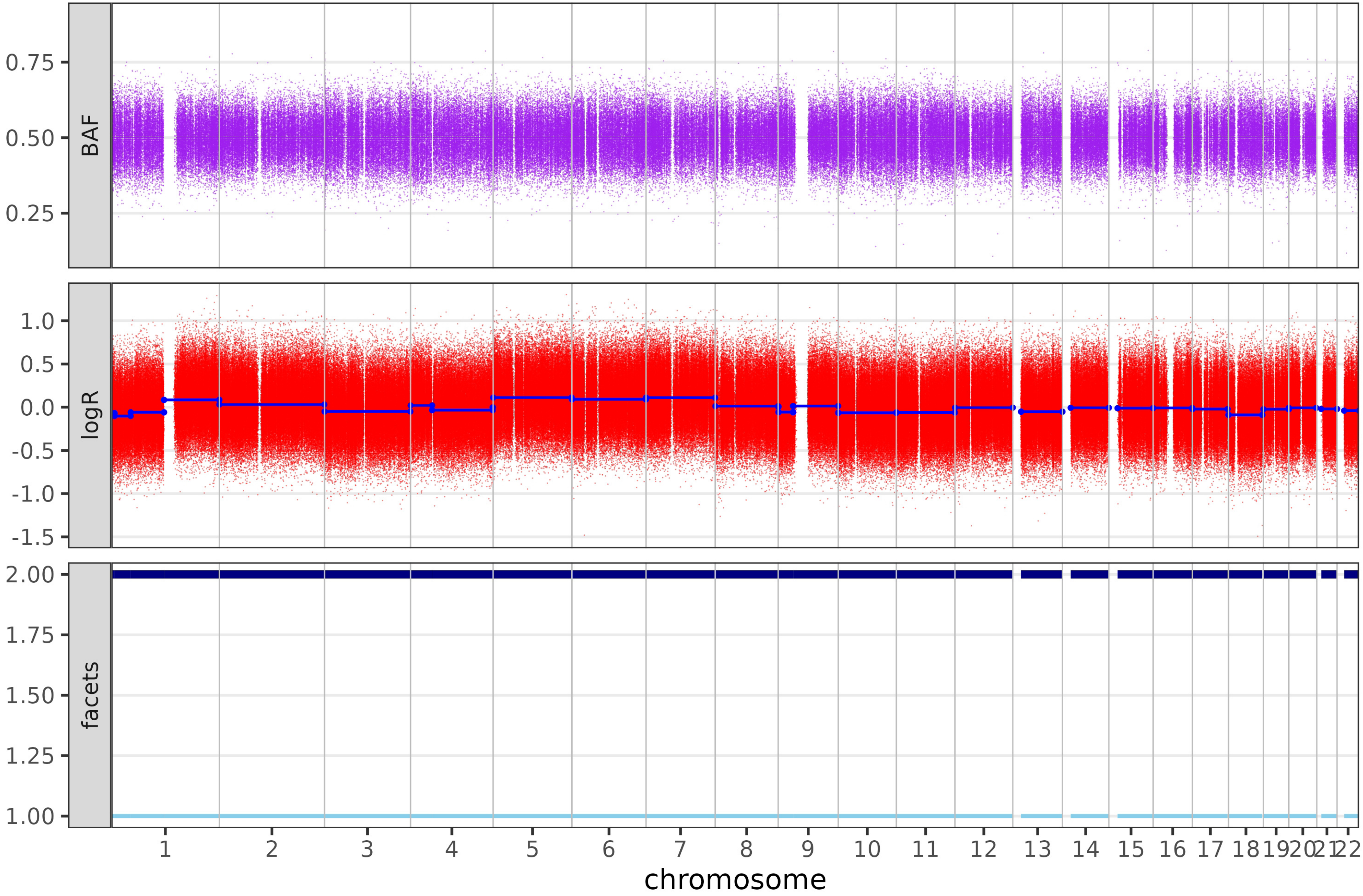

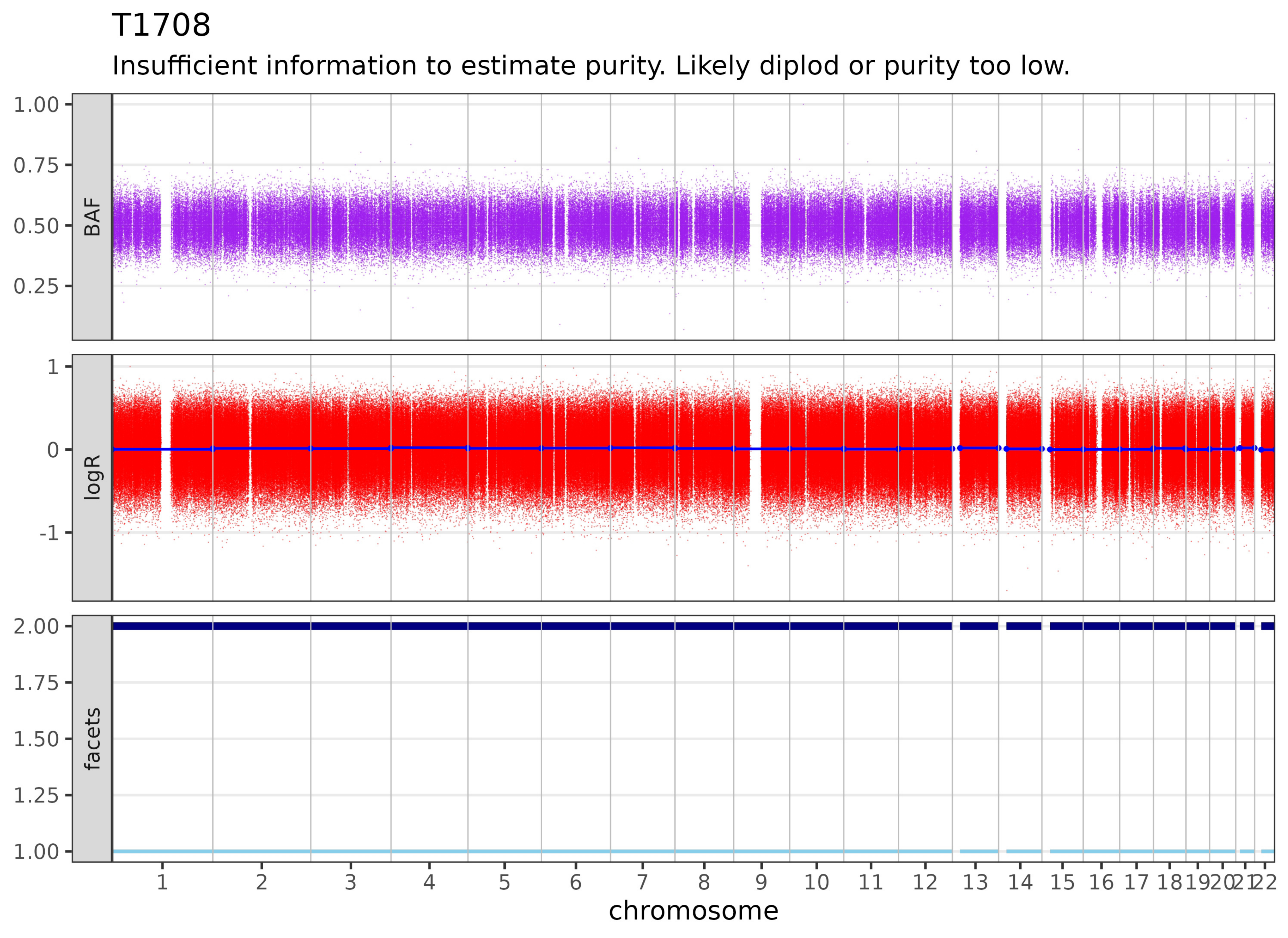
T1708
Insufficient information to estimate purity. Likely diplod or purity too low.
BAF
logR
facets
1.00
0.75
0.50
0.25
1
0
-1
2.00
1.75
1.50
1.25
1.00
1 2 3 4 5 6 7 8 9 10 11 12 13 14 15 16 17 18 19 20 21 22
chromosome

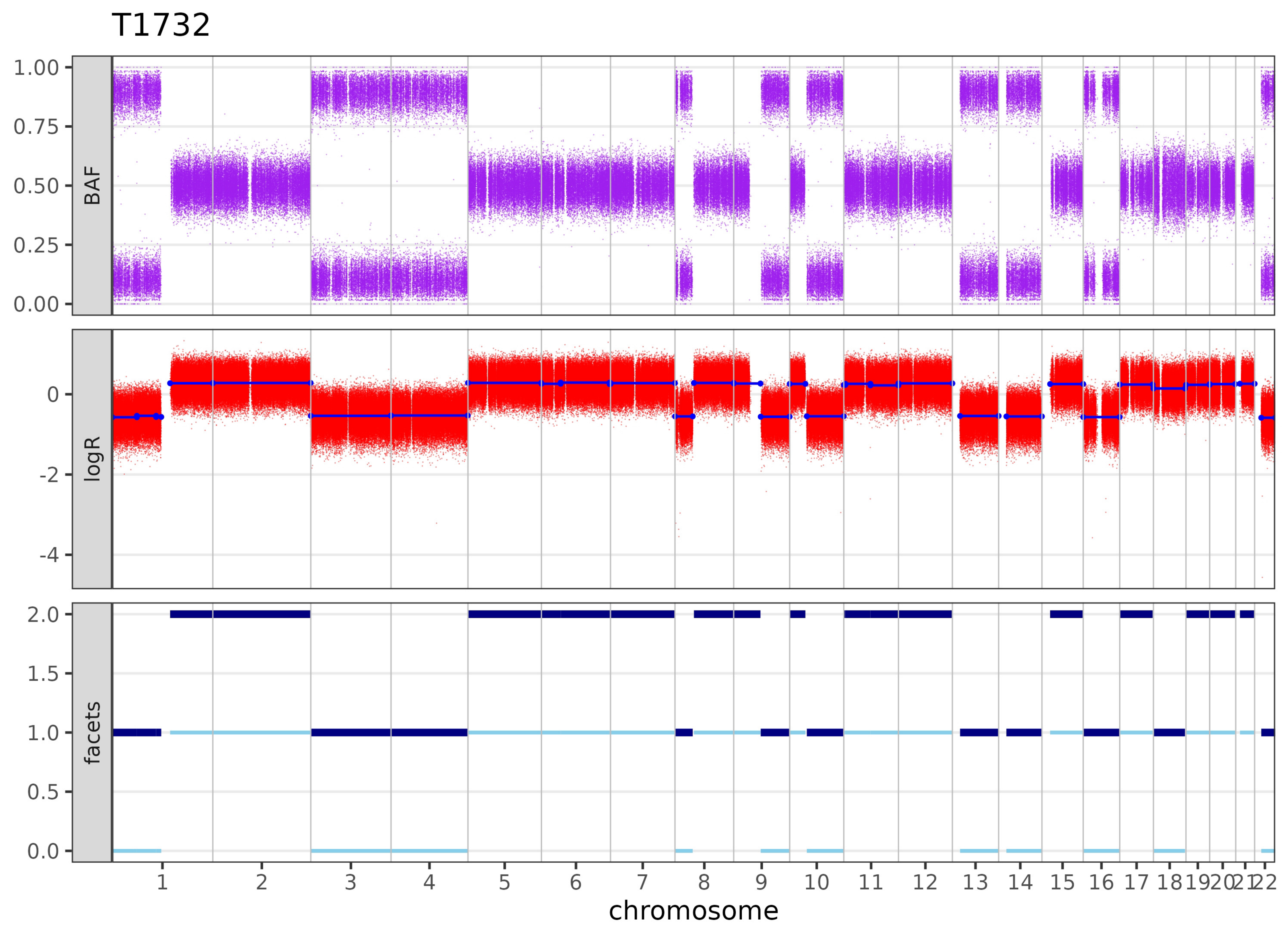

T1732
BAF
logR
facets
1.00
0.75
0.50
0.25
0.00
0
-2
-4
2.0
1.5
1.0
0.5
0.0
1 2 3 4 5 6 7 8 9 10 11 12 13 14 15 16 17 18 19 20 21 22
chromosome

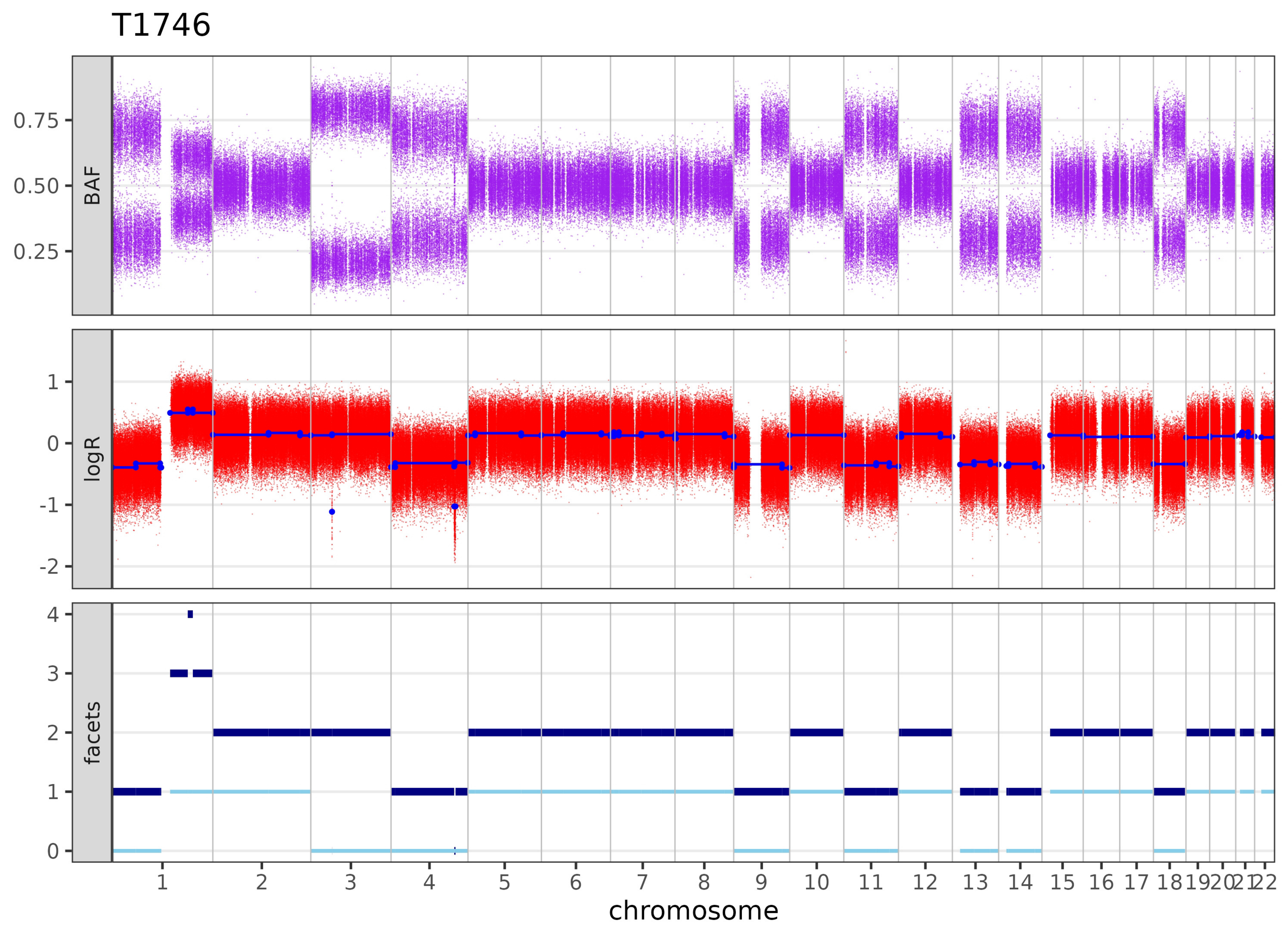

T1746
BAF
0.75
0.50
0.25
logR
1
0
-1
-2
facets
4
3
2
1
0
1
2
3
4
5
6
7
8
9
10
11
12
13
14
15
16
17
18
19
20
21
22
chromosome

T1813

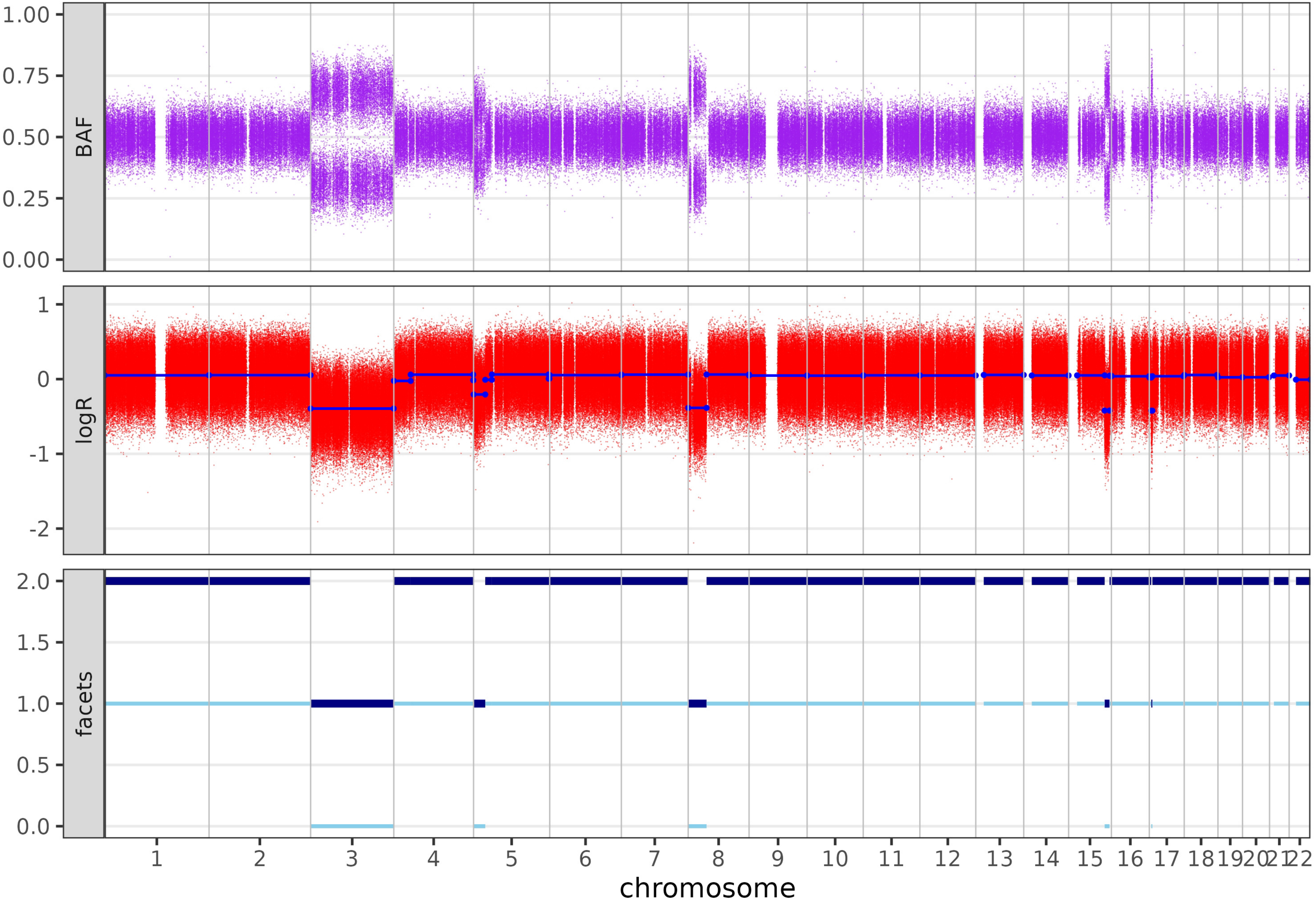
BAF
logR
facets
1.00
0.75
0.50
0.25
0.00
1
0
-1
-2
2.0
1.5
1.0
0.5
0.0
1 2 3 4 5 6 7 8 9 10 11 12 13 14 15 16 17 18 19 20 21 22
chromosome

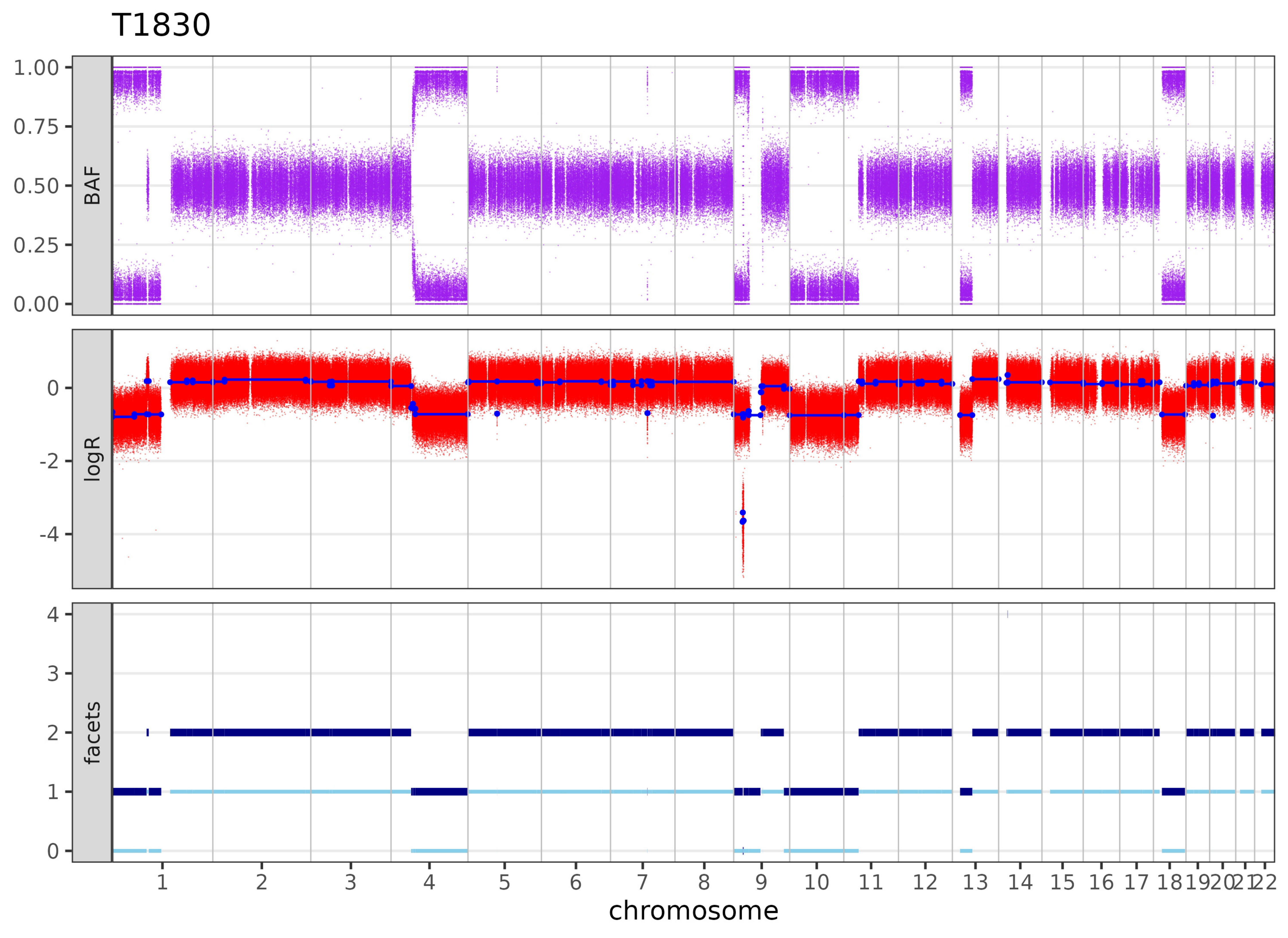
T1830
BAF
logR
facets
chromosome
1.00
0.75
0.50
0.25
0.00
0
-2
-4
4
3
2
1
0
1 2 3 4 5 6 7 8 9 10 11 12 13 14 15 16 17 18 19 20 21 22

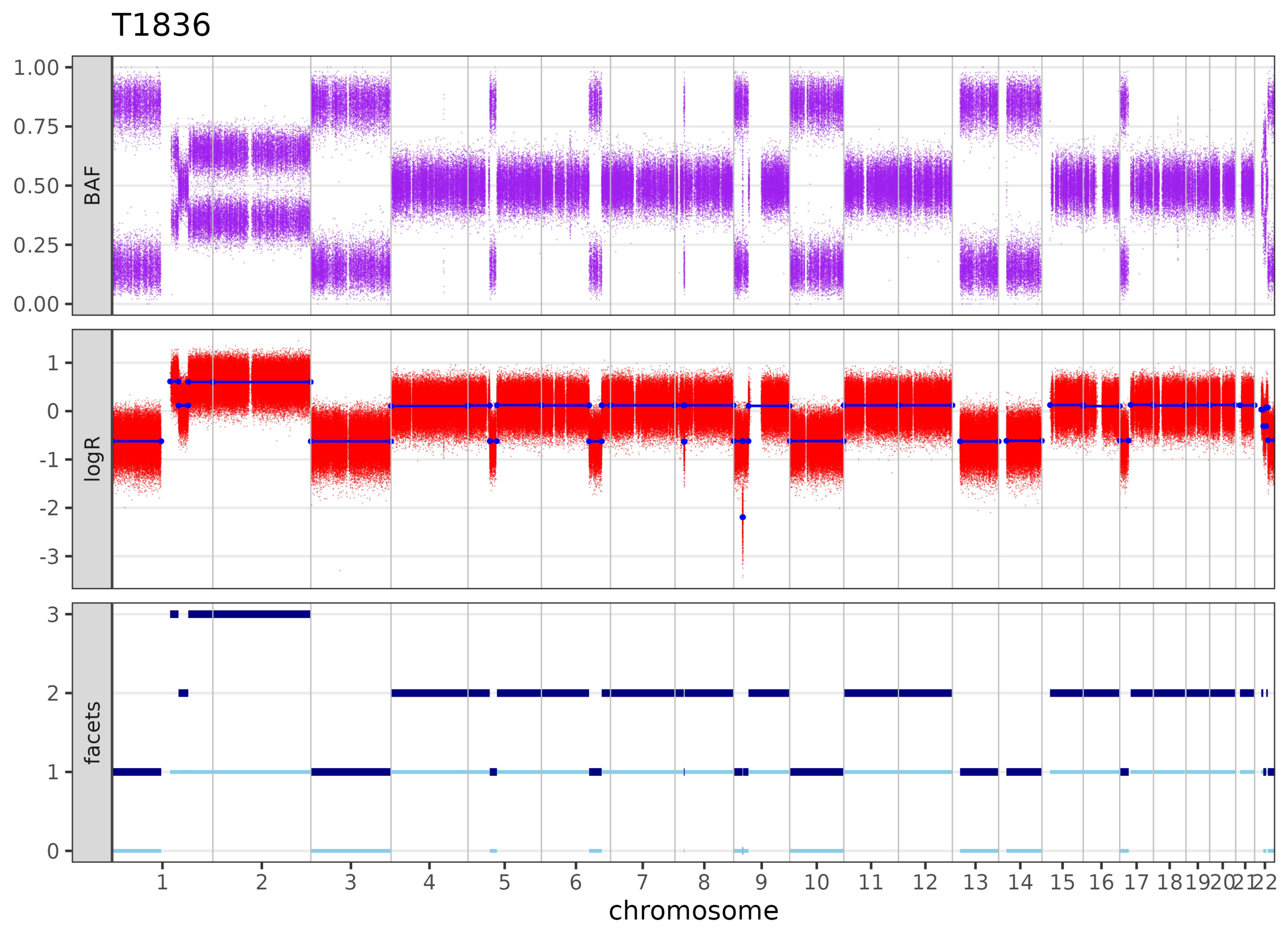
T1836
BAF
1.00
0.75
0.50
0.25
0.00
logR
1
0
-1
-2
-3
facets
3
2
1
0
1 2 3 4 5 6 7 8 9 10 11 12 13 14 15 16 17 18 19 20 21 22
chromosome

T1844

Low purity. Calls can be unreliable.

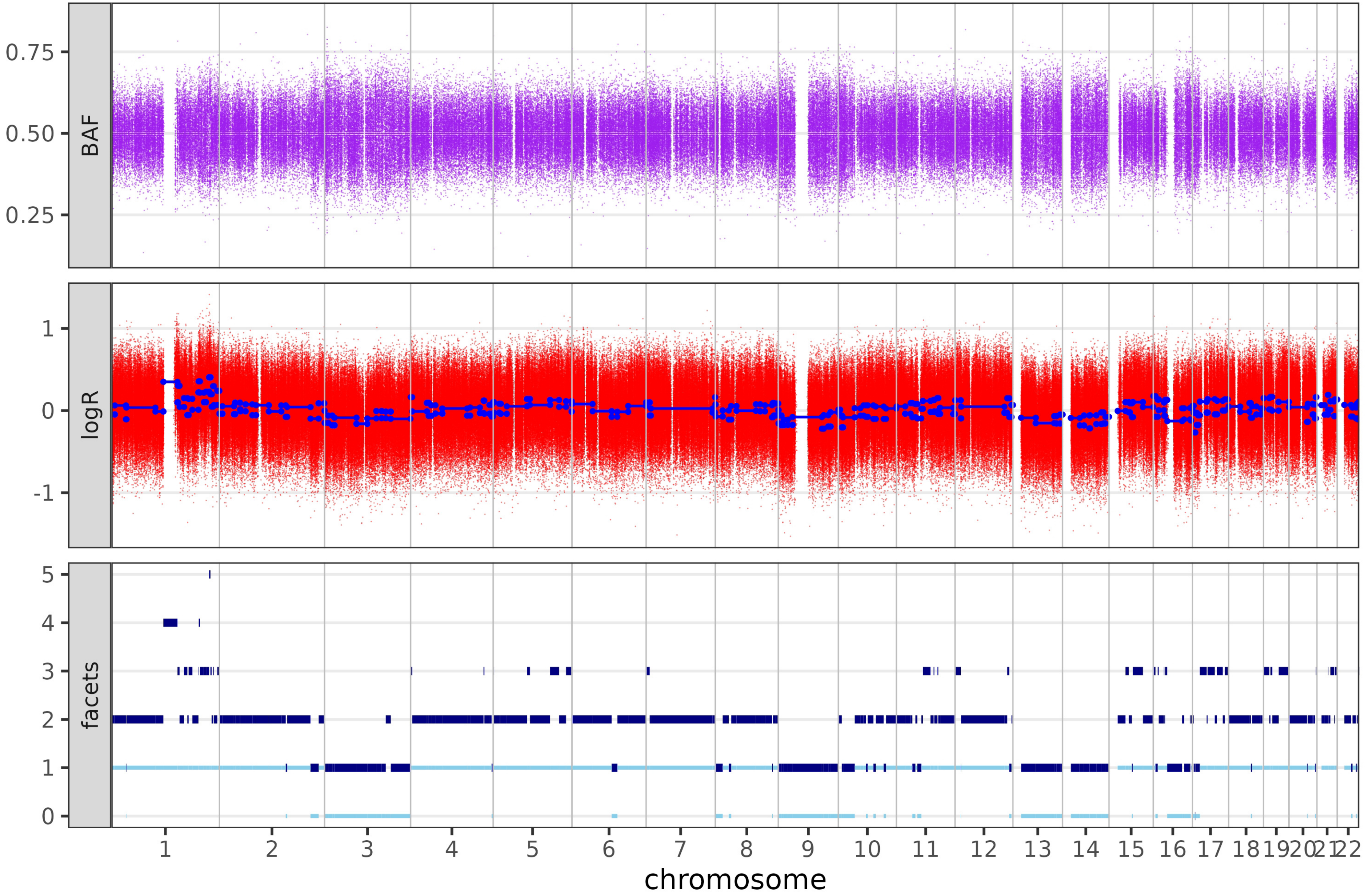

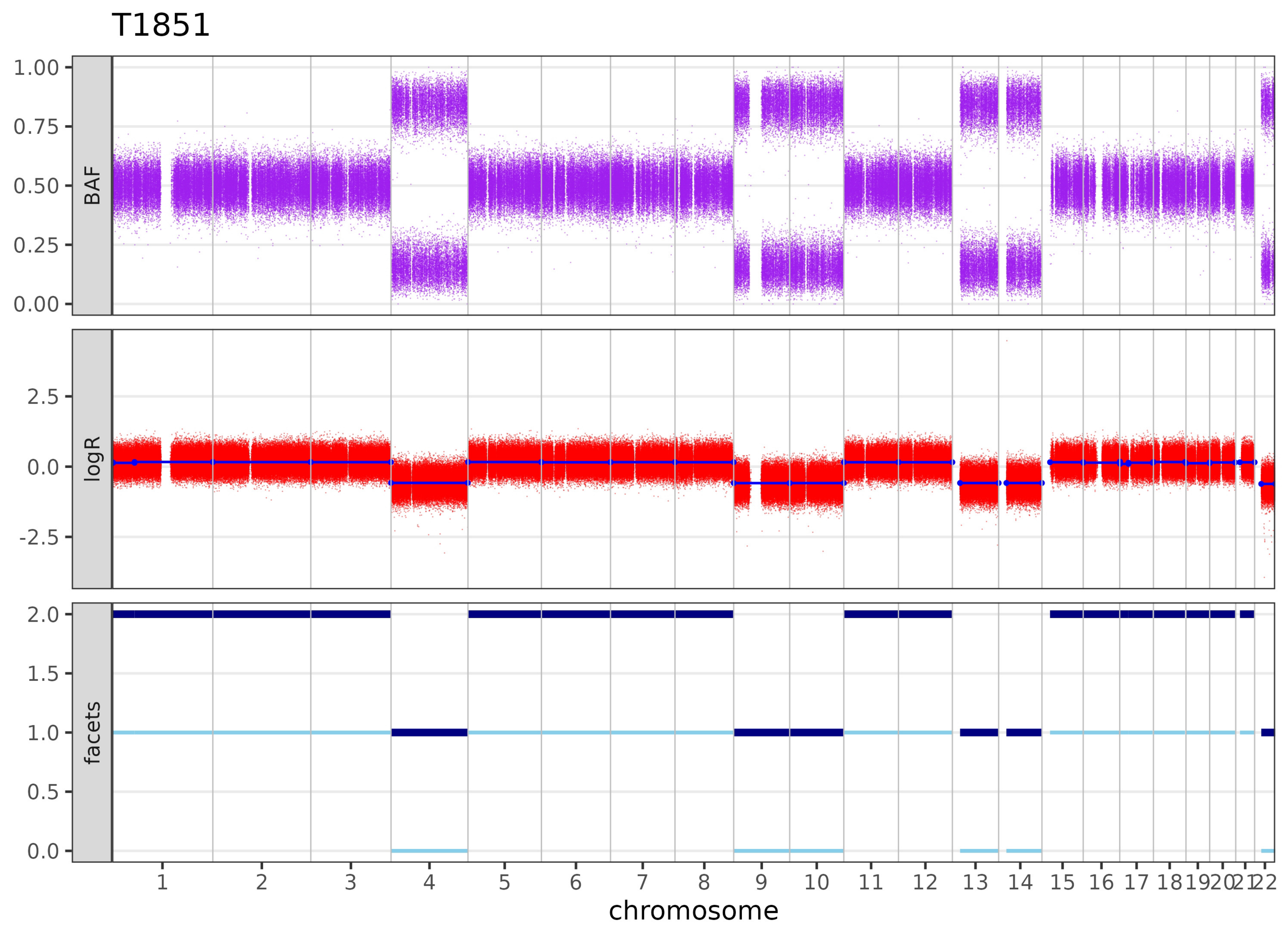
T1851
BAF
logR
facets
1.00
0.75
0.50
0.25
0.00
2.5
0.0
-2.5
2.0
1.5
1.0
0.5
0.0
1 2 3 4 5 6 7 8 9 10 11 12 13 14 15 16 17 18 19 20 21 22
chromosome

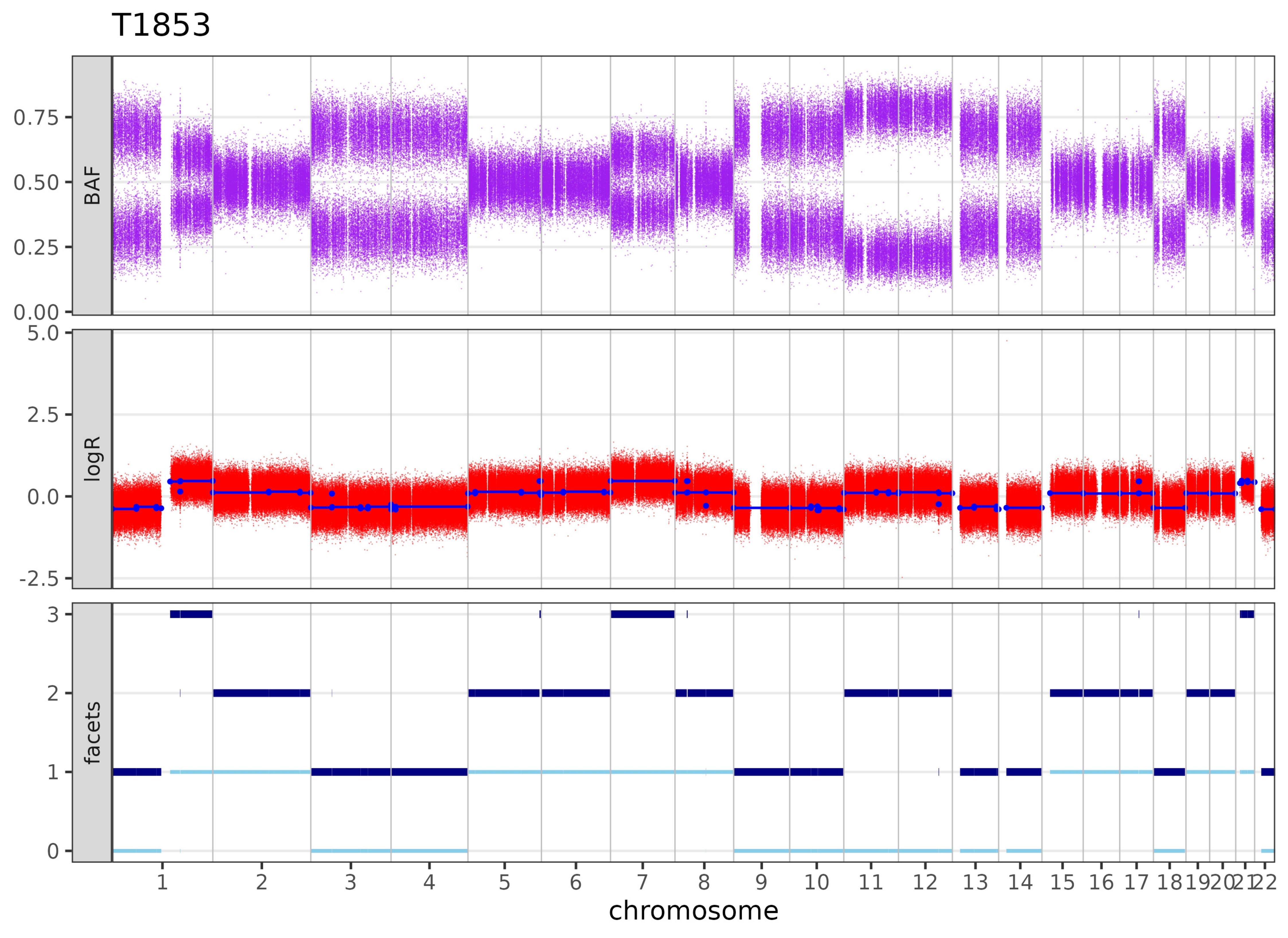
T1853
BAF
0.75
0.50
0.25
0.00
logR
5.0
2.5
0.0
-2.5
facets
3
2
1
0
1
2
3
4
5
6
7
8
9
10
11
12
13
14
15
16
17
18
19
20
21
22
chromosome

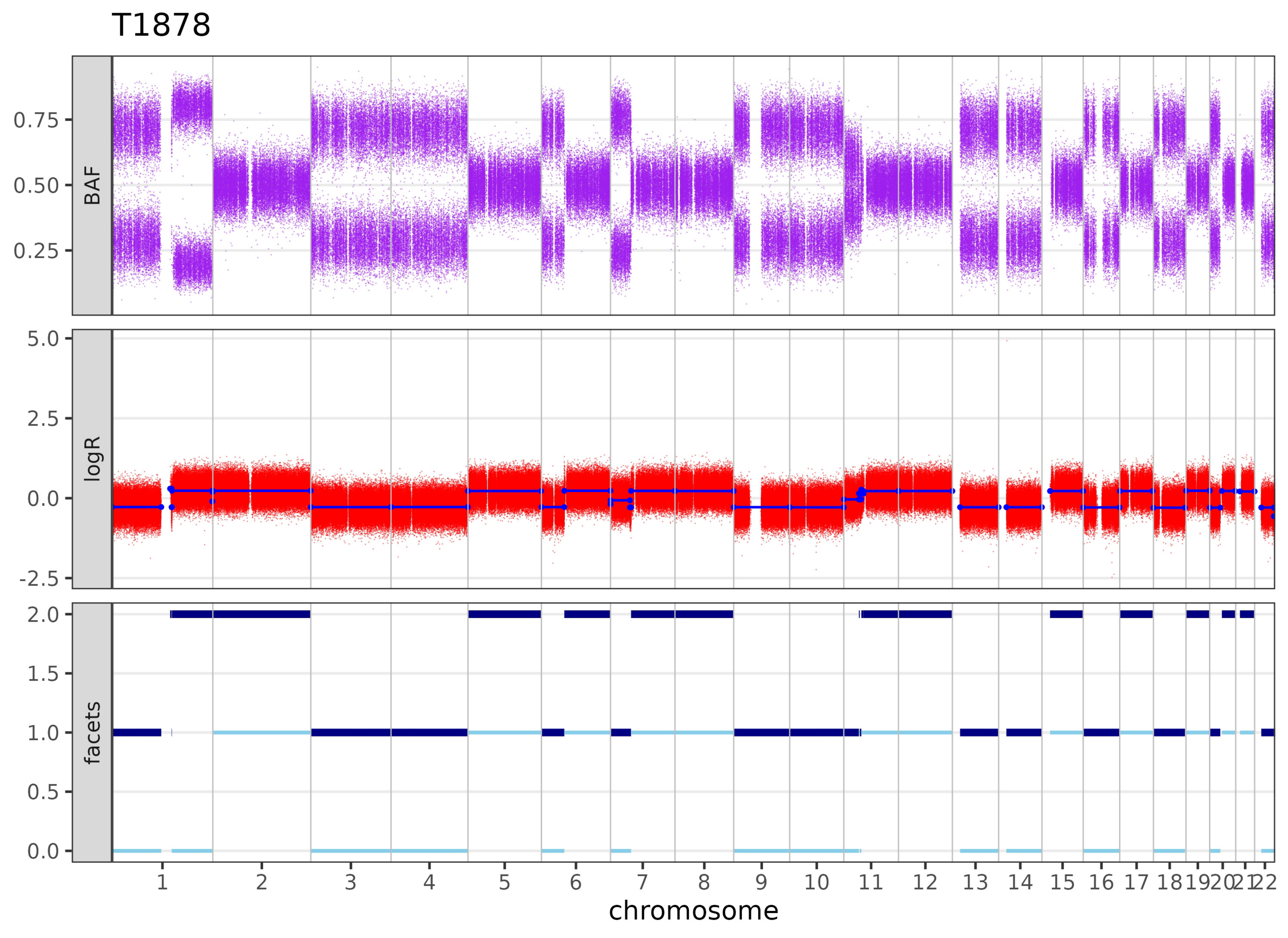
T1878
BAF
0.75
0.50
0.25
logR
5.0
2.5
0.0
-2.5
facets
2.0
1.5
1.0
0.5
0.0
1 2 3 4 5 6 7 8 9 10 11 12 13 14 15 16 17 18 19 20 21 22
chromosome

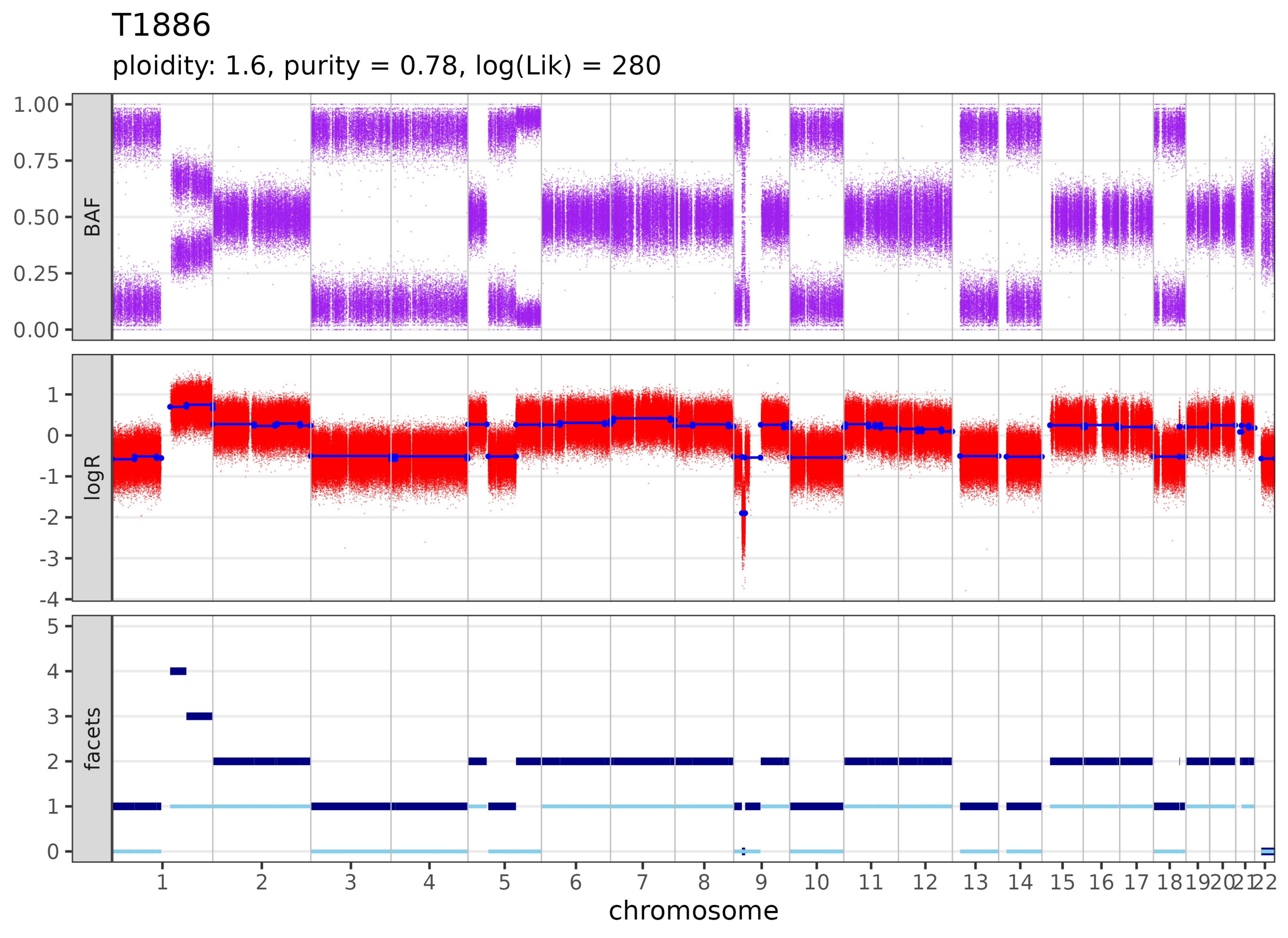

T1886
ploidity: 1.6, purity = 0.78, log(Lik) = 280
BAF
1.00
0.75
0.50
0.25
0.00
logR
1
0
-1
-2
-3
-4
facets
5
4
3
2
1
0
1 2 3 4 5 6 7 8 9 10 11 12 13 14 15 16 17 18 19 20 21 22
chromosome

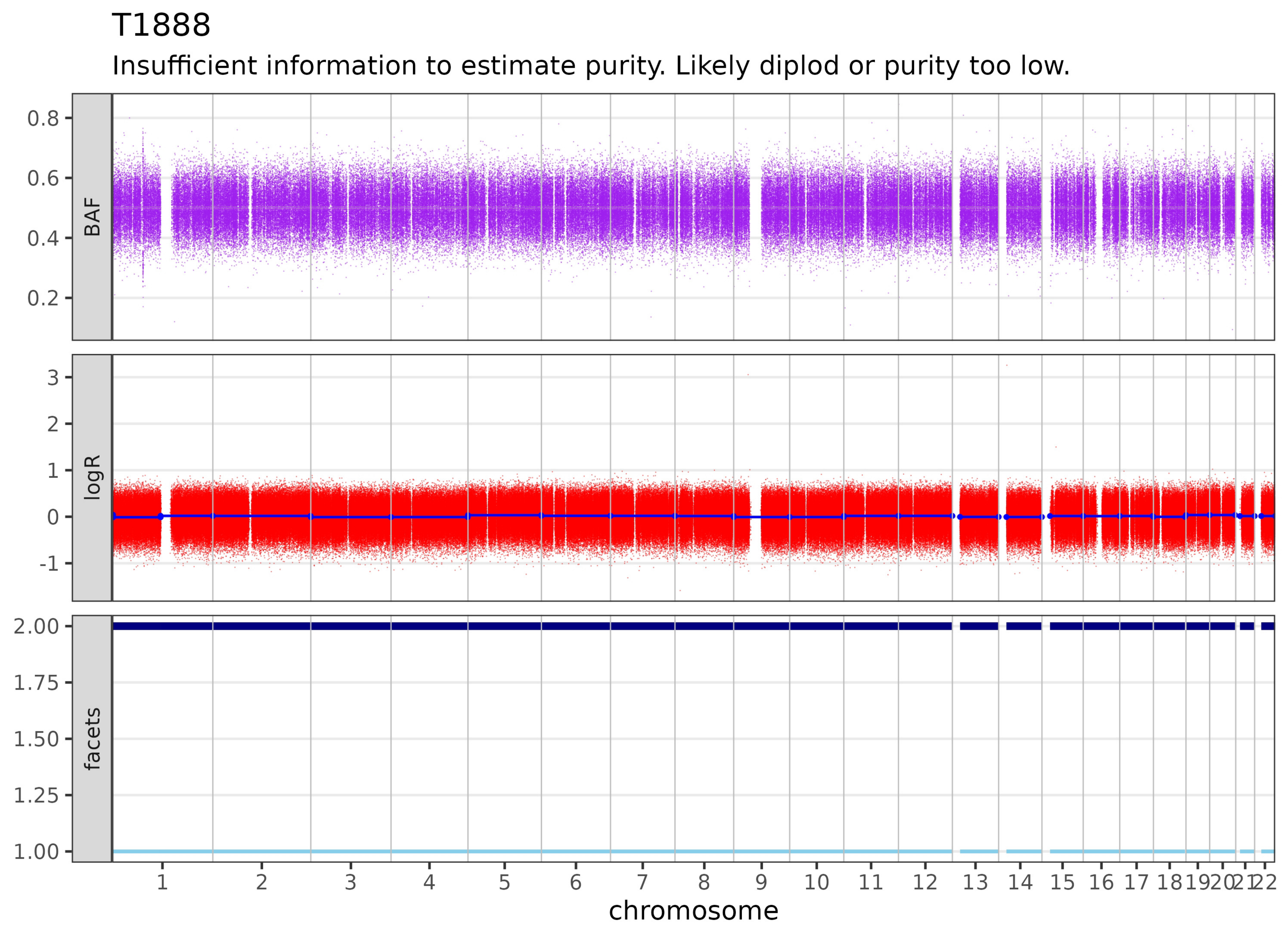
T1888
Insufficient information to estimate purity. Likely diplod or purity too low.
BAF
0.8
0.6
0.4
0.2
logR
3
2
1
0
-1
facets
2.00
1.75
1.50
1.25
1.00
1 2 3 4 5 6 7 8 9 10 11 12 13 14 15 16 17 18 19 20 21 22
chromosome

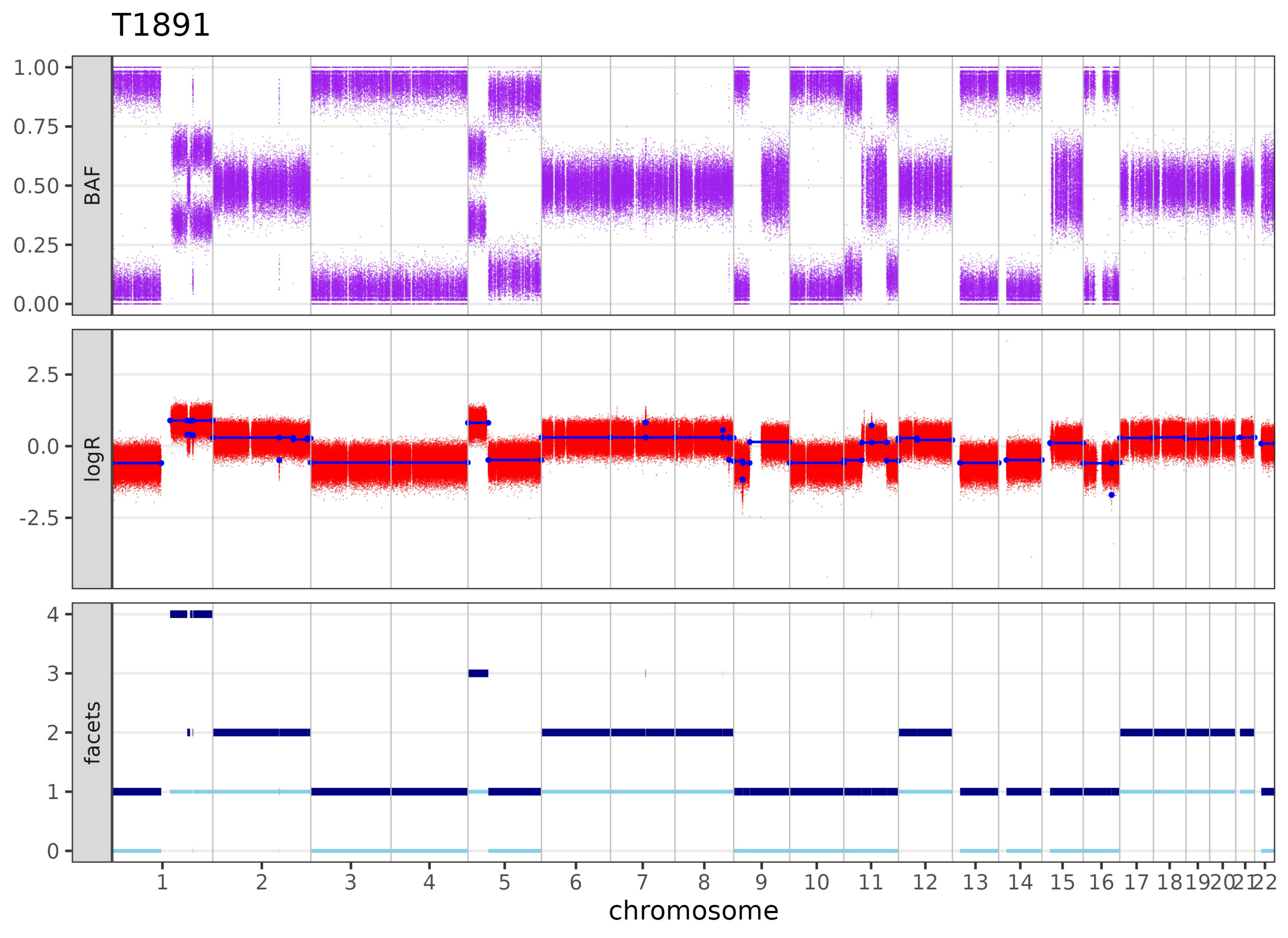

T1891
BAF
1.00
0.75
0.50
0.25
0.00
logR
2.5
0.0
-2.5
facets
4
3
2
1
0
1
2
3
4
5
6
7
8
9
10
11
12
13
14
15
16
17
18
19
20
21
22
chromosome

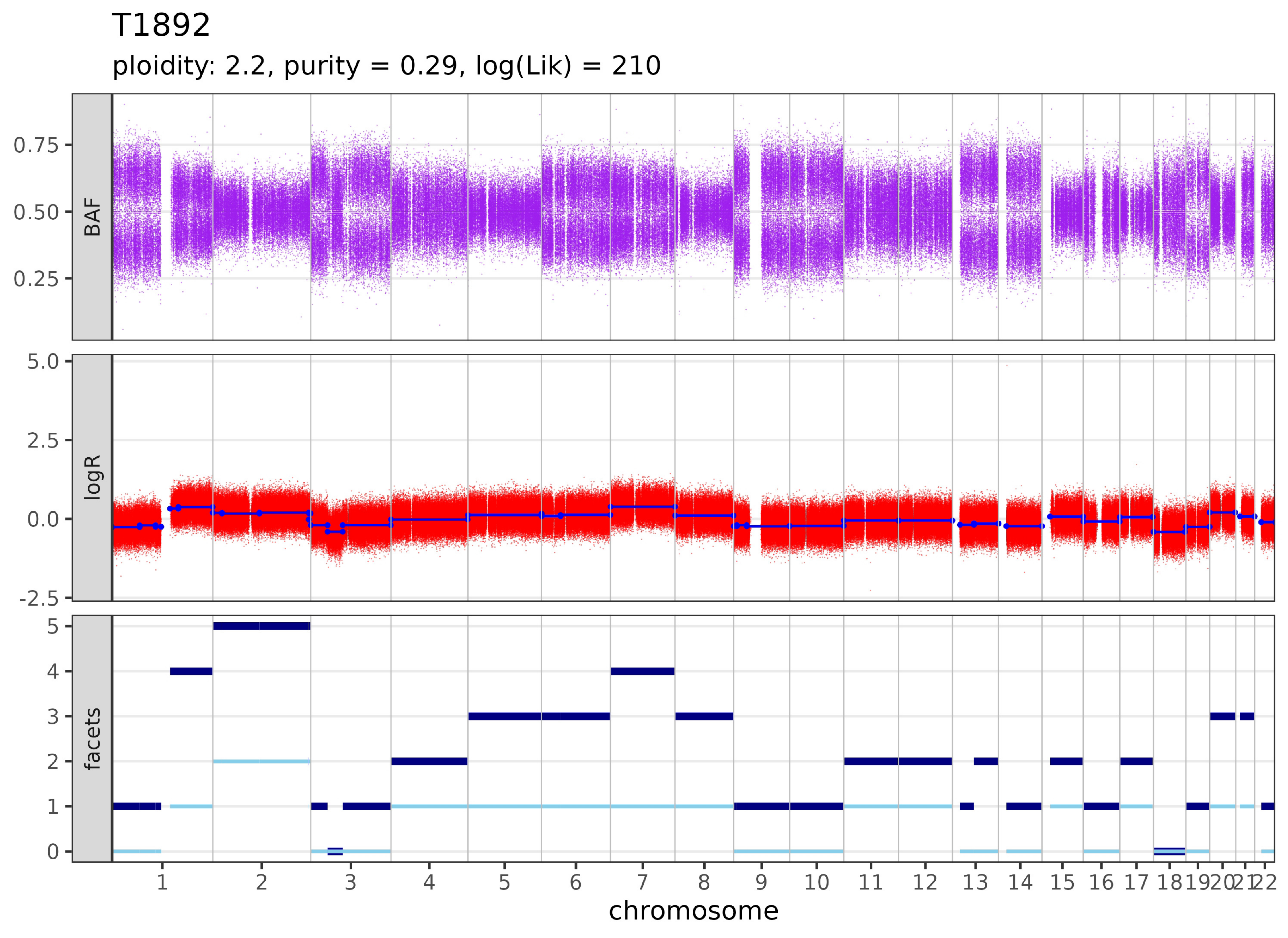
T1892
ploidity: 2.2, purity = 0.29, log(Lik) = 210
BAF
0.75
0.50
0.25
logR
5.0
2.5
0.0
-2.5
facets
5
4
3
2
1
0
1 2 3 4 5 6 7 8 9 10 11 12 13 14 15 16 17 18 19 20 21 22
chromosome

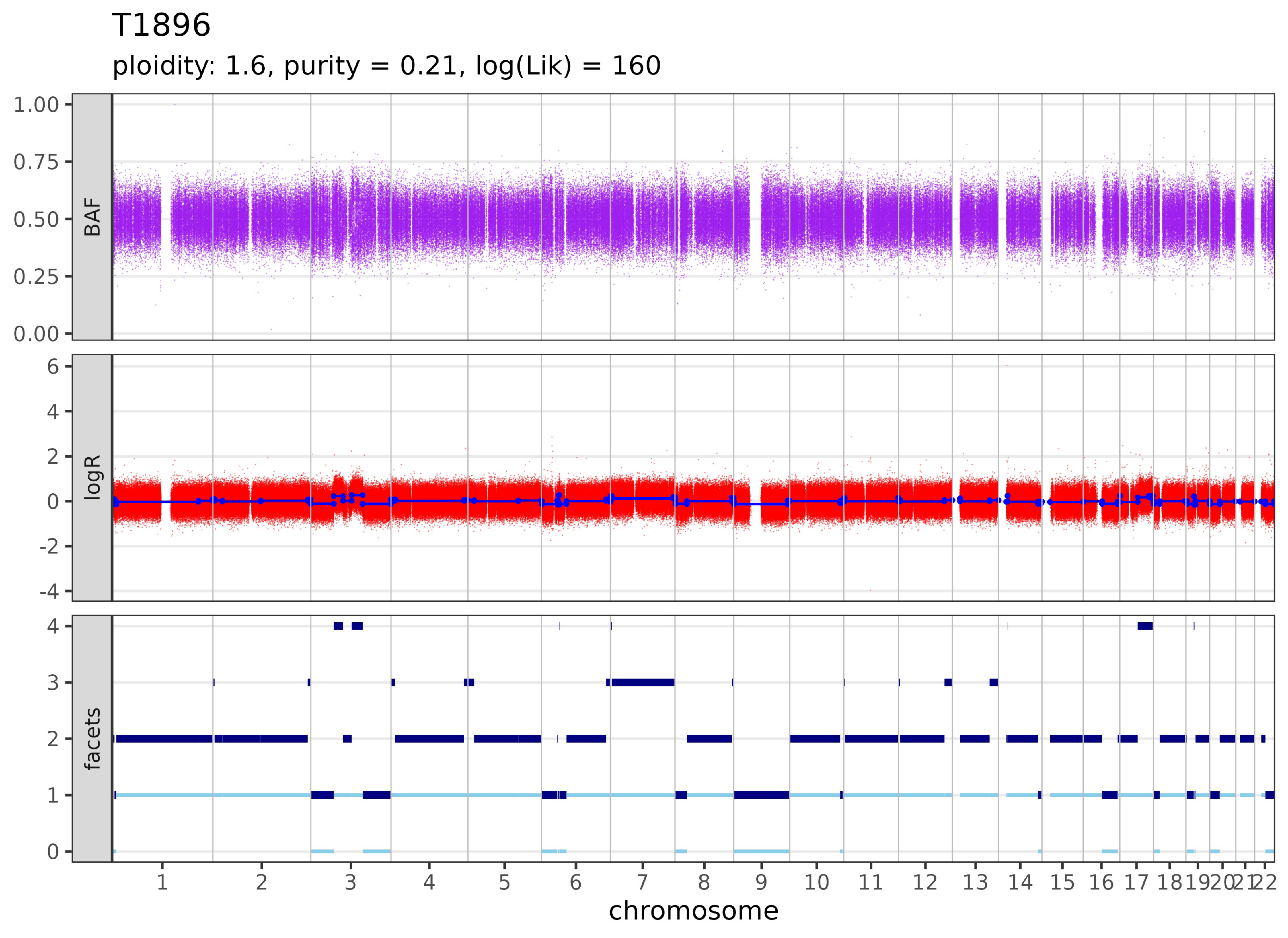
T1896
ploidity: 1.6, purity = 0.21, log(Lik) = 160
BAF
logR
facets
1.00
0.75
0.50
0.25
0.00
6
4
2
0
-2
-4
4
3
2
1
0
1
2
3
4
5
6
7
8
9
10
11
12
13
14
15
16
17
18
19
20
21
22
chromosome

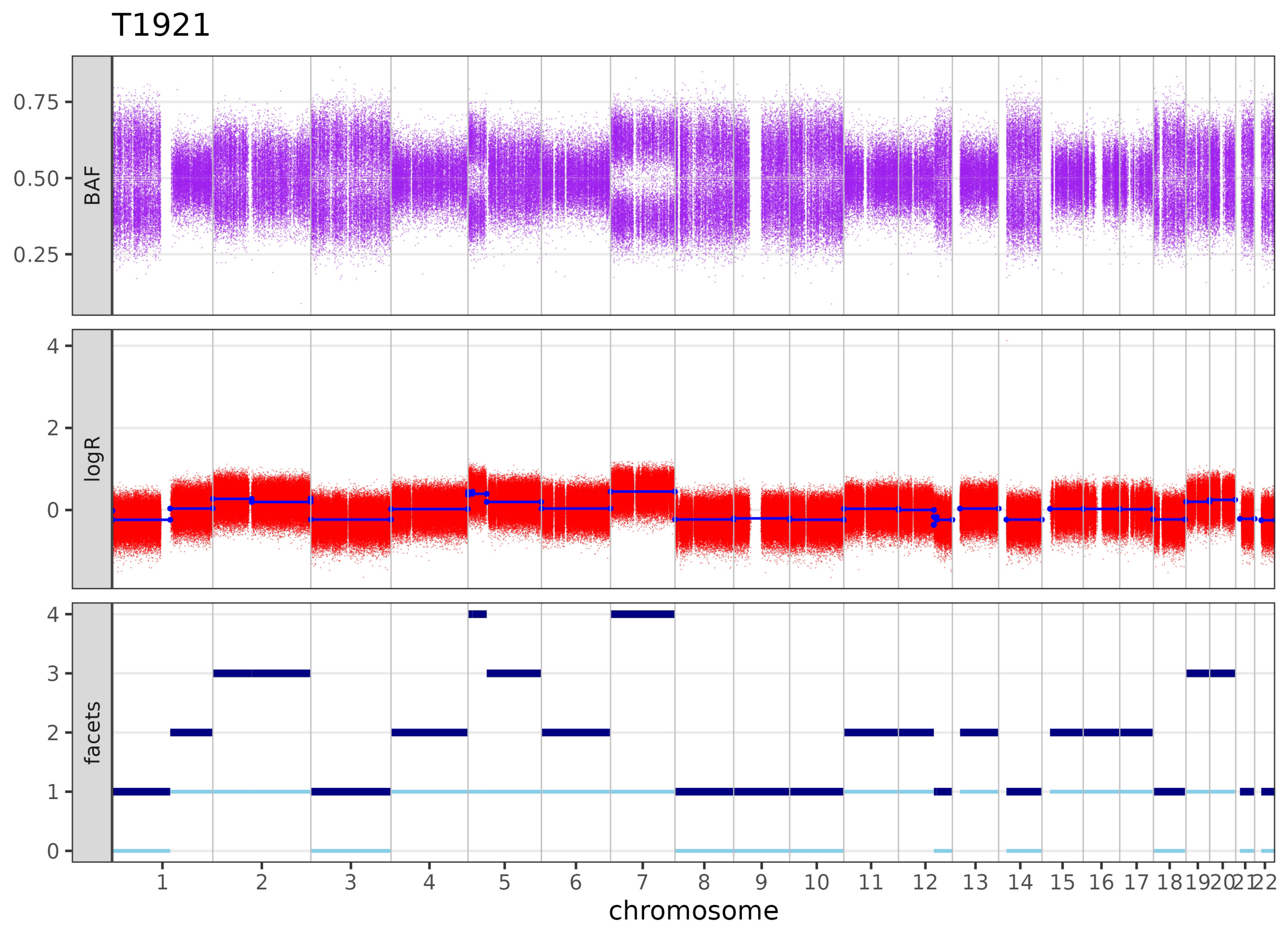
T1921
BAF
0.75
0.50
0.25
logR
4
2
0
facets
4
3
2
1
0
1 2 3 4 5 6 7 8 9 10 11 12 13 14 15 16 17 18 19 20 21 22
chromosome

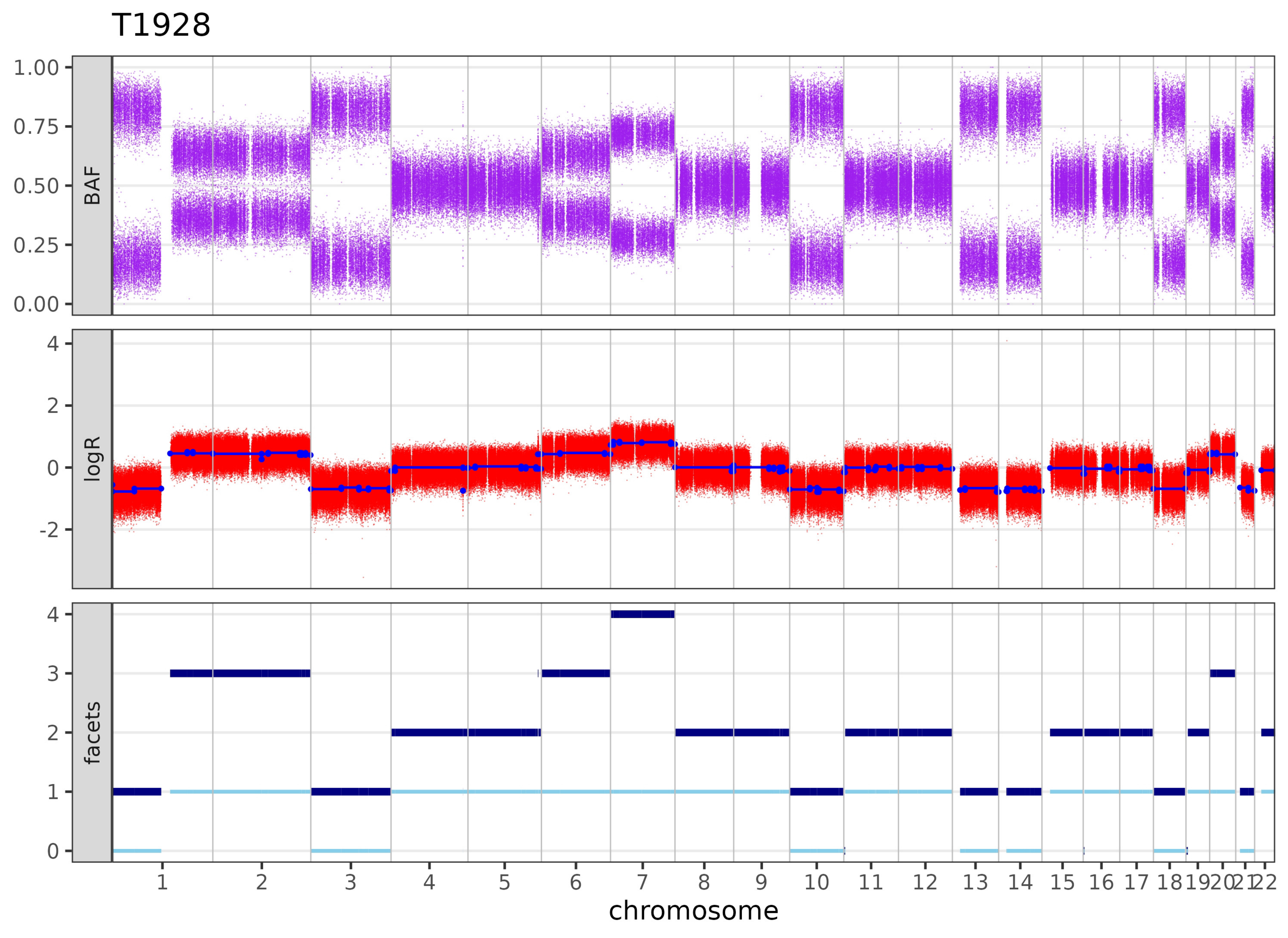

T1928
BAF
logR
facets
1.00
0.75
0.50
0.25
0.00
4
2
0
-2
3
1
1 2 3 4 5 6 7 8 9 10 11 12 13 14 15 16 17 18 19 20 21 22
chromosome

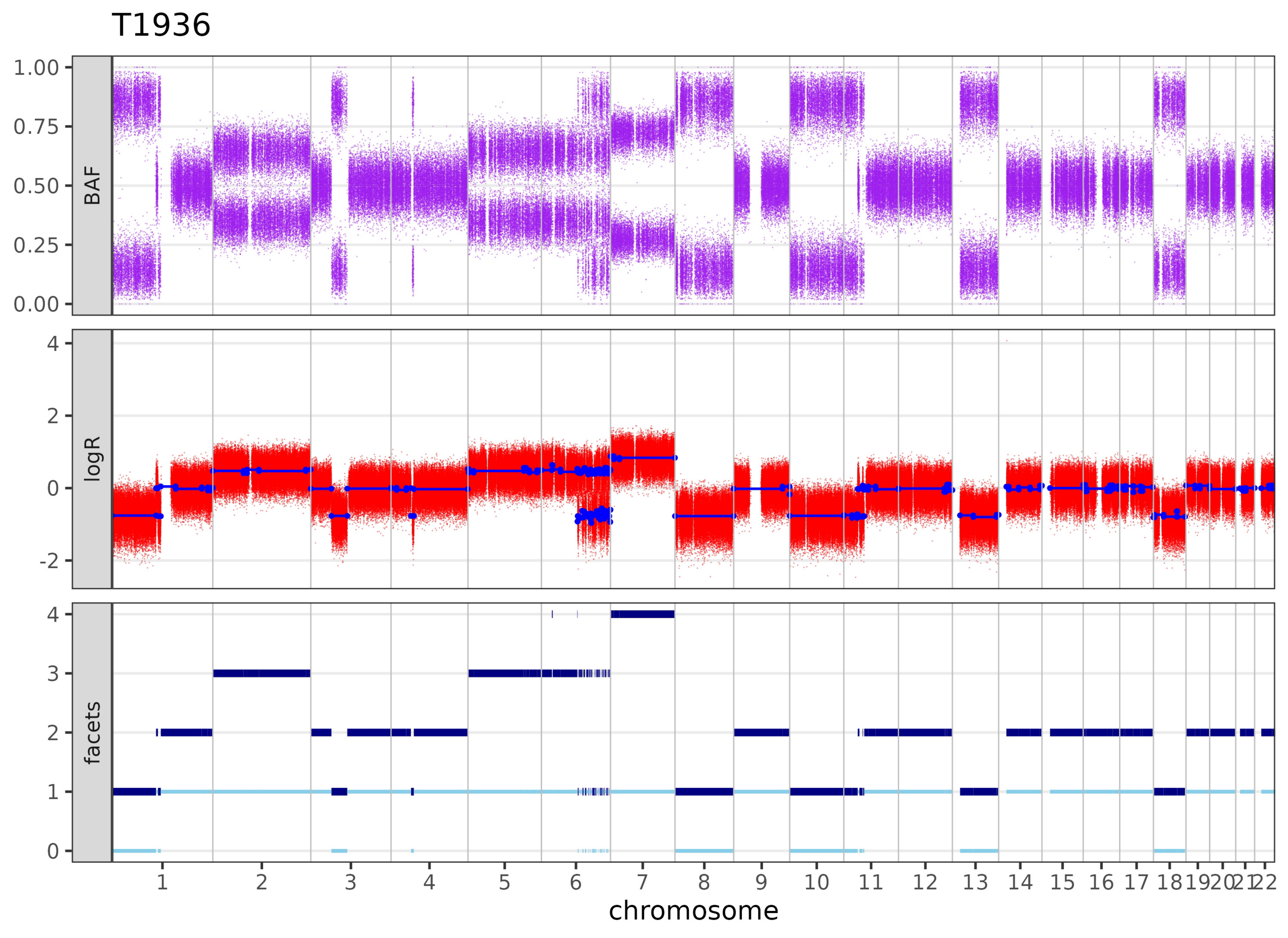

T1936
BAF
1.00
0.75
0.50
0.25
0.00
logR
4
2
0
-2
facets
4
3
2
1
0
1
2
3
4
5
6
7
8
9
10
11
12
13
14
15
16
17
18
19
20
21
22
chromosome

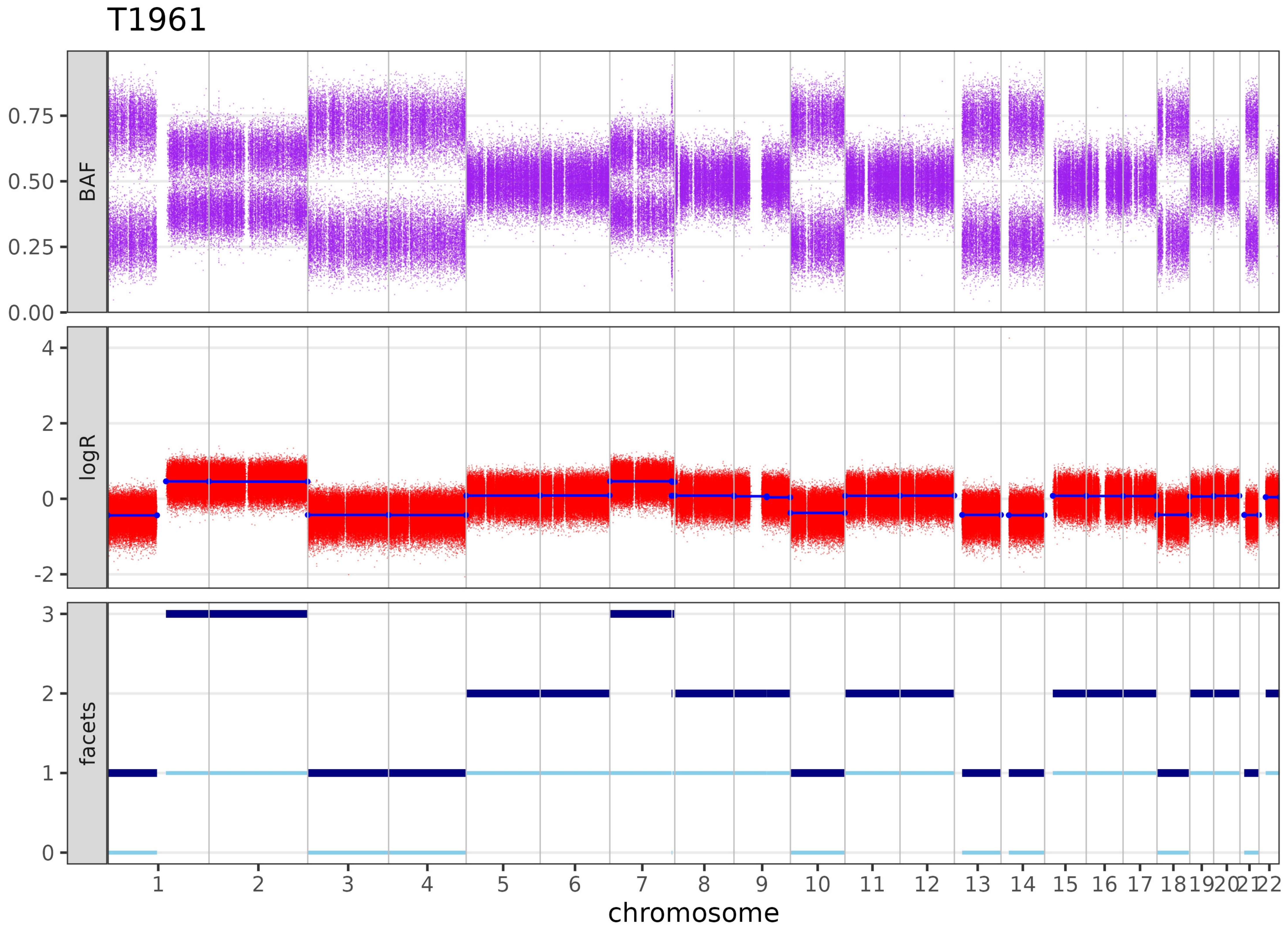
T1961
BAF
logR
facets
0.00
0.25
0.50
0.75
4
2
0
-2
3
2
1
0
1
2
3
4
5
6
7
8
9
10
11
12
13
14
15
16
17
18
19
20
21
22
chromosome

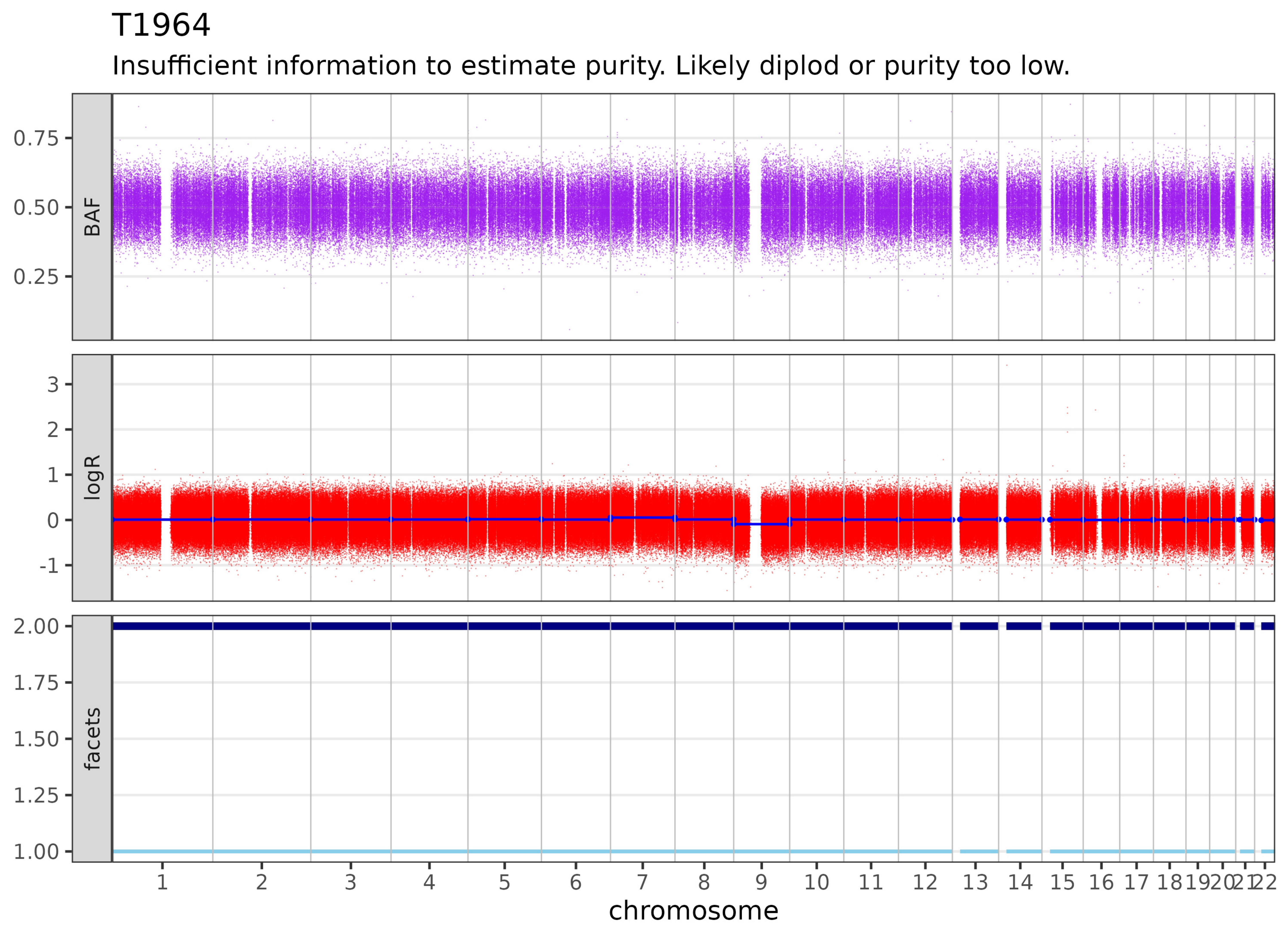
T1964
Insufficient information to estimate purity. Likely diplod or purity too low.
BAF
0.75
0.50
0.25
logR
3
2
1
0
-1
facets
2.00
1.75
1.50
1.25
1.00
1
2
3
4
5
6
7
8
9
10
11
12
13
14
15
16
17
18
19
20
21
22
chromosome

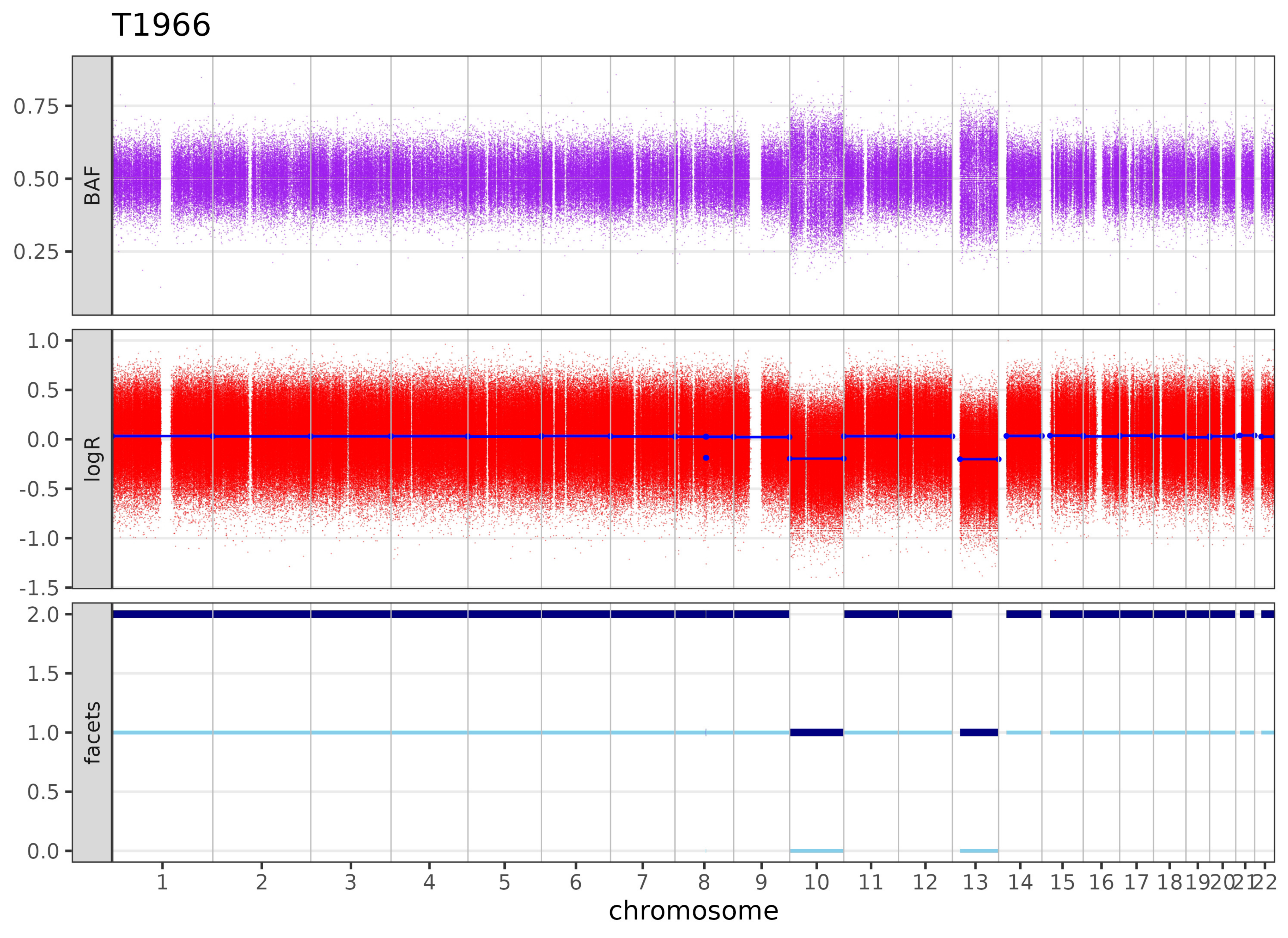
T1966
BAF
0.75
0.50
0.25
logR
1.0
0.5
0.0
-0.5
-1.0
-1.5
facets
2.0
1.5
1.0
0.5
0.0
1
2
3
4
5
6
7
8
9
10
11
12
13
14
15
16
17
18
19
20
21
22
chromosome

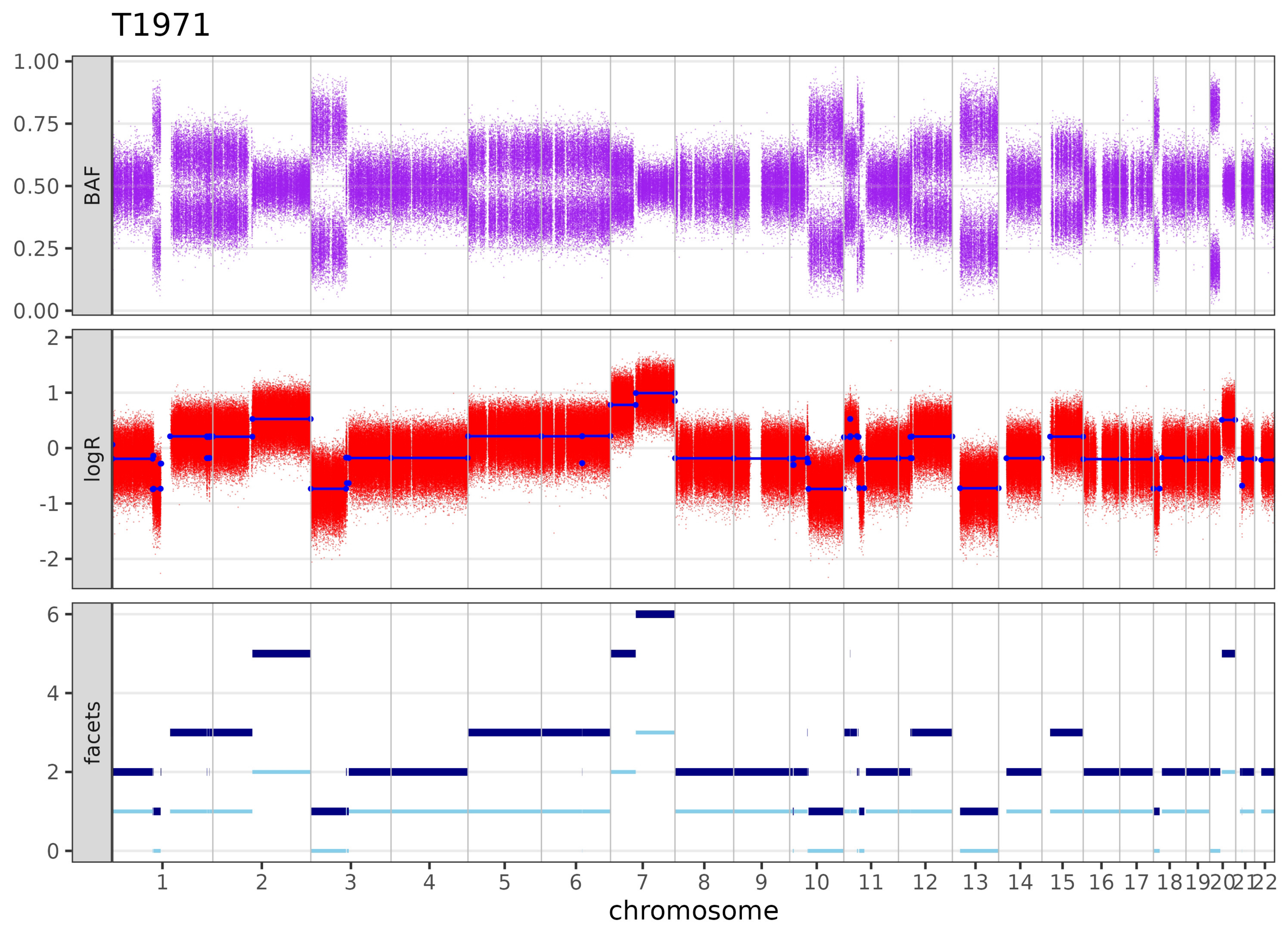

T1971
BAF
logR
facets
1.00
0.75
0.50
0.25
0.00
2
1
0
-1
-2
6
4
2
0
1 2 3 4 5 6 7 8 9 10 11 12 13 14 15 16 17 18 19 20 21 22
chromosome

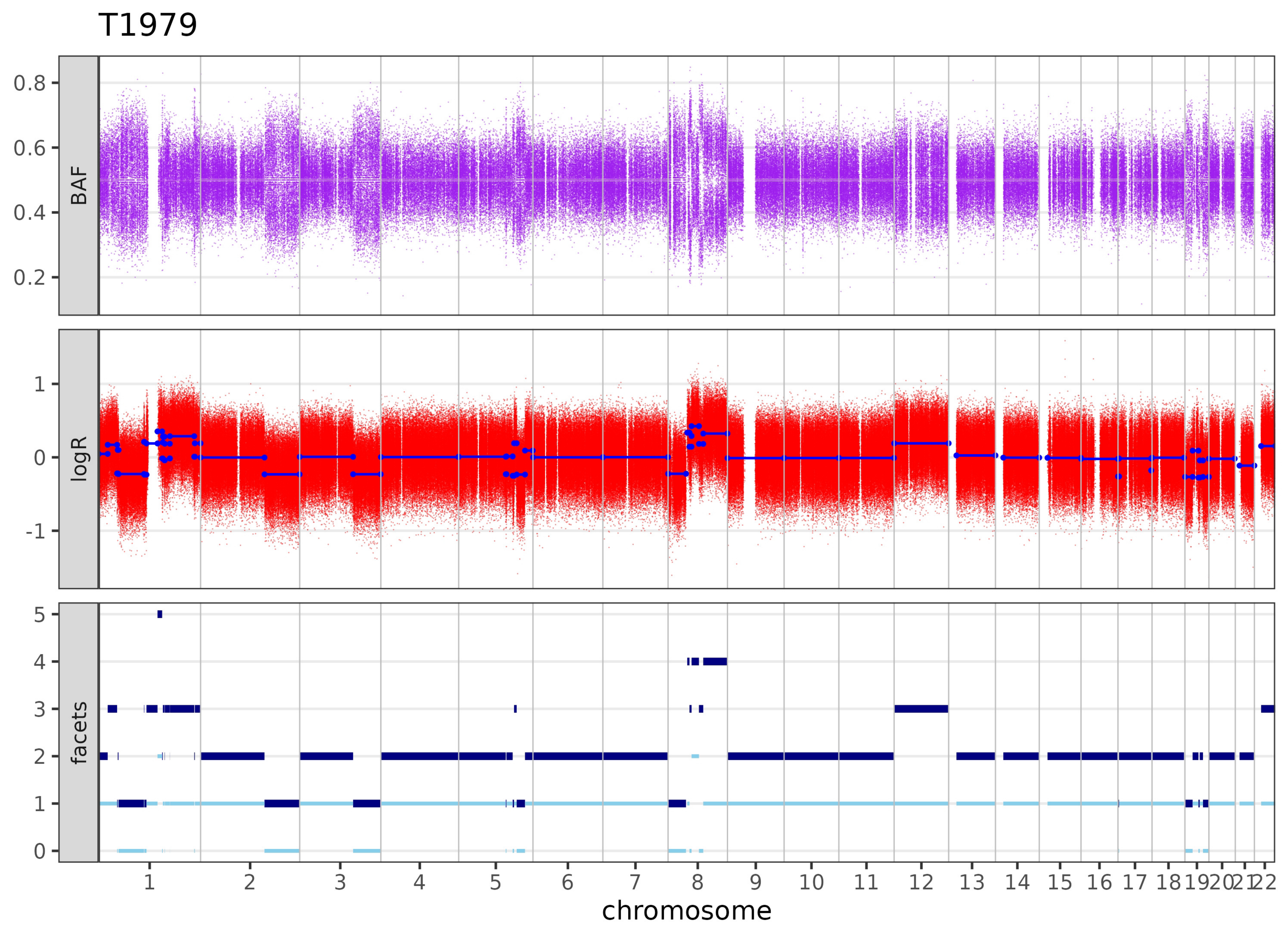

T1979
BAF
0.8
0.6
0.4
0.2
logR
1
0
-1
facets
5
4
3
2
1
0
1 2 3 4 5 6 7 8 9 10 11 12 13 14 15 16 17 18 19 20 21 22
chromosome

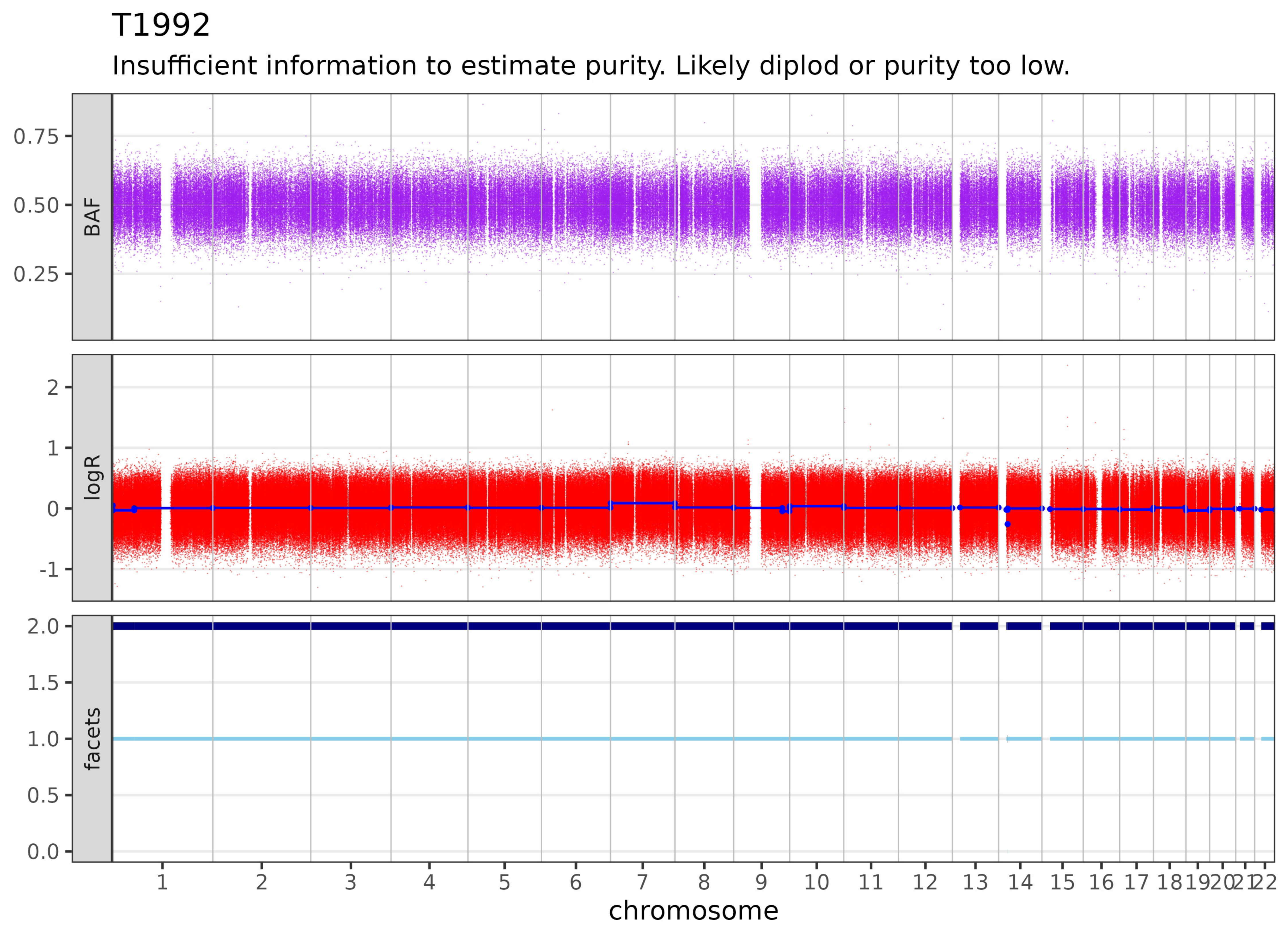
T1992
Insufficient information to estimate purity. Likely diplod or purity too low.
BAF
0.75
0.50
0.25
logR
2
1
0
-1
facets
2.0
1.5
1.0
0.5
0.0
1
2
3
4
5
6
7
8
9
10
11
12
13
14
15
16
17
18
19
20
21
22
chromosome

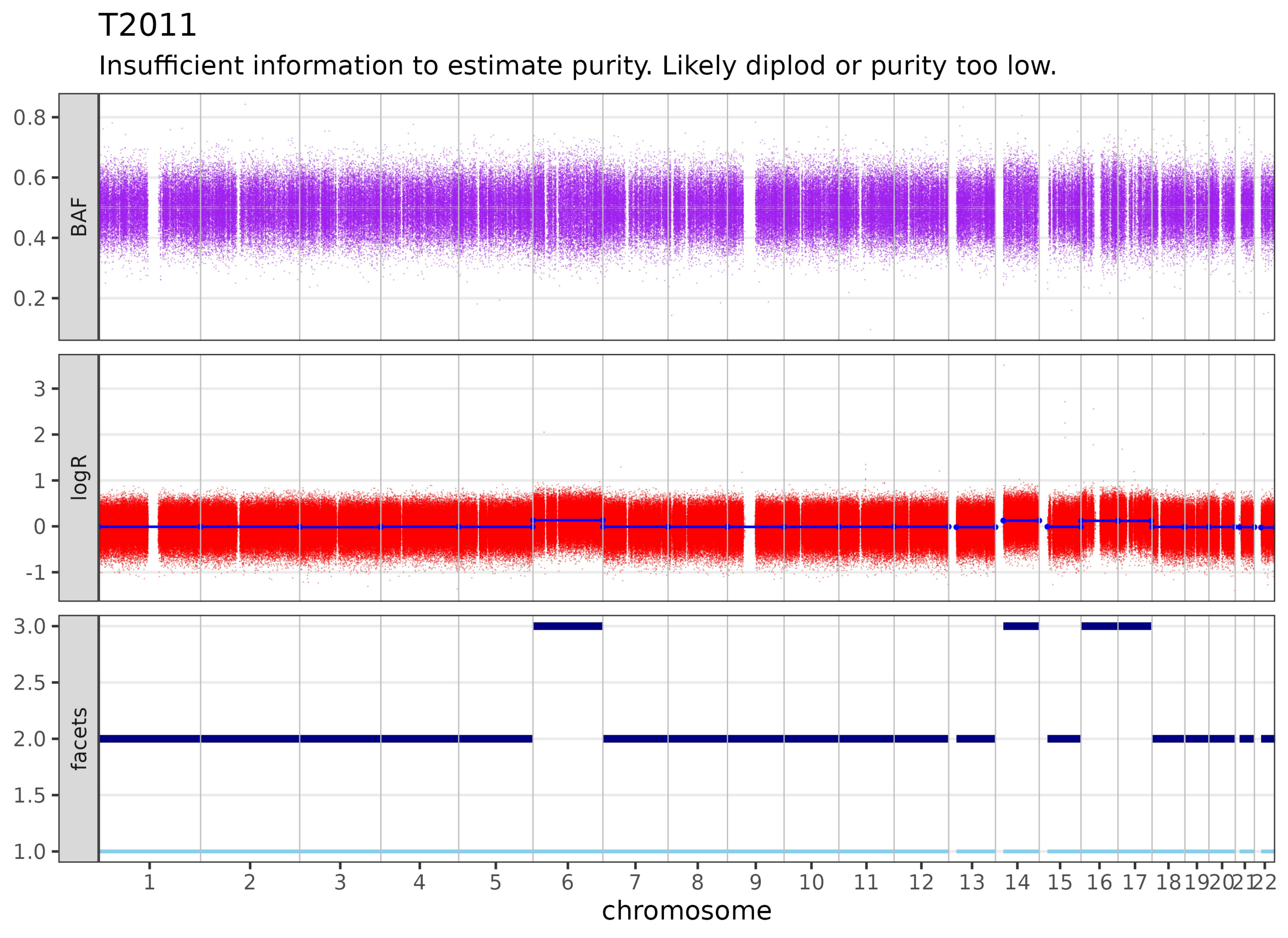
T2011
Insufficient information to estimate purity. Likely diplod or purity too low.
BAF
logR
facets
chromosome
0.8
0.6
0.4
0.2
3
2
1
0
-1
3.0
2.5
2.0
1.5
1.0
1 2 3 4 5 6 7 8 9 10 11 12 13 14 15 16 17 18 19 20 21 22

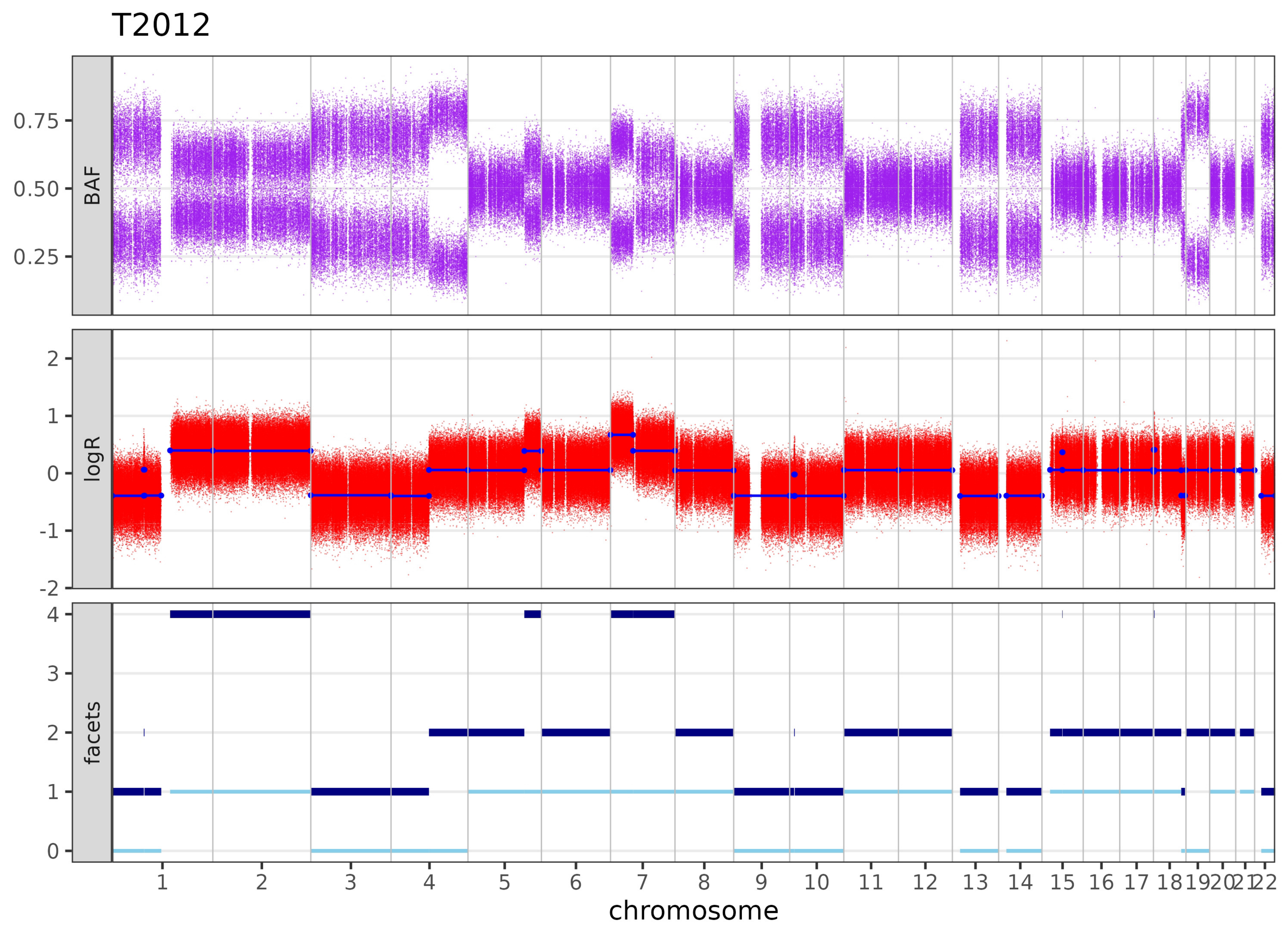

T2012
BAF
0.75
0.50
0.25
logR
2
1
0
-1
-2
facets
4
3
2
1
0
1 2 3 4 5 6 7 8 9 10 11 12 13 14 15 16 17 18 19 20 21 22
chromosome

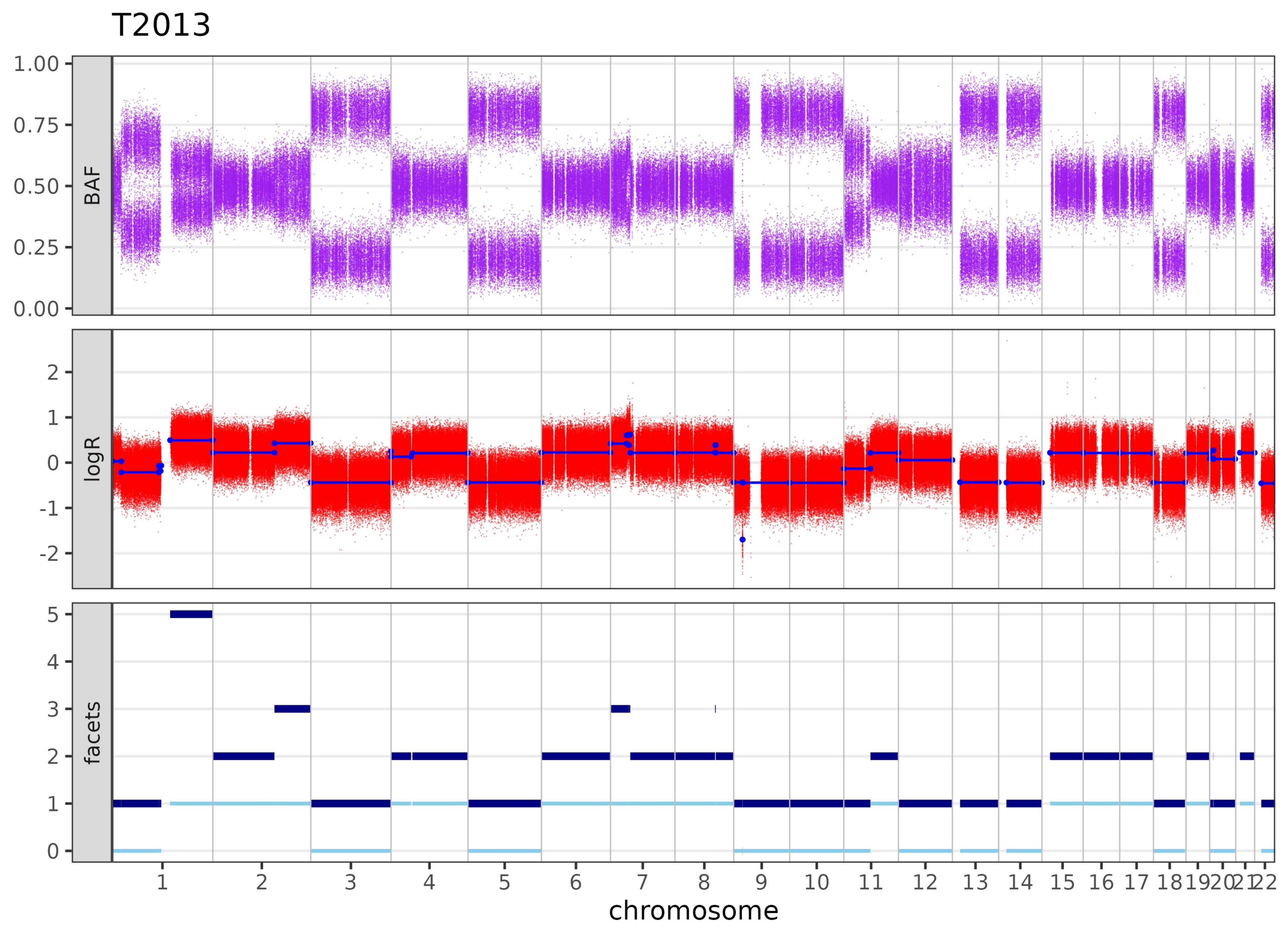
T2013
BAF
logR
facets
chromosome
1.00
0.75
0.50
0.25
0.00
2
1
0
-1
-2
5
4
3
2
1
0
1
2
3
4
5
6
7
8
9
10
11
12
13
14
15
16
17
18
19
20
21
22

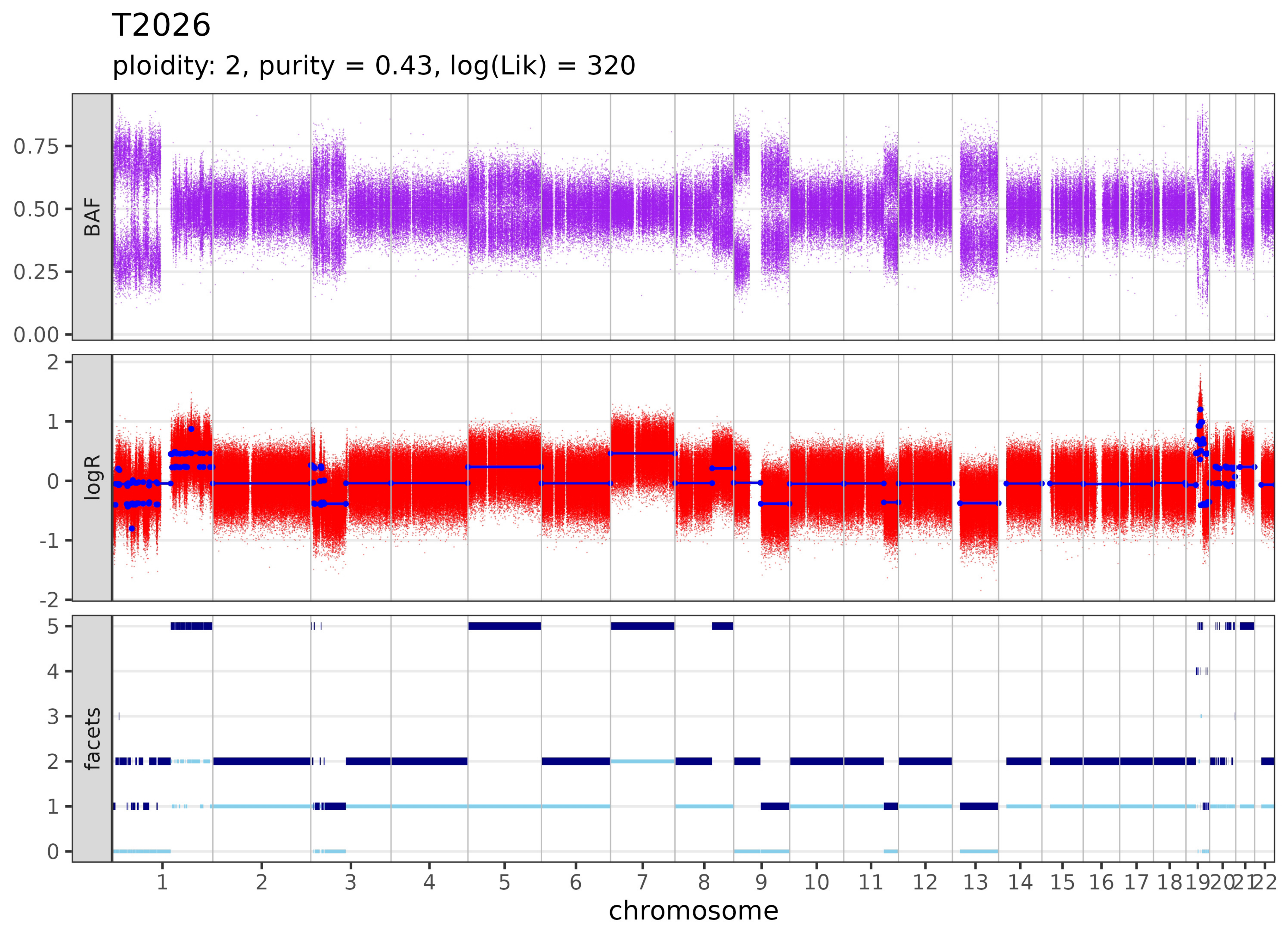

T2026
ploidity: 2, purity = 0.43, log(Lik) = 320
BAF
0.75
0.50
0.25
0.00
logR
2
1
0
-1
-2
facets
5
4
3
2
1
0
1 2 3 4 5 6 7 8 9 10 11 12 13 14 15 16 17 18 19 20 21 22
chromosome

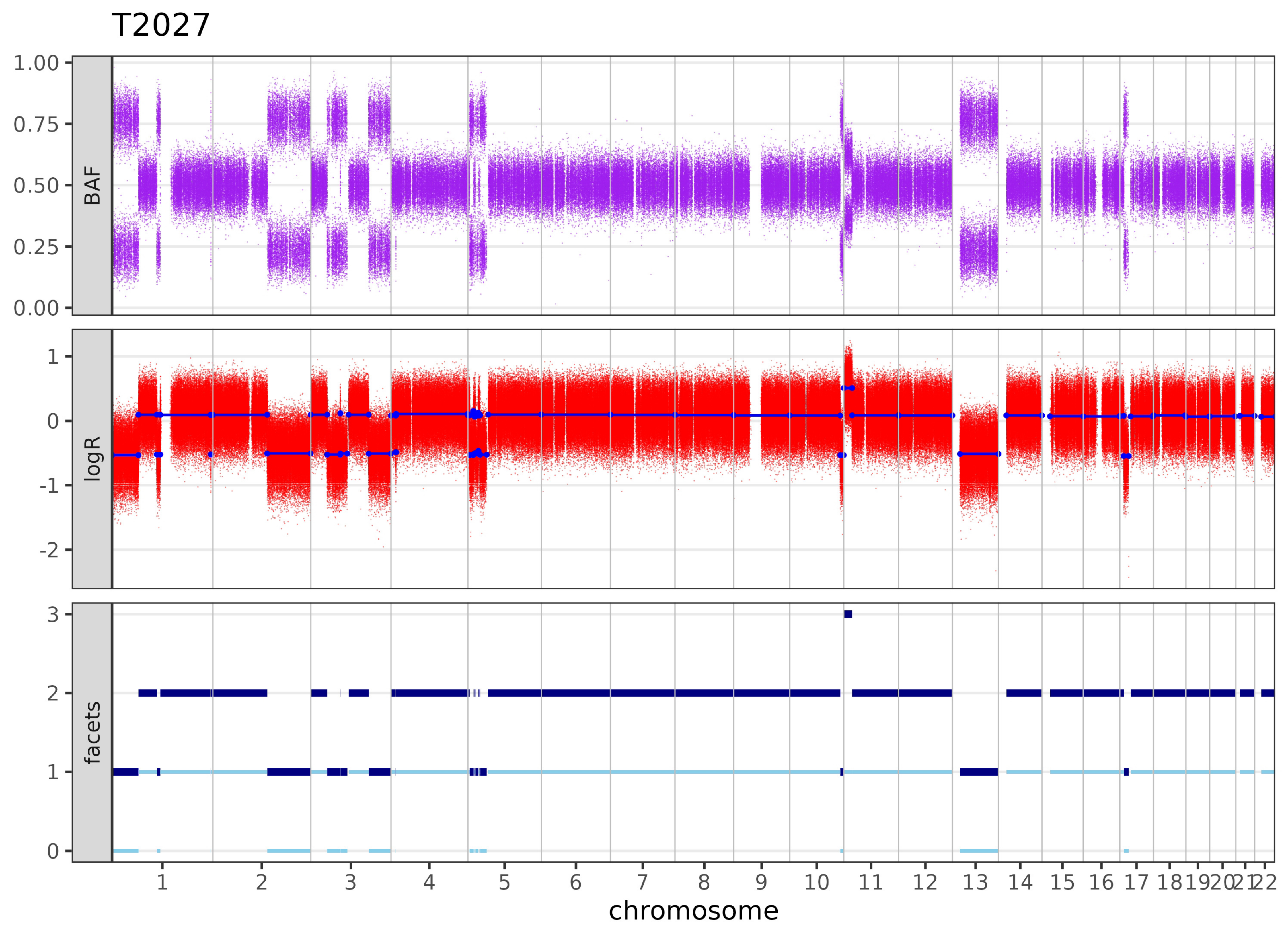
T2027
BAF
1.00
0.75
0.50
0.25
0.00
logR
1
0
-1
-2
facets
3
2
1
0
1 2 3 4 5 6 7 8 9 10 11 12 13 14 15 16 17 18 19 20 21 22
chromosome

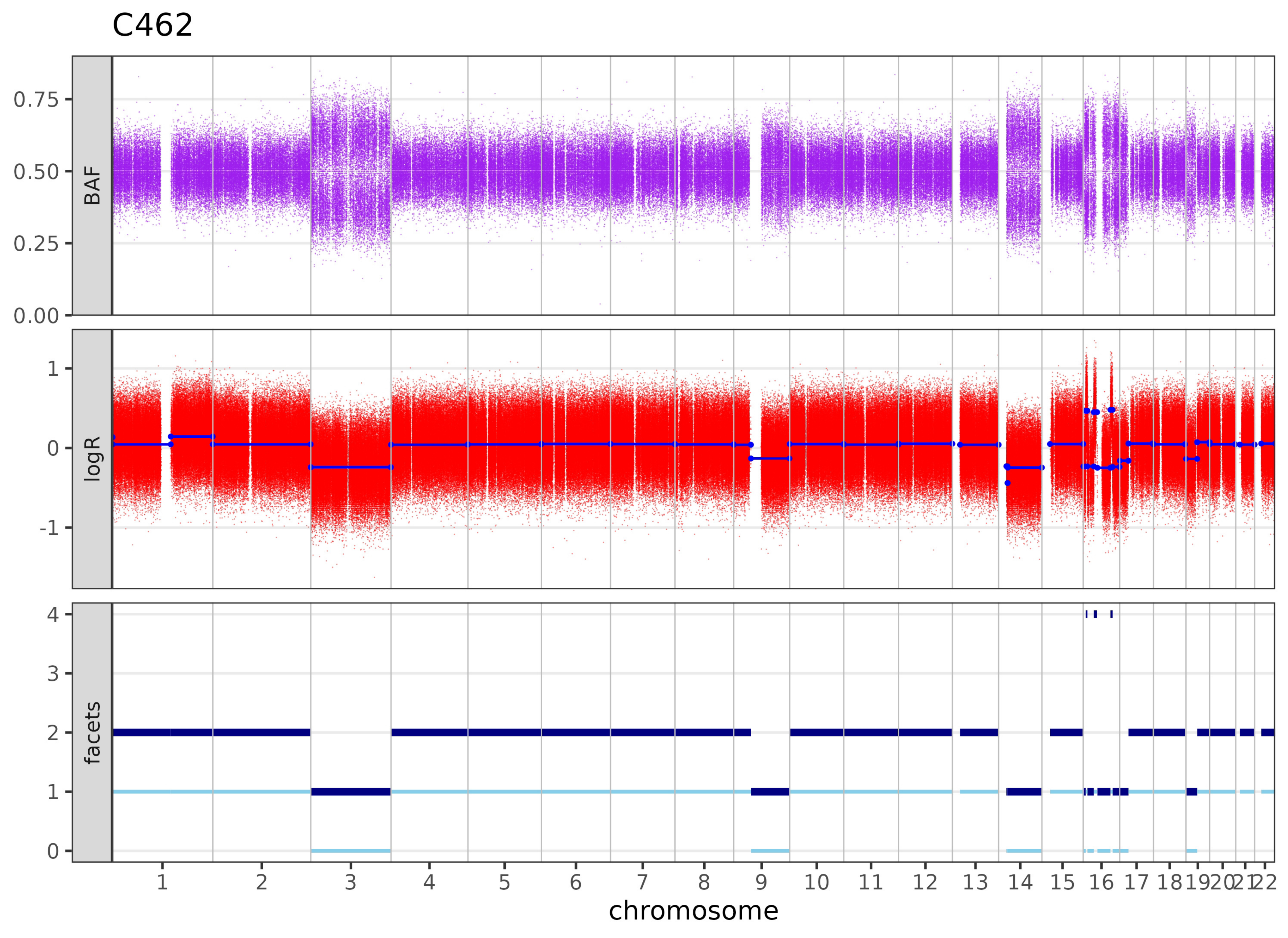

C462
BAF
logR
facets
0.00
0.25
0.50
0.75
-1
0
1
0
1
2
3
4
1 2 3 4 5 6 7 8 9 10 11 12 13 14 15 16 17 18 19 20 21 22
chromosome

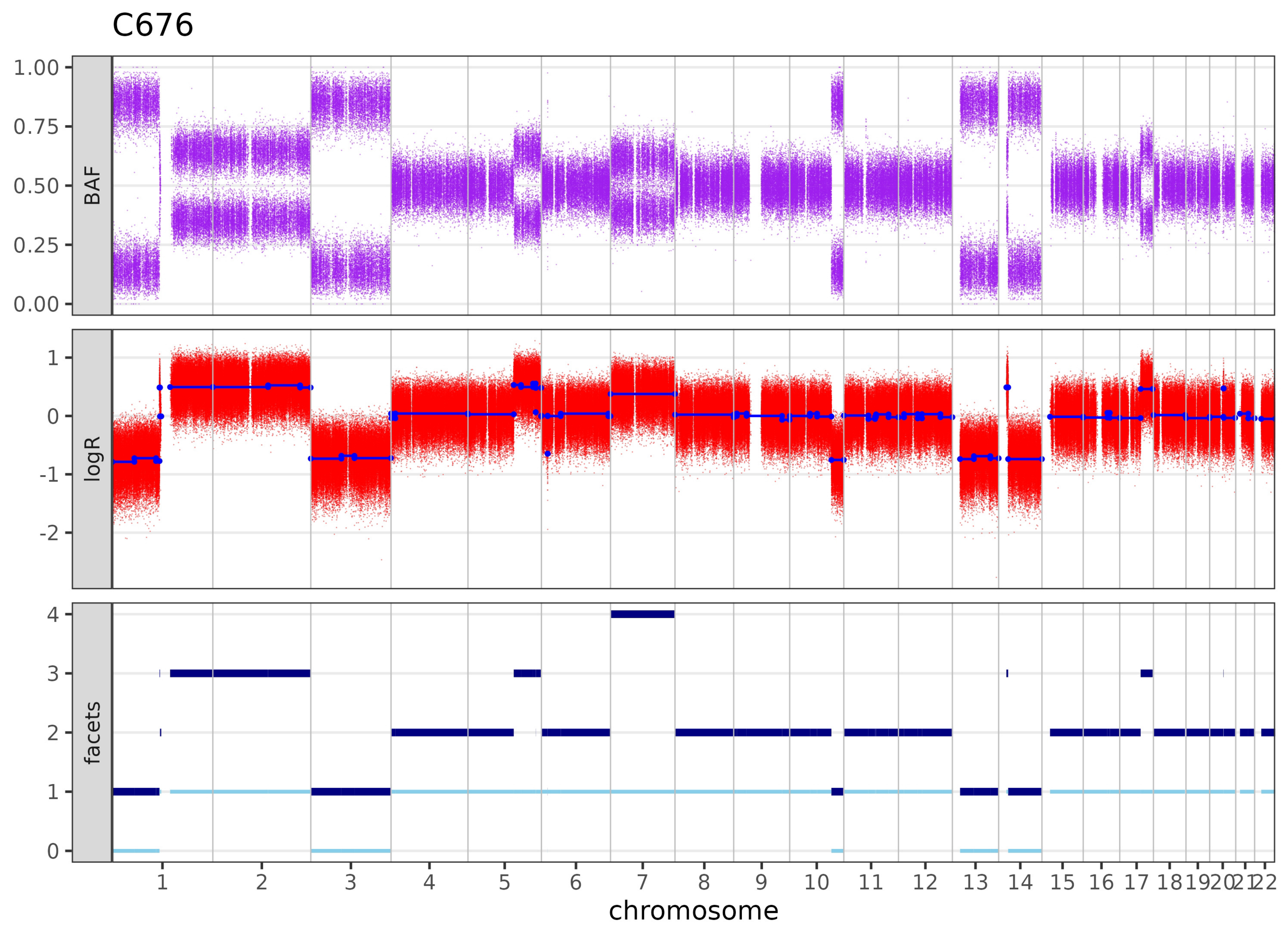

C676
BAF
logR
facets
chromosome
1.00
0.75
0.50
0.25
0.00
1
0
-1
-2
4
3
2
1
0
1 2 3 4 5 6 7 8 9 10 11 12 13 14 15 16 17 18 19 20 21 22

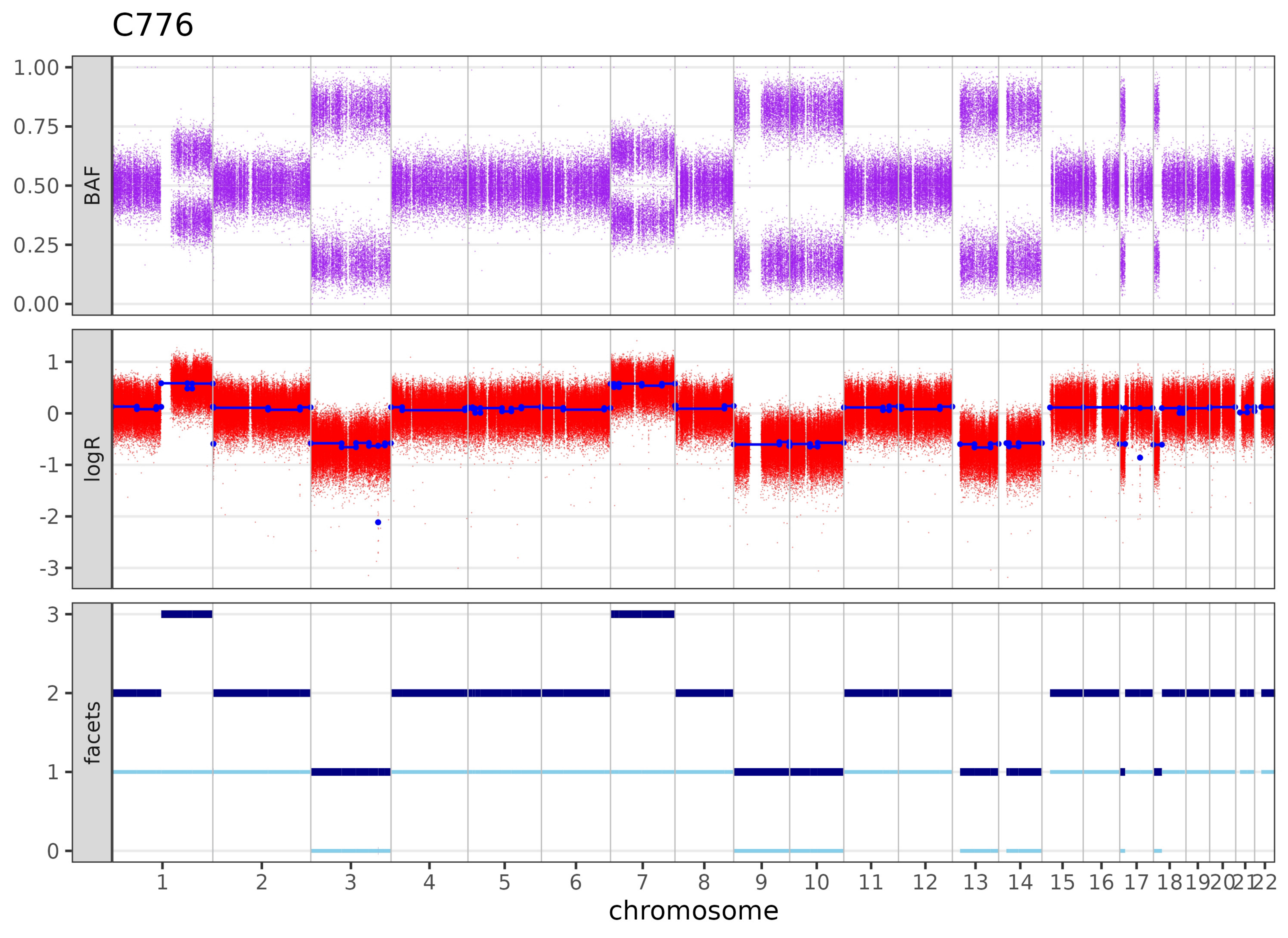
C776
BAF
1.00
0.75
0.50
0.25
0.00
logR
1
0
-1
-2
-3
facets
3
2
1
0
1
2
3
4
5
6
7
8
9
10
11
12
13
14
15
16
17
18
19
20
21
22
chromosome
